# Whole-genome sequencing of *Staphylococcus aureus* isolates after a decade-long decline in MRSA prevalence at a university referral hospital in Guadalajara, Mexico: clonal concentration in MRSA and greater genetic diversity in MSSA

**DOI:** 10.1128/spectrum.00948-26

**Published:** 2026-06-15

**Authors:** Jaime F. Andrade-Villanueva, Pedro Martínez-Ayala, Adolfo Gomez-Quiroz, Violeta Cassandra Vera-Cuevas, Judith Carolina De Arcos-Jiménez, Jaime Briseno-Ramirez

**Affiliations:** 1 Centro Universitario de Ciencias de la Salud, Universidad de Guadalajara 42571 , Guadalajara, Mexico; 2 Antiguo Hospital Civil de Guadalajara “Fray Antonio Alcalde” 103531 , Guadalajara, Mexico; 3 Centro de Investigaciones Biológicas del Noroeste, S. C. 42579 , La Paz, Mexico; 4 Centro Universitario de Tlajomulco, Universidad de Guadalajara 843654, Tlajomulco de Zúñiga, Mexico; 5 Hospital Civil de Oriente, Tonalá, Mexico; Universidad Nacional Autonoma de Mexico - Campus Morelos, Cuernavaca, Mexico; Universidad Nacional Autonoma de Mexico, Mexico City, Mexico

**Keywords:** MRSA, whole-genome sequencing, SCC*mec* excision, clonal dynamics, antimicrobial resistance, *Staphylococcus aureus*

## Abstract

Methicillin-resistant *Staphylococcus aureus* (MRSA) prevalence has declined in many settings, but genomic features of residual MRSA populations remain incompletely defined. We performed whole-genome sequencing (WGS) of 26 *S. aureus* clinical isolates (13 MRSA and 13 methicillin-susceptible *S. aureus* [MSSA]) collected by stratified random sampling during 2024–2025. Analyses included *de novo* assembly, species confirmation, MLST/*spa*/SCC*mec* typing, resistance and virulence profiling, mobile-element analysis, genotype–phenotype concordance, and recombination-filtered/contextual phylogenomics. A secondary matched case-control analysis explored clinical correlates. MRSA was concentrated in ST5-t895-SCC*mec* II (CC5, New York/Japan lineage; 9/13, 69.2%), whereas MSSA was diverse (11 sequence types and 9 clonal complexes; Simpson’s 1 *−* D = 0.974 vs 0.500). SA023 (CC5-MSSA, ST5-t688) nested within the MRSA-CC5 clade and lacked SCC*mec*, a pattern consistent with putative SCC*mec* excision. WGS resolved lineage-specific resistance and mobile-element architectures: CC5-MRSA carried SCC*mec* II with *ermA*/*aadD* and quinolone resistance-determining region mutations, while SCC*mec* IV MRSA belonged to CC8/CC88 and lacked arginine catabolic mobile element/copper and mercury resistance. Panton–Valentine leukocidin genes occurred in six isolates (four community-associated MRSA and two community-associated MSSA). WGS-based resistance prediction showed 91.7% categorical agreement with phenotypes. In the exploratory clinical analysis, prior antibiotic exposure, intensive care unit admission, and healthcare exposure score were associated with MRSA. Contemporary MRSA in this hospital is clonally concentrated, while MSSA is diverse. WGS identifies lineage-specific resistance and mobile element architectures, as well as one observation consistent with SCC*mec* excision, generating hypotheses for longitudinal surveillance rather than establishing a mechanism for MRSA decline. Clinical associations are secondary and exploratory.

**IMPORTANCE:**

Methicillin-resistant *Staphylococcus aureus* (MRSA) infections have declined in many hospitals, but cross-sectional clinical data alone cannot explain the genomic structure of the MRSA populations that remain. This study uses whole-genome sequencing to show that contemporary MRSA isolates from a Mexican university referral hospital are concentrated in a CC5 New York/Japan lineage, whereas methicillin-susceptible *S. aureus* (MSSA) isolates are genetically diverse. The analysis identifies lineage-specific resistance determinants, mobile genetic elements, prophage content, defense systems, and one CC5-MSSA isolate showing a pattern consistent with SCC*mec* excision. These findings provide a descriptive genomic baseline for longitudinal MRSA surveillance. The clinical risk-factor analysis is included as exploratory context, not as the primary evidence for the genomic conclusions.

## INTRODUCTION

*Staphylococcus aureus* is a leading cause of healthcare-associated infections, responsible for an estimated 119,000 bloodstream infections and 20,000 deaths annually in the United States (1). Its clinical spectrum ranges from superficial skin infections to life-threatening bacteremia, endocarditis, and sepsis (2, 3). Methicillin resistance, conferred by *mecA* on the mobile staphylococcal cassette chromosome *mec* (SCC*mec*), has made methicillin-resistant *S. aureus* (MRSA) one of the most consequential antimicrobial-resistant pathogens worldwide (4, 5).

After decades of increasing prevalence, MRSA rates have declined in numerous healthcare settings. In the United States, invasive MRSA infections decreased by 54% between 2005 and 2011 (6). These declines have been attributed to infection prevention bundles, antimicrobial stewardship, and screening programs (7, 8), while genomic and evolutionary processes may shape the MRSA populations that remain. Clonal replacement—whereby dominant epidemic MRSA lineages are displaced by competing lineages—has been documented for multiple pandemic clones (9, 10). Prior experimental studies have reported measurable fitness effects associated with SCC*mec* carriage, including growth-rate disadvantages in some genetic backgrounds; larger SCC*mec* II elements (~53 kb), carrying Tn554 (*ermA*) and pUB110 (*aadD*), are therefore commonly interpreted as imposing a greater predicted metabolic burden than smaller type IV elements (~21–24 kb) (11, 12). Spontaneous SCC*mec* excision, mediated by the *ccrA*/*ccrB* recombinase, has been documented in multiple lineages as a mechanism for MRSA-to-MSSA reversion (13–15).

In Latin America, MRSA prevalence in tertiary hospitals has historically ranged from 30% to 50%, with CC5 and CC8 as the dominant clonal complexes (16, 17). In Mexico, the New York/Japan clone (ST5-t895-SCC*mec* II) is the predominant healthcare-associated MRSA (HA-MRSA) lineage (18, 19), while community-associated MRSA (CA-MRSA) strains of the CC8 background, including USA300-related lineages, have emerged with increasing frequency (20, 21). However, whole-genome sequencing (WGS) studies from Mexico remain scarce, and genomic features associated with local MRSA trends have not been characterized in detail.

Consistent with these global trends, MRSA prevalence at our institution has declined from 28.1% to 14.0% over 9.5 years, with a 50% reduction in incidence density (22), whereas Latin American multicenter data continue to document substantial MRSA burden and regional heterogeneity (16, 17). Despite growing epidemiological evidence of changing MRSA prevalence in the region, the genomic architecture of contemporary low-prevalence MRSA populations remains incompletely described. The present study addresses this gap through WGS of *S. aureus* clinical isolates from the current low-prevalence period (2024–2025) with three objectives: (i) characterize the clonal structure of the contemporary MRSA and methicillin-susceptible *S. aureus* (MSSA) populations; (ii) identify resistance, virulence, and genomic determinants related to cassette burden and colonization that may contextualize the contemporary post-decline population; and (iii) assess evolutionary relationships through recombination-filtered phylogenomics contextualized against global reference genomes.

## MATERIALS AND METHODS

### Study design and setting

This cross-sectional genomic study was conducted at the Antiguo Hospital Civil de Guadalajara “Fray Antonio Alcalde,” a 1,000-bed tertiary-care university referral hospital affiliated with the Universidad de Guadalajara, serving a predominantly uninsured population in Jalisco state, particularly metropolitan Guadalajara (population ~5 million). The sampling design specified *a priori* the selection of 13 MRSA and 13 MSSA non-duplicate clinical isolates by stratified random sampling from the institutional strain collection during 2024–2025, with temporal balance ensured by selecting approximately equal numbers from each year (7 MRSA and 6 MSSA from 2024; 6 MRSA and 7 MSSA from 2025). Randomization within each stratum was performed using computer-generated random numbers, representing the post-peak period of declining MRSA prevalence (22). This stratified design ensured balanced representation of both populations for genomic comparison, while random selection within each stratum minimized sampling bias. Although the sample size is modest, whole-genome sequencing at adequate depth (median estimated coverage, 62.5×; Table S1) provides nucleotide-level resolution across the chromosome for each isolate, enabling comprehensive characterization of dominant lineages, resistance and virulence determinants, and phylogenetic relationships that conventional molecular typing cannot achieve. Source specimens included blood cultures, abscess and tissue samples, respiratory specimens, urine, and biopsies. Patient identifiers were anonymized using study codes SA001–SA027. One isolate (SA003) was retrospectively identified as a nasal colonization screening culture rather than a clinical infection specimen; it was excluded and replaced by an additional randomly selected isolate to maintain the target sample size.

### DNA extraction, library preparation, and sequencing

Isolates were subcultured overnight on blood agar at 37°C. To facilitate lysis of the peptidoglycan-rich gram-positive cell wall, a pre-treatment step was performed: one to two colonies (~3 mm diameter) were resuspended in 80 μL lysozyme (Thermo Scientific, Ref. 900082; 20 mg/mL in 50 mM Tris-HCl [pH 8.0], 50 mM EDTA, and 1.2% Triton X-100) and 120 μL Tris buffer (10 mM Tris-HCl [pH 8.0]), followed by incubation at 37°C for 75 min. Subsequent lysis and protein digestion were carried out by adding 100 μL of the proprietary MagDEA Dx SV lysis buffer (Precision System Science, kit Ref. E1300; manufacturer-stated composition: chaotropic guanidinium thiocyanate-based denaturant with anionic detergent and EDTA-buffered carrier) and 20 μL of Proteinase K (20 mg/mL), with incubation at 56°C for 30 min. Total nucleic acids were extracted using the GeneSelect platform (magLEAD 12gC LiNK, Precision System Science) with the MagDEA Dx SV kit (Ref. E1300; protocol v1.0), based on magnetic bead purification, with a final elution volume of 50 μL.

DNA was quantified by fluorometry using a Qubit 4 fluorometer (Thermo Fisher Scientific) with the dsDNA High Sensitivity assay kit and normalized to 10 ng input per sample.

Sequencing libraries were prepared using the Illumina DNA Prep kit (tagmentation-based) with IDT for Illumina Nextera DNA UD Indexes Set A (10 bp unique dual indexes), using 10 cycles of PCR amplification. Library size distribution and quality were assessed using the QIAxcel system (Qiagen) with a High Sensitivity kit, showing a mean fragment size of ~370 bp.

Libraries were individually quantified, pooled equimolarly at 14 pM, and sequenced on an Illumina MiSeq platform using paired-end mode (2 × 200 bp).

### Strain isolation, phenotypic identification, and MRSA/MSSA classification

Clinical isolates were recovered as part of the routine diagnostic workflow at the Antiguo Hospital Civil de Guadalajara microbiology laboratory. Specimens were inoculated on 5% sheep blood agar and incubated at 35°C in ambient air for 18–24 h. Colonies displaying typical *S. aureus* morphology (creamy yellow, β-hemolytic, and catalase-positive) were subjected to species identification using a combination of conventional biochemistry (slide and tube coagulase, DNase) and the VITEK 2 GP card (bioMérieux). Confirmed *S. aureus* isolates were preserved in 20% glycerol at −80°C in the institutional strain bank. For the purpose of MRSA/MSSA classification, every isolate underwent two independent phenotypic tests interpreted per CLSI 2024 breakpoints (23): (i) cefoxitin disk diffusion (30 μg disk on Mueller-Hinton agar; resistant if zone diameter ≤21 mm) and (ii) oxacillin broth microdilution by VITEK 2 (resistant if MIC ≥4 µg/mL); concordant resistance in both tests defined MRSA, while concordant susceptibility defined MSSA. Discrepant phenotypes (e.g., the borderline oxacillin-resistant *S. aureus* [BORSA] candidate SA020) were resolved by reference to the genotypic *mecA*/*mecC* status, with reads back-mapped against *mecA* (GenBank AB245468.1) as a final confirmation step. *S. aureus* ATCC 29213 (cefoxitin-susceptible) and ATCC 43300 (cefoxitin-resistant, *mecA*+) were used as quality-control strains and included in every weekly run.

### Antimicrobial susceptibility testing and MIC determination

Minimum inhibitory concentrations (MICs) for the panel of agents reported in Table 1 (oxacillin, cefoxitin screen, vancomycin, linezolid, daptomycin, clindamycin, erythromycin, tetracycline, ciprofloxacin, trimethoprim–sulfamethoxazole, gentamicin, and mupirocin) were determined by broth microdilution using the VITEK 2 AST-P607 panel (bioMérieux) following the manufacturer’s instructions. Cefoxitin disk diffusion (30 μg, BD BBL Sensi-Disc) was performed in parallel on Mueller-Hinton agar with a 0.5 McFarland inoculum, and zone diameters were read at 16–18 h, interpreted per CLSI document M100, 34th edition (23) as susceptible (≥22 mm) or resistant (≤21 mm). Inducible MLS_B_ resistance was screened by D-test on Mueller-Hinton agar (clindamycin and erythromycin disks placed 15–26 mm apart). Mupirocin susceptibility was evaluated by E-test (bioMérieux Etest M.U.P.) with high-level resistance defined as MIC ≥512 µg/mL. Quality control included *S. aureus* ATCC 29213 and *Enterococcus faecalis* ATCC 29212 in each batch; results were accepted only when QC values fell within the CLSI-published acceptable ranges.

**TABLE 1:** Molecular typing, antimicrobial susceptibility, resistance determinants, virulence gene content, fitness markers, plasmid content, and clinical characteristics of 26 *Staphylococcus aureus* clinical isolates^*a*^^,^^*j*^^,^^*k*^^,^^*l*^^,^^*m*^

									MIC (µg/mL)										Resistance genes												Clinical			
Isolate	Status	ST	CC	spa	SCCmec	agr	PVL	IEC	PEN	OXA	ERY	CLI	CIP	TET	SXT	LZD	DAP	VAN	*blaZ*	*ermA*	*ermC*	QRDR	*tetK*	*aacA-aphD*	*aadD*	*mupA*	Vir	Fit	Plsm	Adeq	Specimen	Syndrome	Department	Outcome
MRSA—CC5-SCC*mec* II (*n* = 9)																																		
SA001	MRSA	5^*b*^	CC5	t895	II	II	*−*	A	≥0.5	≥4	≥8	≥4	≥8	≤1	≤10	2	0.25	1	+	+	*−*	+	*−*	+	+	*−*	74	H	6	No	Blood	RTI	Geriatrics	Deceased
SA002	MRSA	5	CC5	t895	II	II	*−*	A	≥0.5	≥4	≥8	≥4	≥8	≤1	≤10	4	0.5	≤0.5	+	+	*−*	+	*−*	+	+	*−*	78	H	4	No	Urine	Other	ED	Alive
SA004	MRSA	5	CC5	t895	II	II	*−*	A	≥0.5	≥4	≥8	≥4	*−*	≥16	≤10	4	0.25	1	+	+	*−*	+	*−*	+	+	*−*	78	H	4	No	Abscess	SSTI	Orthopedics	Alive
SA014	MRSA	5	CC5	t895	II	II	*−*	A	≥0.5	≥4	≥8	≥4	≥8	≤1	≤10	2	0.5	1	+	+	*−*	+	*−*	+	+	*−*	78	H	4	No	Respiratory	RTI	Geriatrics	Alive
SA015	MRSA	5^*b*^	CC5	t895	II	II	*−*	A	0.12	≤0.25^*h*^	≤0.25	≥4	—	≤1	≤10	2	—	1	+	+	*−*	*−*	*−*	+	+	*−*	78	H	—	No	Blood	RTI	Nephrology	Deceased
SA019	MRSA	5	CC5	t895	II	II	*−*	A	≥0.5	≥4	≥8	≥4	≥8	≤1	≤10	2	≤0.5	≤0.5	+	+	*−*	+	*−*	+	+	*−*	78	H	4	No	Blood	EVI	Plastic Surgery	Deceased
SA021	MRSA	5	CC5	t895	II	II	*−*	A	≥0.5	≥4	≥8	≥4	≥8	2	≤10	2	0.25	≤0.5	+	+	*−*	+	*−*	+	+	*−*	78	H	4	No	Blood	IAI	General Surgery	Alive
SA024	MRSA	5	CC5	t895	II	II	*−*	A	≥0.5	≥4	≥8	≥4	≥8	≤1	≤10	2	0.5	≤0.5	+	+	*−*	+	*−*	+	+	*−*	78	H	4	No	Respiratory	EVI	Plastic Surgery	Deceased
SA026	MRSA	5	CC5	t895	II	II	*−*	A	≥0.5	≥4	≥8	≥4	≥8	≤1	≤10	2	0.25	1	+	+	*−*	+	*−*	+	+	*−*	78	H	4	No	Urine	UTI	Oncology	Deceased
SA006	MRSA	8	CC8	t008	IV	I	+	E	≥0.5	≥4	≥8	≥4	≤0.5	≤1	≤10	2	0.25	1	+	*−*	+	+	*−*	*−*	*−*	*−*	80	L	4	No	Abscess	SSTI	Orthopedics	Alive
SA018	MRSA	8	CC8	t008	IV	I	+	A	≥0.5	≥4	≥8	≥4	≥8	≥16	20	2	0.25	1	+	*−*	+	+	+	+	*−*	+	82	L	5	No	Abscess	SSTI	ENT	Alive
SA027	MRSA	8	CC8	t008	IV	I	+	E	≥0.5	0.5^*h*^	≤0.25	0.25	≤0.5	≤1	≤10	2	0.25	≤0.5	+	*−*	+	*−*	*−*	*−*	*−*	*−*	72	L	4	No	Biopsy	SSTI	Burns	Alive
SA005	MRSA	88	CC88	t13831	IV	III	+	A	≥0.5	≥4	≥8	≥4	≥8	≤1	≤10	2	0.5	≤0.5	+	*−*	+	*−*	+	+	+	*−*	82	L	7	No	Abscess	SSTI	HIV Unit	Alive
SA007	MSSA	152	CC152	t1096	−	I	+	+	≥0.5	0.5	≤0.25	0.25	≤0.5	≤1	≤10	2	0.5	≤0.5	+	*−*	*−*	*−*	*−*	*−*	*−*	*−*	81	*−*	3	Yes	Tissue	SSTI	Burns	Alive
SA008	MSSA	22	CC22	t223	*−*	I	*−*	+	≥0.5	≤0.25	≤0.25	0.25	≤0.5	≤1	≤10	2	0.25	≤0.5	+	*−*	*−*	*−*	*−*	*−*	*−*	*−*	69	*−*	3	Yes	Tissue	BJI	Burns	Alive
SA009	MSSA	15	CC15	t085	*−*	II	*−*	+	≥0.5	0.5	≤0.25	0.25	—	≤1	≤10	2	0.25	≤0.5	+	*−*	*−*	*−*	*−*	*−*	*−*	*−*	71	*−*	3	Yes	Tissue	BJI	Neurosurgery	Alive
SA010	MSSA	8	CC8	t008	*−*	I	+	+	0.12	0.5	≥8	≥4	≥8	≤1	≤10	2	1	≤0.5	*−*	*−*	+	+	*−*	*−*	*−*	*−*	82	*−*	5	—	Abscess	SSTI	Dermatology	Alive
SA011	MSSA	2867	CC8	t2016	*−*	II	*−*	+	0.06	≤0.25	≤0.25	0.25	≤0.5	≤1	≤10	2	0.5	≤0.5	*−*	*−*	*−*	*−*	*−*	*−*	*−*	*−*	78	*−*	2	Yes	Abscess	SSTI	ED	Alive
SA012	MSSA	*−^c^*	CC5^*d*^	t010	*−*	II	*−*	+	≥0.5	≤0.25	≤0.25	0.25	≤0.5	≤1	≤10	2	0.5	≤0.5	+	*−*	*−*	*−*	*−*	*−*	*−*	*−*	78	*−*	4	Yes	Abscess	BJI	Neurosurgery	Alive
SA013	MSSA	188	CC188	t189	*−*	I	*−*	+	≥0.5	≤0.25	≥8	0.25	≤0.5	≥16	≤10	2	0.5	2	+	*−*	*−*	+	+	+	+	*−*	80	*−*	3	Yes	Tissue	SSTI	Orthopedics	Alive
SA016	MSSA	4552	CC97^*e*^	t224	*−*	I	*−*	+	≥0.5	0.25	≤0.25	0.25	—	≤1	≤10	2	0.5	≤0.5	+	*−*	*−*	*−*	*−*	*−*	*−*	*−*	73	*−*	6	Yes	Blood	VAI	Nephrology	Alive
SA017	MSSA	Novel^*f*^	CC22	t309	*−*	I	*−*	+	≥0.5	0.5	≤0.25	0.25	≤0.5	≤1	≤10	2	0.5	≤0.5	+	*−*	*−*	*−*	*−*	*−*	*−*	*−*	67	*−*	3	Yes	Blood	EVI	Cardiology	Alive
SA020	MSSA	25	CC25	t078	*−*	I	*−*	+	≥0.5	≥4^*g*^	≥8	0.25^*i*^	≤0.5	≤1	≤10	2	0.25	≤0.5	+	*−*	*−*	*−*	*−*	*−*	*−*	*−*	79	*−*	4	Yes	Bone	BJI	Orthopedics	Alive
SA022	MSSA	188	CC188	t189	*−*	I	*−*	+	≥0.5	0.25	≤0.25	0.12	2	≤1	≤10	2	0.5	≤0.5	+	*−*	*−*	+	*−*	+	*−*	*−*	80	*−*	3	Yes	Blood	EVI	Oncology	Deceased
SA023	MSSA	5	CC5	t688	*−*	II	*−*	+	≥0.5	0.5	≤0.25	0.25	2	≤1	≤10	2	0.5	≤0.5	*−*	*−*	*−*	*−*	*−*	+	+	+	57	*−*	3	Yes	Respiratory	RTI	Cardiology	Deceased
SA025	MSSA	30	CC30	t012	*−*	III	*−*	+	≥0.5	0.5	≤0.25	0.25	—	≤1	≤10	2	0.25	≤0.5	+	*−*	*−*	*−*	*−*	*−*	*−*	*−*	75	*−*	4	Yes	Tissue	BJI	Neurosurgery	Alive

^*a*^ ST, sequence type; CC, clonal complex; SCC*mec*, staphylococcal cassette chromosome *mec*; PVL, Panton–Valentine leukocidin; IEC, immune evasion cluster; PEN, benzylpenicillin; OXA, oxacillin; ERY, erythromycin; CLI, clindamycin; CIP, ciprofloxacin; TET, tetracycline; SXT, trimethoprim–sulfamethoxazole; LZD, linezolid; DAP, daptomycin; VAN, vancomycin; QRDR, quinolone resistance-determining region; Vir, virulence gene count; Fit, predicted SCC*mec*-associated metabolic burden category; Plsm, plasmid count; Adeq, adequate empiric therapy; RTI, respiratory tract infection; SSTI, skin and soft tissue infection; EVI, endovascular infection; IAI, intra-abdominal infection; UTI, urinary tract infection; BJI, bone and joint infection; VAI, vascular access infection; ED, emergency department; ENT, ear–nose–throat; —, not tested or not applicable. Bold MIC values denote resistance per CLSI 2024 breakpoints or instrument-reported interpretations: PEN ≥0.25, OXA ≥4, ERY ≥8, CLI ≥4, CIP ≥4, TET ≥16, and SXT 20 µg/mL. CIP MIC of 2 µg/mL = intermediate. All isolates were susceptible to vancomycin (MIC ≤2), linezolid (MIC ≤4), and daptomycin (MIC ≤1); all but SA018 were susceptible to trimethoprim–sulfamethoxazole. No *vanA*/*vanB*, *cfr*/*optrA*, or acquired *dfr* determinants were detected.

^*b*^ SA001 and SA015 showed the canonical ST5 allelic profile (1-4-1-4-12-1-10; Table S5).

^*c*^ Novel ST; nearest match not assigned by multilocus sequence typing (MLST) database.

^*d*^ Assigned to CC5 based on nearest-neighbor eBURST analysis and *spa*-CC concordance.

^*e*^ Assigned to CC97 based on double-locus-variant linkage and *spa*-CC concordance.

^*f*^ Novel ST not previously deposited in the MLST database; allelic profile submitted to PubMLST.

^*g*^ SA020: borderline oxacillin-resistant *S. aureus*; *mecA*/*mecC*-negative with OXA MIC ≥4 µg/mL, attributed to *blaZ* hyperproduction.

^*h*^ SA015 and SA027: phenotypic heteroresistance to oxacillin (*mecA*-positive, cefoxitin screen positive, OXA MIC below breakpoint).

^*i*^ SA020: positive D-test (inducible clindamycin resistance); all others, D-test negative or not applicable.

^*j*^ Number of virulence-associated genes detected out of 94 screened (ABRicate/VFDB, ≥80% identity and coverage). MRSA mean, 77.8 ± 2.8; MSSA mean, 73.1 ± 7.1 (*P* = 0.08, Mann–Whitney *U*).

^*k*^ QRDR mutations detected by AMRFinderPlus: +, *gyrA* S84L and/or *parC* S80F/S80Y; −, wild type.

^*l*^ Fit: predicted SCC*mec*-associated metabolic burden, inferred from cassette type, size, and gene content rather than direct fitness measurement in this study. H, higher predicted burden (type II, 53 kb); L, lower predicted burden (type IV, 24 kb); −, none. All CP8 capsule; arginine catabolic mobile element-negative (Table S12).

^*m*^ Plsm: number of predicted MOB-recon plasmid clusters from final Clinical ID assemblies. Median 4 (range, 2–7) among 25 evaluable assemblies; —, SA015 excluded because fragmented assembly precluded reliable plasmid reconstruction (Table S9).

### Read quality assessment and filtering

Read quality was assessed with FastQC v0.11.9 (24) and aggregated with MultiQC v1.14 (25). Adapter trimming and quality filtering were performed with fastp v1.0.1 (26) using paired-end adapter auto-detection, a qualified-quality threshold of Phred 20, a minimum read length of 50 bp, and overlap-based error correction (minimum overlap, 30 bp; maximum overlap differences, 5; and maximum difference percentage, 20%). Post-filtering quality metrics were summarized with SeqKit v2.8.2 (27); per-isolate sequencing quality metrics are reported in Table S1.

### *De novo* assembly and annotation

Draft genomes were assembled with SPAdes v4.2.0 in standard paired-end mode using the --careful and --only-assembler options (28); contigs shorter than 500 bp were discarded. Assembly quality was evaluated with QUAST v5.3.0 (29). Assemblies were evaluated against expected *S. aureus* genome parameters (size, 2.7–3.1 Mb; GC content, 32%–34%, ≤200 contigs, and *N*_50_ ≥50 kb); assemblies outside these thresholds were retained only when independent species confirmation and contamination screening supported their validity, and their limitations are flagged in Table S22. Species identity was confirmed by whole-genome average nucleotide identity (ANI) using FastANI v1.34 (30); each draft assembly was queried against five *S*. *aureus* reference genomes (NCTC8325, GCF_000013425.1; N315, GCF_000009645.1; USA300 FPR3757, GCF_000013465.1; Mu50, GCF_000010465.1; and MW2, GCF_000011505.1) and *Staphylococcus epidermidis* RP62A (GCF_000011865.1) as outgroup. All 26 assemblies returned ≥97.28% ANI to NCTC8325 (mean, 98.7%; range, 97.28%–99.79%) and ≥95% ANI to at least one *S*. *aureus* reference, while the *S. epidermidis* outgroup yielded no significant ANI (alignment fraction below the FastANI reporting threshold), confirming species identity (per-isolate ANI values in Table S2). Kraken2 v2.17.1 (31) was retained as an orthogonal contamination screen (≥98% reads classified as *S. aureus* for all isolates), and species-specific multilocus sequence typing (MLST) and *spa* typing against the PubMLST *S. aureus* scheme provided two further independent species-identity controls. Genome annotation was performed with Prokka v1.15.6 (32).

### Molecular typing

Multilocus sequence typing was performed with mlst v2.32.2 (T. Seemann, github.com/tseemann/mlst) against the PubMLST *S. aureus* scheme (33). *spa* typing used spaTyper v0.3.3 (34). SCC*mec* typing employed staphopia-sccmec v1.0.0 (35). Clonal complex assignment followed PubMLST eBURST definitions; for isolates with unresolved sequence types, double-locus variant analysis and *spa* type cross-referencing with the Ridom SpaServer (36) were used to infer clonal complex membership. The *agr* group was determined as part of the typing workflow. Complete MLST allelic profiles and inferred clonal-complex assignments are provided in Table S5.

### Antimicrobial resistance and virulence gene profiling

Resistance determinants were identified using AMRFinderPlus v4.2.5 with organism-specific settings for *S. aureus* (including point mutation detection) (37) and ABRicate v1.2.0 querying CARD and ResFinder databases at ≥80% identity and ≥60% coverage thresholds (38–40). A gene was considered present if detected by at least two independent methods. To confirm methicillin resistance at the read level, quality-filtered reads from all MSSA isolates were mapped with BWA-MEM v0.7.19 against *mecA* (GenBank AB245468.1); zero mapped reads confirmed MSSA status (41). Virulence genes were screened with ABRicate against VFDB (≥80% identity and coverage) (42). Immune evasion cluster (IEC) classification followed the established scheme (43). The arginine catabolic mobile element (ACME) and copper and mercury resistance (COMER) element were specifically screened (20, 21).

### Phylogenomic analysis

Reference-based single nucleotide polymorphism (SNP) calling was performed with Snippy v4.6.0 using *S. aureus* USA300 FPR3757 (GenBank GCF_000013465.1) as a reference, with a minimum coverage of 10× and a minimum variant allele fraction of 0.9 (44). A core-genome alignment was generated with snippy-core, retaining only positions present in all isolates. Recombination was detected and masked using Gubbins v3.4.3 (45). Maximum-likelihood phylogeny was inferred with IQ-TREE v2.3.6 (model TVM + F + ASC + R3, 1,000 ultrafast bootstrap replicates) (46). Pairwise SNP distances were computed with snp-dists v0.8.2 (47). A contextual phylogeny was constructed using an expanded data set of 40 taxa comprising the 26 study isolates and 14 globally representative reference genomes (Table S3); maximum-likelihood inference used the GTR + F + R4 model with 1,000 ultrafast bootstrap replicates. Phylogenies were visualized with ggtree (48); tree files and the recombination-filtered core-genome alignment are provided as Data S3 and S4. Pan-genome analysis was performed with Roary v3.13.0 (95% amino acid identity and 99% prevalence threshold for core genes) (49).

### Prophage detection and insertion sequence analysis

Prophage regions were identified using PhiSpy v5.0.2 (50) with the *S. aureus*-specific training set. Virulence genes within prophage regions were annotated by cross-referencing Prokka predictions with known *S. aureus* prophage-associated genes (*scn*, *sak*, *chp*, *lukS-PV*, *lukF-PV*, *tst*, and *etb*). Prophage types were operationally classified from virulence gene content: φSa3int (IEC-carrying), φSa2 (Panton–Valentine leukocidin [PVL]-carrying), φETA (exfoliative toxin), and SaPI (pathogenicity islands). Insertion sequences (ISs) were screened from Prokka annotations, with particular attention to IS431/IS257 (flanking SCC*mec*) and IS256 (associated with biofilm formation).

### Core genome MLST

A *de novo* whole-genome MLST (wgMLST) schema was generated from the 26 assemblies using chewBBACA v3.5.3 (51), yielding 3,438 loci, of which 546 comprised the strict core genome (present in 100% of isolates) and 1,541 the relaxed core (≥95% presence). Allelic distances were computed, and a minimum spanning tree was constructed.

### Anti-phage defense systems

Bacterial defense systems were identified using DefenseFinder v2.0.1 (52) on Prokka-predicted protein sequences from all 26 assemblies. The DefenseFinder model database was used to classify known anti-phage defense systems, including restriction-modification (RM), CRISPR-Cas, abortive infection (Abi), and recently characterized systems. Differences in defense system prevalence between MRSA and MSSA were assessed using Fisher’s exact test.

### Genotype–phenotype concordance

WGS-based resistance predictions were systematically compared with phenotypic susceptibility results for 10 antimicrobial agents. Genotypic resistance was defined by the presence of established resistance determinants or point mutations: *blaZ* (penicillin), *mecA*/*mecC* (oxacillin), *ermA*/*ermC*/*msrA*/*mphC* (erythromycin), *ermA*/*ermC* (clindamycin), quinolone resistance-determining region (QRDR) mutations *gyrA* S84L/*parC* S80F/S80Y (ciprofloxacin), *tetK*/*tetM* (tetracycline), *dfrG*/*dfrA*/*dfrK* (trimethoprim–sulfamethoxazole), *vanA*/*vanB* (vancomycin), *cfr*/*optrA* (linezolid), and *mprF* mutations (daptomycin). The intrinsic efflux pump *tet(38)* was excluded from tetracycline predictions. Phenotypic susceptibility was classified per CLSI 2024 breakpoints, with intermediate results grouped as non-susceptible. Categorical agreement, sensitivity, specificity, positive predictive value, and negative predictive value were calculated per antibiotic and overall.

### Case-control analysis

A frequency-matched case-control analysis was conducted to identify clinical risk factors for MRSA infection. The 13 MRSA isolates served as cases, and 39 MSSA controls were drawn from routine cultures at the same institution during the overlapping study sampling period (Q1–Q2 2025) at a 1:3 case-to-control ratio (*N* = 52). The 39 controls comprised the 13 MSSA isolates from the WGS cohort (identified by their SA codes throughout this manuscript) plus 26 additional routine hospital MSSA isolates from the institutional Laboratory Information System used only for the epidemiological matching (not whole-genome sequenced); the full de-identified case-control analytic registry is provided in Table S4. Frequency matching was empirically balanced after applying the corrected clinical ID mapping used throughout the revised manuscript: cases and controls did not differ significantly in age (median 46 vs 41 years; mean 49.7 vs 43.1 years; Mann–Whitney *U* test *P* = 0.32; Welch’s *t*-test *P* = 0.35), sex (male 92.3% vs 76.9%; Fisher’s exact test *P* = 0.42), or sampling period (Mann–Whitney *U* test on culture-day index *P* = 0.83; both groups within Q1–Q2 2025). Clinical data were abstracted by a single investigator blinded to genomic results; variables included demographics, healthcare exposure (prior hospitalization within 12 months, current intensive care unit admission, inter-hospital transfer, and long-term care facility residence), comorbidities, invasive devices, surgery within 30 days, prior antibiotic exposure within 90 days, empiric therapy, and outcomes. A composite healthcare exposure score (0–7) was calculated, comprising seven binary indicators: prior hospitalization (12 months), intensive care unit admission, inter-hospital transfer, long-term care facility residence, prior antibiotic exposure (90 days), presence of an invasive device, and surgery within 30 days. Categorical variables were compared using Fisher’s exact test and continuous variables using the Mann–Whitney *U* test. Multivariable analysis employed Firth penalized logistic regression (53, 54) to address separation and small-sample bias; variables with *P* < 0.10 in univariable analysis were entered into the multivariable model. All statistical analyses were performed in R v4.5.2 (R Foundation for Statistical Computing, Vienna, Austria); two-sided *P* values < 0.05 were considered statistically significant.

## RESULTS

### Cohort description and risk factors for MRSA infection

The WGS cohort comprised 26 patients (13 MRSA and 13 MSSA) sampled from the Antiguo Hospital Civil de Guadalajara during 2024–2025. Median age was 42 years (interquartile range, 31–68), and 80.8% (21/26) were male. Specimen sources included blood (10/26), abscess and tissue (8/26), respiratory (5/26), urine (2/26), and biopsy (1/26) (Table S6). Patient demographics, infection type, sampling site, and clinical characteristics for both MRSA and MSSA cases are provided in Table S6; healthcare-associated vs community-associated classification per CDC 2013 narrative criteria (55) is provided in Table S7.

This exploratory matched case-control analysis compared 13 MRSA cases with 39 MSSA controls (Table 2). Prior antibiotic exposure within 90 days was the sole variable significantly associated with MRSA in the univariable analysis: 76.9% of MRSA vs 30.8% of MSSA patients (OR, 7.50; 95% CI, 1.66–26.29; *P* = 0.008). The composite healthcare exposure score was higher in MRSA cases (median 3 vs 2, *P* = 0.045).

**TABLE 2:** Demographic and clinical characteristics of patients with MRSA and MSSA infections (matched case-control analysis, 1:3 ratio)^*a*^

Variable	Total (*N* = 52)	MRSA (*n* = 13)	MSSA (*n* = 39)	OR (95% CI)	*P*
Demographics					
Age (years), median (IQR)	42 (31–68)	46 (41–69)	41 (31–66)	–	0.319
Male sex, *n* (%)	42 (80.8)	12 (92.3)	30 (76.9)	3.60 (0.41–170.60)	0.419
Healthcare exposure					
Previous hospitalization (12 months)	10/51 (19.6)	2/13 (15.4)	8/38 (21.1)	0.68 (0.06–4.26)	1.000
ICU admission at the time of culture	16/50 (32.0)	7/13 (53.8)	9/37 (24.3)	3.63 (0.96–12.47)	0.082
Transfer from another hospital	3/52 (5.8)	0/13 (0.0)	3/39 (7.7)	–	0.564
Long-term care facility	1/52 (1.9)	1/13 (7.7)	0/39 (0.0)	–	0.250
Comorbidities					
Diabetes mellitus	9/52 (17.3)	1/13 (7.7)	8/39 (20.5)	0.32 (0.01–2.93)	0.420
CKD/hemodialysis	9/52 (17.3)	0/13 (0.0)	9/39 (23.1)	–	0.091
HIV infection	3/52 (5.8)	2/13 (15.4)	1/39 (2.6)	6.91 (0.66–46.89)	0.151
Active malignancy	4/52 (7.7)	1/13 (7.7)	3/39 (7.7)	1.00 (0.17–9.41)	1.000
Burn injury	6/52 (11.5)	3/13 (23.1)	3/39 (7.7)	3.60 (0.68–17.79)	0.157
COPD	3/52 (5.8)	1/13 (7.7)	2/39 (5.1)	1.54 (0.02–31.87)	1.000
Heart failure	5/52 (9.6)	3/13 (23.1)	2/39 (5.1)	5.55 (0.86–29.10)	0.093
Invasive devices					
Central venous catheter	18/52 (34.6)	6/13 (46.2)	12/39 (30.8)	1.93 (0.43–8.33)	0.334
Urinary catheter	25/52 (48.1)	9/13 (69.2)	16/39 (41.0)	3.23 (0.73–16.59)	0.112
Mechanical ventilation	13/52 (25.0)	6/13 (46.2)	7/39 (17.9)	3.92 (0.79–18.65)	0.064
Surgery (30 days)	23/52 (44.2)	5/13 (38.5)	18/39 (46.2)	0.73 (0.16–3.10)	0.752
Prior antibiotic exposure (90 days)					
Any antibiotic	22/52 (42.3)	10/13 (76.9)	12/39 (30.8)	7.50 (1.66–26.29)	**0.008**
Composite score, median (IQR)					
Healthcare exposure score (0–7)	2 (1–3)	3 (2–4)	2 (1–3)	–	**0.045**

^*a*^ OR, odds ratio; CI, confidence interval; IQR, interquartile range; ICU, intensive care unit; CKD, chronic kidney disease; COPD, chronic obstructive pulmonary disease. Fisher’s exact test for categorical variables; Mann–Whitney for continuous variables. Bold P values indicate variables significantly associated with MRSA infection (P < 0.05). A dash (‘−’) in the OR column indicates that an odds ratio is not reported (continuous variables compared with the Mann–Whitney U test; or categorical variables with zero events in one group, for which an OR is not finitely estimable).

In Firth penalized logistic regression (Table 3), prior antibiotics and ICU admission were independently significant: prior antibiotics aOR 9.61 (95% CI, 2.24–60.48; *P* = 0.002) and ICU admission aOR 6.17 (95% CI, 1.40–36.89; *P* = 0.015). Each additional point on the healthcare exposure score was associated with 66% increased odds of MRSA (aOR, 1.66; *P* = 0.042).

**TABLE 3:** Exploratory Firth penalized logistic regression for independent risk factors of MRSA infection^*a*^

Model	Variable	aOR (95% CI)	*P*
1 (univariable)	Prior antibiotics (90 days)	6.60 (1.78–30.10)	0.004
2 (multivariable)	Prior antibiotics (90 days)	9.61 (2.24–60.48)	0.002
	ICU admission	6.17 (1.40–36.89)	0.015
3 (composite)	Healthcare score (per point)	1.66 (1.02–2.92)	0.042

^*a*^ Firth’s penalized likelihood method was used to address small-sample bias and quasi-complete separation. Healthcare exposure score: sum of prior hospitalization (12 months), ICU admission, transfer, long-term care, prior antibiotics (90 days), invasive device, and surgery (30 days).

In an exploratory analysis of clinical outcomes (Table S8), adequate empiric therapy was less frequent among MRSA cases than MSSA controls (2/13 [15.4%] vs 34/38 [89.5%], *P* < 0.001). In-hospital mortality was higher among evaluable MRSA cases (5/11 [45.5%] vs 5/39 [12.8%], OR 5.67, *P* = 0.030). The five in-hospital MRSA deaths involved SA001 (hospital-acquired pneumonia), SA015 (hospital-acquired pneumonia), SA019 (infective endocarditis), SA024 (infective endocarditis), and SA026 (catheter-associated urinary tract infection with malignancy)—all belonging to the CC5-SCC*mec* II lineage. Given the small sample size, these results are preliminary and require validation in a larger cohort; clinical outcomes, in particular, should be interpreted as hypothesis-generating.

### Sequencing and assembly

All 26 isolates from the cohort described above yielded assemblies sufficient for species confirmation, molecular typing, and gene detection (Tables S1 and S22). Sequencing produced a median of 456,389 read pairs per sample. Post-trimming read retention exceeded 96% in all isolates, and Q30 scores averaged 96.3%. Mean assembled genome size was 2.80 Mb (range, 2.69–3.46 Mb) with a median *N*_50_ of 168 kb. SA015 (CC5-MRSA) produced a fragmented assembly (1,990 contigs, *N*_50_ = 1.7 kb) attributed to low input DNA quality, precluding reliable plasmid reconstruction but retaining sufficient contiguity for resistance and virulence gene detection. SA016 (ST4552, CC97) yielded an unusually large genome (3.46 Mb), likely attributable to a high burden of mobile genetic elements (prophages and plasmids; Tables S9 and S15); contamination was ruled out by Kraken2 (>99% *S*. *aureus*). All assemblies passed species confirmation by four convergent methods: whole-genome ANI (≥97.28% to NCTC8325, ≥ 95% to at least one *S*. *aureus* reference; Table S2), Kraken2 read classification (≥98% *S*. *aureus*), MLST against the species-specific PubMLST scheme, and *spa* typing.

### Molecular typing and clonal structure

**Fig 1:**
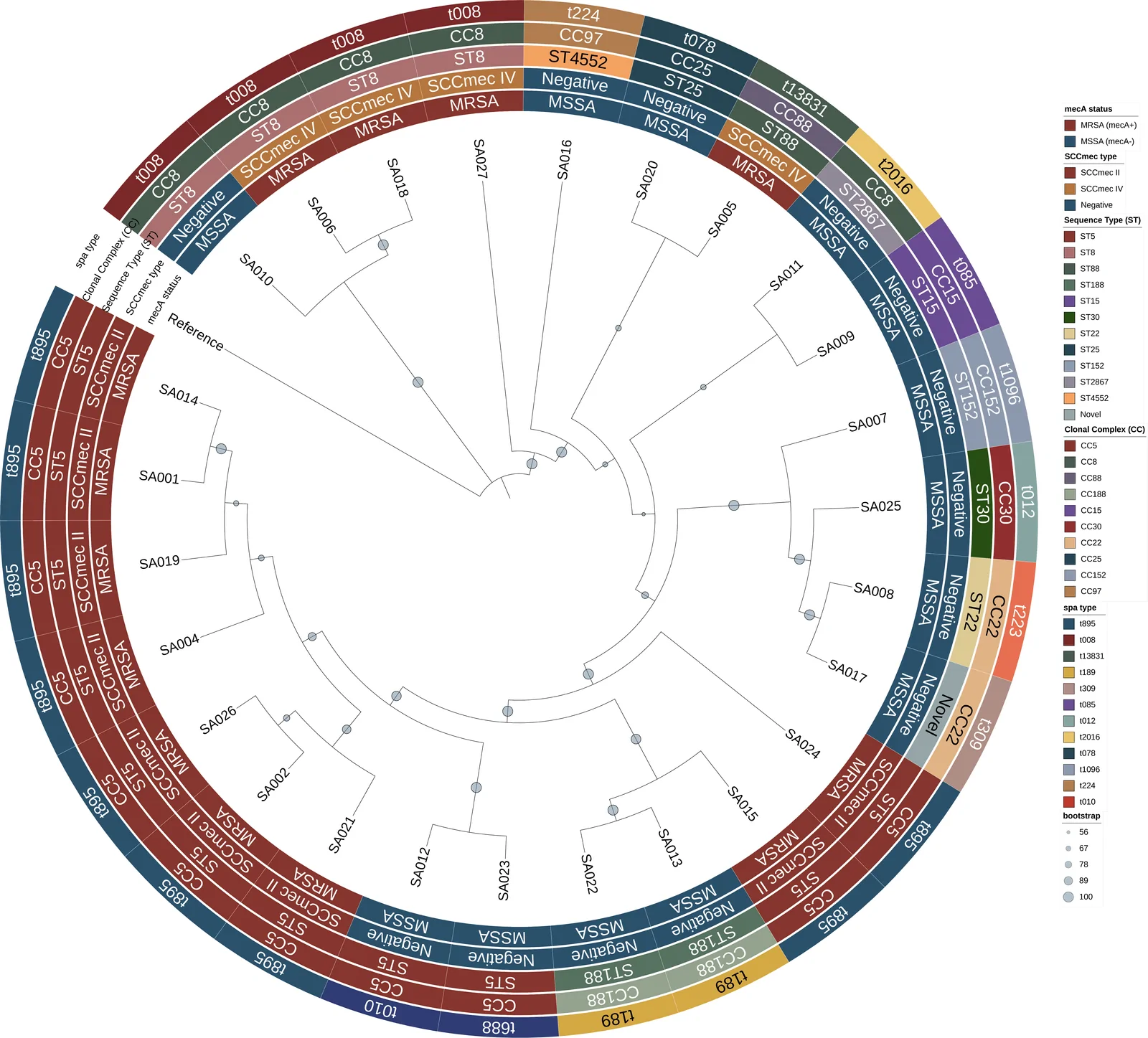
Recombination-filtered maximum-likelihood phylogeny of 26 *S. aureus* clinical isolates with molecular typing annotations. The tree was inferred with IQ-TREE v2.3.6 (model TVM + F + ASC + R3) from a core-SNP alignment after recombination filtering with Gubbins v3.4.3. Branch support values are from 1,000 ultrafast bootstrap replicates. Annotation rings from inner to outer indicate methicillin resistance status (MRSA, red; MSSA, blue), clonal complex, SCC*mec* type, *agr* group, and PVL status. The dominant CC5-SCC*mec* II clade includes nine MRSA isolates and one MSSA isolate (SA023, ST5-t688), the latter providing a single observation consistent with SCC*mec* excision.

**Fig 2:**
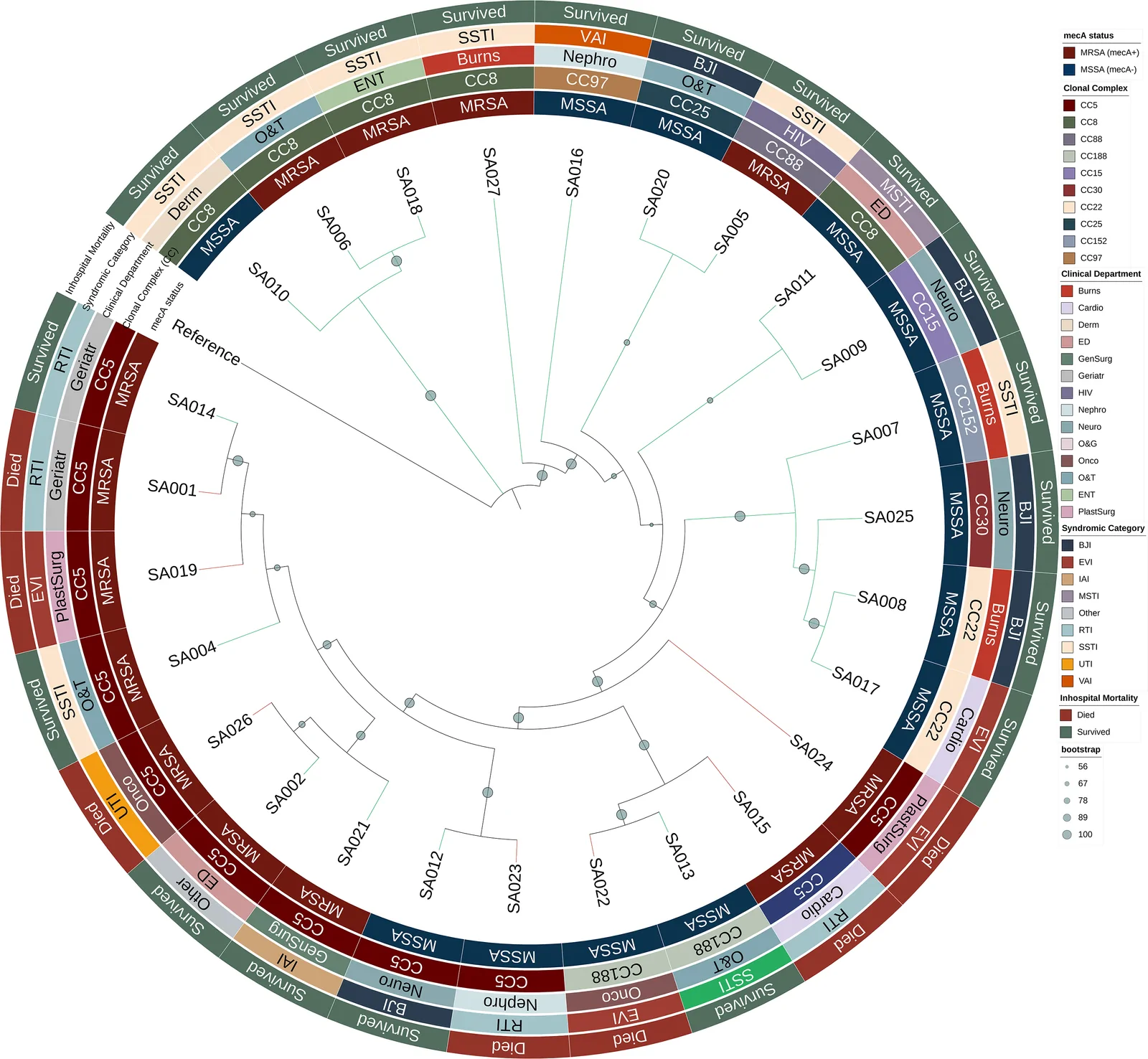
Recombination-filtered phylogeny with integrated clinical annotations. The same tree as Fig. 1 is annotated with methicillin resistance status, clinical syndrome (skin and soft tissue infection [SSTI], bone and joint infection [BJI], endovascular infection [EVI], respiratory tract infection [RTI], intra-abdominal infection [IAI], urinary tract infection [UTI], and vascular access infection [VAI]), hospital department, and in-hospital outcome (alive/deceased). This integrated view illustrates the clinical context of each isolate relative to its phylogenomic position. Deceased patients are concentrated within the CC5-SCC*mec* II clade.

Molecular typing revealed a strikingly asymmetric population structure (Table 1; Table S5; Fig. 1 and 2; Fig. S1). MRSA was dominated by a single clone: ST5-t895-SCC*mec* II (CC5), accounting for 9/13 (69.2%) of MRSA isolates. This lineage corresponds to the New York/Japan pandemic HA-MRSA clone, the predominant strain in Mexican hospitals (18, 19, 56, 57). The remaining MRSA comprised ST8-t008-SCC*mec* IV (CC8; 3/13, 23.1%), a lineage associated with community-onset infections (20, 21), and ST88-t13831-SCC*mec* IV (CC88; 1/13, 7.7%), which is also community-associated (58).

In contrast, MSSA encompassed 11 distinct sequence types across 9 clonal complexes (Table 1), including CC5, CC8, CC15, CC22, CC25, CC30, CC97, CC152, and CC188—with CC97 representing a putative zoonotic-associated lineage (see below). All CC5-MRSA isolates belonged to *agr* group II, consistent with the New York/Japan lineage, while *agr* group I predominated among CC8 and MSSA isolates (Table S5; Fig. S1). Simpson’s diversity index was 1 − D = 0.500 for MRSA vs 1 − D = 0.974 for MSSA; Pielou’s evenness was J′ = 0.719 vs 0.981, confirming marked clonal concentration in MRSA contrasted with a highly diverse MSSA population. SA016 (ST4552, CC97) was the sole isolate with a zoonotic-associated clonal complex; CC97 is predominantly found in livestock and has only sporadically been reported in human infections (59). However, prophage analysis identified a complete φSa3int-associated IEC gene set in SA016 (Table S15), so IEC status cannot be used here as evidence for a non-human reservoir origin.

### Antimicrobial resistance profiles

Resistance gene profiling revealed distinct arsenals in MRSA and MSSA (Table 1; Table S11). The β-lactamase gene *blaZ* was detected in 22/26 (84.6%) isolates. Phenotype–genotype concordance for methicillin resistance was 100% (26/26; Table S10): every cefoxitin-positive isolate carried *mecA*, and every cefoxitin-negative isolate lacked it.

The MLS_B_ resistance gene *ermA* was present in all nine CC5-MRSA-SCC*mec* II isolates (carried on Tn554 within SCC*mec* II), while *ermC* was detected in 5/26 (19.2%) isolates, primarily CC8 and CC88 MRSA. Combined, 14/26 (53.8%) carried at least one *erm* gene. The aminoglycoside resistance gene *aadD* was found in 14/26 (53.8%), predominantly within CC5-MRSA. High-level mupirocin resistance (*mupA*) was detected in 2/26 (7.7%). No vancomycin (*vanA*/*vanB*) or linezolid (*cfr*/*optrA*) resistance genes were detected. Inducible clindamycin resistance, mediated by *ermA* or *ermC*, was detected in 14/26 (53.8%) isolates, underscoring the need for routine D-zone testing prior to clindamycin prescribing.

SA020 (ST25-CC25-MSSA) exhibited a borderline oxacillin-resistant *S. aureus* phenotype: the cefoxitin screen was negative, yet the oxacillin MIC was ≥4 µg/mL. No *mecA* or *mecC* was detected. This isolate carried *blaZ*, which may be consistent with β-lactamase hyperproduction as the mechanism for borderline resistance. Notably, all isolates were susceptible to vancomycin (MIC ≤2 µg/mL), linezolid (MIC ≤4 µg/mL), and daptomycin (MIC ≤1 µg/mL); trimethoprim–sulfamethoxazole non-susceptibility was observed only in SA018 (MIC, 20 μg/mL) without detected acquired *dfr* determinants (Table S11). This preserved susceptibility to vancomycin, linezolid, and daptomycin is clinically reassuring and contrasts with reports of emerging vancomycin-intermediate *S. aureus* from other Mexican institutions.

### Virulence factor profiles

Virulence screening identified 94 unique virulence-associated genes (Table 1; Table S23; Fig. S2; Data S1). Panton-Valentine leukocidin genes (*lukS-PV*/*lukF-PV*) were detected in 6/26 (23.1%) isolates: SA005 (CC88-MRSA, SCC*mec* IV; CA), SA006 (CC8-MRSA, SCC*mec* IV; CA), SA018 (CC8-MRSA, SCC*mec* IV; CA), SA027 (CC8-MRSA, SCC*mec* IV; CA, burn wound), SA007 (CC152-MSSA; CA, burn wound), and SA010 (CC8-MSSA; CA). Thus, PVL positivity comprised four CA-MRSA isolates and two CA-MSSA isolates. PVL was absent from all nine CC5-MRSA-SCC*mec* II isolates, consistent with the typical absence of φSa2 prophage from the New York/Japan clone. Prophage-informed IEC annotation detected at least one φSa3int-associated IEC gene in all 26 isolates, with complete *scn + chp + sak* configurations in 17/26 and partial configurations in 9/26 (Tables S12 and S15). Complete IEC was present in 10/13 MRSA and 7/13 MSSA isolates; SA016 carried complete IEC, whereas SA017 carried partial *sak + scn*. ACME and COMER screening was negative for all 26 isolates, indicating the CC8-SCC*mec* IV isolates do not belong to the classic USA300 or USA300-LV sublineages (21).

### Genomic markers related to SCC*mec* burden and colonization

Literature-linked genomic markers distinguished MRSA and MSSA primarily through SCC*mec* cassette burden (Table 1; Table S12). All nine CC5-SCC*mec* II isolates carried a ~53 kb cassette (predicted higher metabolic burden based on cassette size and gene content; not measured experimentally in this study), while the four CC8/CC88-SCC*mec* IV isolates harbored the smaller ~24 kb element (predicted lower burden). ACME (*arcA*/*opp3*) was absent in all 26 isolates, including the three CC8-SCC*mec* IV MRSA, confirming that these are not USA300 and lack the ACME-associated colonization advantage described for that lineage. Capsule typing revealed uniform CP8 across all isolates, contrasting with CP5 predominance reported in a large U.S. clinical-isolate survey. QRDR mutations (*gyrA* S84L, *parC* S80F/S80Y) were detected in all nine CC5-SCC*mec* II isolates and in SA018 (CC8), consistent with the fluoroquinolone resistance phenotype. Acquired *tetK* was identified in SA004, SA013, and SA022, while chromosomal *tet(38)* and *mepA* were present in all isolates. No *vanA*/*vanB*, *cfr*/*optrA*, or *dfrG* resistance determinants were detected.

### Plasmid repertoire

Plasmid reconstruction by MOB-recon identified predicted plasmid clusters in 25 evaluable assemblies (SA015 was excluded from plasmid-summary statistics because its fragmented assembly precludes reliable reconstruction; median, 4; range, 2–7; Table S9). A total of 100 plasmid clusters were detected, of which 75 (75.0%) were non-mobilizable, 24 (24.0%) were mobilizable, and one (1.0%; SA023) was conjugative. Mean total plasmid content was comparable between MRSA (28.6 kb, excluding SA015) and MSSA (35.0 kb). A rep_cluster_1837-type replicon (~5–10 kb), consistent with pUB110-like elements carrying *aadD*, was identified in five CC5-MRSA isolates (SA004, SA019, SA021, SA024, and SA026). In SA023—the SCC*mec* excision candidate—MOB-recon grouped the 18.4-kb *mupA*-bearing plasmid contig and the 1.4-kb *aadD*-bearing fragment into a predicted conjugative AA083 plasmid cluster, consistent with retention of resistance elements outside the SCC*mec* backbone.

### Phylogenomic analysis

The recombination-filtered phylogeny resolved well-defined clonal clusters (Fig. 1 and 2; Fig. S3; Table S13). Gubbins identified 205,980 total SNPs, of which 65,860 (32.0%) fell within recombination blocks, and 140,120 (68.0%) represented clonal SNPs. The median *r*/*m* ratio was 0.537, confirming predominantly clonal evolution.

Within the CC5-MRSA cluster, pairwise SNP distances were low, with putative transmission pairs at ≤15 SNPs (SA004–SA019, 3 SNPs; SA004–SA002, 4 SNPs; and SA002–SA019, 7 SNPs; Data S2). The broader CC5-MRSA cluster spanned <700 within-clade SNPs, consistent with recent clonal spread.

**Fig 3:**
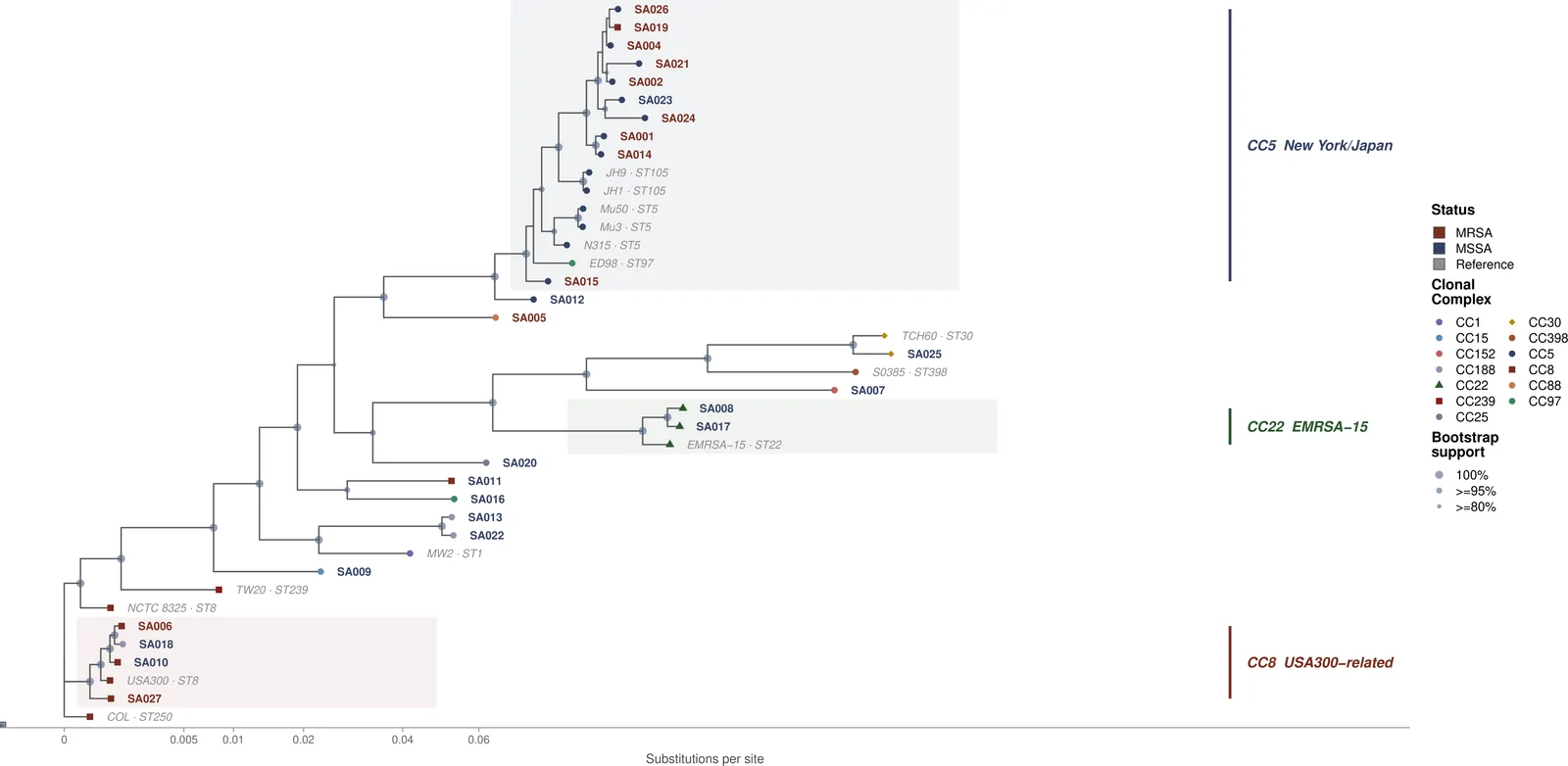
Contextual maximum-likelihood phylogeny including 26 study isolates and 14 globally representative reference genomes (40 taxa; Table S3). The tree was inferred with IQ-TREE v2.3.6 (model GTR + F + R4, 1,000 ultrafast bootstrap replicates). Branch lengths were square-root transformed for visualization to resolve compressed intra-clade structure; axis tick marks show original substitutions per site. Filled circles at internal nodes indicate ultrafast bootstrap support (large, 100%; medium, ≥95%; and small, ≥80%). Tip point shapes denote major clonal complexes (circles, CC5; squares, CC8; and triangles, CC22). Three major clades are highlighted: CC5 New York/Japan (blue shading), CC8 USA300-related (red shading), and CC22 EMRSA-15 (green shading). Local CC5-MRSA-SCC*mec* II isolates cluster with Mu3/N315, confirming their identity as the New York/Japan pandemic lineage. SA023 (ST5-t688-MSSA) nests within the MRSA-CC5 clade, providing a single phylogenomic observation consistent with SCC*mec* excision.

The contextual phylogeny with 14 global reference genomes (40 taxa; Fig. 3; Table S3) placed our CC5-MRSA-SCC*mec* II isolates within the Mu3/N315 subclade, supporting their assignment to the ST5-SCC*mec* II New York/Japan pandemic lineage (56, 57, 60). SA023 (ST5-t688-MSSA), which lacks *mecA* at both assembly and read level, nested within the MRSA-CC5 clade, providing a single phylogenomic observation consistent with SCC*mec* excision (13). The CC8-SCC*mec* IV isolates clustered near USA300-FPR3757, but, lacking ACME and COMER, represent a distinct CC8 sublineage. Between-lineage SNP distances exceeded 6,000 (e.g., CC5 vs CC8: 6,187 SNPs; CC5 vs CC152: 6,167 SNPs), reflecting deep phylogenetic divergence consistent with independent evolutionary histories (Table S13; Fig. S3). SA017 (Novel ST, CC22) and SA008 (ST22, CC22) were separated by only 156 SNPs despite different clinical presentations, compatible with possible nosocomial circulation of a CC22 lineage. Similarly, SA013 and SA022 (both CC188) differed by only 69 SNPs.

Pan-genome analysis identified 6,068 gene clusters, of which 1,168 (19.2%) comprised the core genome, 766 (12.6%) soft-core, 1,174 (19.3%) shell, and 2,960 (48.8%) cloud genes (Table S14). The large accessory genome (80.8%) reflects the substantial genetic diversity captured in this collection, despite the small sample size, largely reflecting the polyclonal MSSA population.

### Prophage content and insertion sequence repertoire

**Fig 4:**
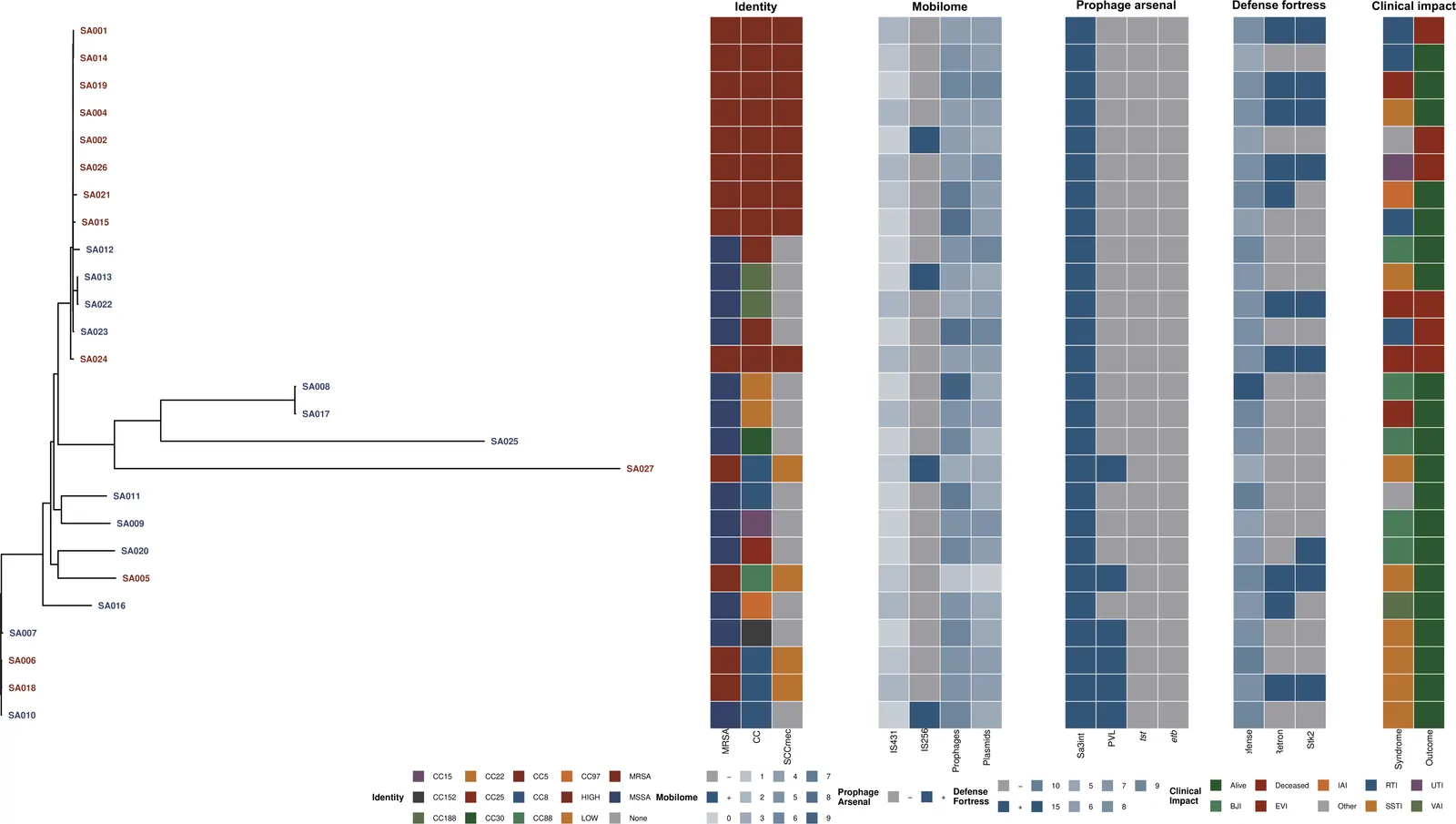
Mobilome landscape of 26 *S. aureus* clinical isolates. The recombination-filtered phylogeny (left) is aligned with five annotation blocks displayed as heatmaps: (i) identity—methicillin resistance status, clonal complex, and SCC*mec* type; (ii) mobilome—IS431 and IS256 presence, prophage count, and plasmid count; (iii) prophage arsenal—Sa3int (φSa3 integrase), PVL (*lukS/F-PV*), *tst* (toxic shock syndrome toxin), and *etb* (exfoliative toxin B); (iv) defense fortress—total defense system count, Retron_III, and Stk2 presence; (v) clinical impact—syndromic category and in-hospital outcome. Color intensity scales are shown in the legend. Gene names *tst* and *etb* are italicized as they represent individual genes. This integrated view reveals the co-occurrence of mobile genetic elements, defense systems, and clinical phenotypes across the phylogeny.

PhiSpy identified 136 prophage regions across the 26 genomes (mean, 5.2 per isolate; range, 1–9; Table S15; Fig. 4). Total prophage content averaged 60.5 kb per genome (range, 7.4–98.3 kb). The φSa3int prophage carrying a complete IEC locus (*scn + chp + sak*) was detected in 17/26 isolates; partial IEC configurations were found in 9 additional isolates. Two isolates—SA007 (CC152) and SA011 (CC8)—harbored *tst* (toxic shock syndrome toxin), indicating the presence of SaPI-type pathogenicity islands. Three isolates (SA015, SA025, and SA027) carried *etb* (exfoliative toxin B), consistent with φETA-type prophages. PVL genes (*lukS-PV*/*lukF-PV*), typically carried by φSa2, were not co-localized within identified prophage regions, likely due to fragmentation across multiple contigs in the short-read assemblies.

Insertion sequence analysis identified a mean of 6.8 IS elements per genome (range, 4–15; Table S16). IS431/IS257 (IS6 family), which commonly flanks SCC*mec* and can participate in cassette mobilization, was significantly more prevalent in MRSA (10/13, 76.9%) than MSSA (3/13, 23.1%). At the chromosomal SCC*mec* integration locus on SA023’s contig 1 (*rlmH*/*orfX*, position 277,749–278,228), no IS431/IS257-family elements were detected within ±50 kb of the integration site, and the immediate flanking genes (*walK*/*walR*/*yycJ* upstream; *glpE*/*gloB*/*dus* downstream) preserved the canonical MSSA chromosomal architecture identical to NCTC8325. Targeted BLAST confirmed an intact *orfX* gene (1,242/1,242 bp, 99.8% identity to NCTC8325) and a native *attB* site 100% identical to the MSSA reference; the wider *orfX* ±10 kb context aligned at 98.4% over 12,633 bp without insertions. Genome-wide, a single IS257-1 transposase was detected on a separate 18,417-bp plasmid contig (SA023_contig_23, GC 29.3%) that also carries *mupA* (high-level mupirocin resistance) and is unrelated to the chromosomal SCC*mec* integration site. A direct gene-by-gene comparison of the canonical SCC*mec* core genes across SA023 and the nine sibling CC5-MRSA isolates (*mecA*, *mecR1*, *mecI*, *ccrA*, and *ccrB*; Table S17) confirmed that all nine sibling MRSA carry the complete *mec*-complex, while SA023 carries zero of these genes. Quantitative read-mapping of SA023’s reads against the N315 SCC*mec* II reference (D86934.1) yielded 94.8% zero coverage across the cassette-specific 36-kb region (*ccrA–mecA* core); residual coverage was restricted to chromosomal flanks, the *kdp* auxiliary operon, and the IS431m elements that cross-map from the SA023 plasmid IS257-1 (Fig. S4). The clean chromosomal *attB*, absence of IS431/IS257 elements at the *orfX* flanking sequences, and focused dotplot of SA023’s chromosomal contig vs the sister CC5-MRSA isolate SA015 (Fig. S5) are consistent with precise *ccr*AB-mediated cassette excision rather than IS431-mediated homologous recombination, although long-read sequencing would be required to resolve the junction definitively. The 18.4-kb *mupA*-bearing plasmid (SA023_contig_23) and a 1.4-kb *aadD*-bearing fragment (SA023_contig_29, consistent with a partial pUB110 derivative) persist at 2.81× and 2.37× chromosomal read depth, respectively, indicating retention as two to three copies per cell. IS256, associated with biofilm formation and vancomycin resistance, was detected in only 4/26 isolates with no MRSA/MSSA enrichment.

### Core genome MLST

The *de novo* wgMLST schema generated by chewBBACA comprised 3,438 loci, of which 546 formed the strict core genome (Table S18). The pan-genome architecture (core, 546; soft-core, 995; and shell, 1,897) was concordant with the Roary-based analysis. Within the CC5-MRSA-SCC*mec* II cluster, pairwise cgMLST distances ranged from 0 to 20 allelic differences, consistent with recent clonal spread. The minimum spanning tree resolved the same major clonal groups identified by SNP phylogeny (Fig. S6). Correlation between cgMLST allelic distances and SNP distances was significant but moderate (Spearman *ρ* = 0.327, *P* < 0.001), reflecting the inherent difference between gene-level (allelic) and nucleotide-level resolution. Notably, the strict core of 546 loci—derived *de novo* from this small collection—provided limited inter-lineage discrimination; a validated reference schema (e.g., cgMLST.org, 1,861 loci) would improve resolution for cross-institutional comparisons.

### Anti-phage defense systems

DefenseFinder identified 209 defense systems across the 26 isolates (mean, 8.0 per genome; range, 5–15; Table S19; Fig. 4). Three systems formed a conserved core: gcu233 (26/26, 100%), Abi2 (25/26, 96%), and Type I restriction-modification (22/26, 85%). No CRISPR-Cas systems were detected, consistent with the known rarity of CRISPR in clinical *S. aureus*. The CC5-SCC*mec* II lineage displayed a conserved defense profile comprising Abi2, FS_Sma, gcu233, Retron_III, RM_Type_I, RosmerTA, and Stk2. Notably, Retron_III was enriched in MRSA (8/13, 62%) compared with MSSA (2/13, 15%), as was Stk2 (7/13, 54% vs 2/13, 15%). Three defense systems—Pycsar, ShosTA, and PD-Lambda-1—were detected exclusively in MSSA isolates. SA008 (CC22-MSSA) was an outlier harboring 15 defense systems, including five RM systems, suggesting an unusually fortified genome. The combined catalog includes 188 entries spanning antimicrobial-resistance determinants, virulence-associated genes, stress/resistance markers, and anti-phage defense systems; DefenseFinder identified 23 defense-system subtypes across the cohort (Tables S19 and S20).

### Genotype–phenotype concordance for antimicrobial resistance

Systematic comparison of WGS-based resistance predictions with phenotypic susceptibility testing across 10 antimicrobial agents yielded an overall categorical agreement of 91.7% (233/254 evaluable isolate–antibiotic combinations; Table S21). Perfect concordance (100%) was observed for vancomycin, linezolid, and daptomycin, where no resistance determinants were detected genomically, and all isolates were phenotypically susceptible. Clindamycin and trimethoprim–sulfamethoxazole achieved ≥96% agreement. Ciprofloxacin showed the lowest concordance (71.4%), attributable to isolates harboring *parC* mutations alone without *gyrA* S84L, which were insufficient to confer phenotypic resistance. Notable discordances included the BORSA isolate SA020 (oxacillin false-negative: phenotypically resistant but lacking *mecA*, consistent with β-lactamase hyperproduction) and two isolates with *mecA*-positive heteroresistance (SA015, SA027), which were classified as oxacillin false-positives because their MICs were below the breakpoint despite positive cefoxitin screens; clinically, these are treated as MRSA. Three tetracycline false-positives (SA002, SA005, and SA017) harbored *tetK* but were phenotypically susceptible, possibly reflecting low gene copy number or non-functional variants. Erythromycin false-negatives (SA013 and SA020) showed phenotypic resistance (MIC ≥8 µg/mL) without detected *erm* or *msr* genes, suggesting non-canonical resistance mechanisms such as promoter mutations or regulatory variants not captured by current databases. Trimethoprim–sulfamethoxazole concordance was 96.2%: SA018 showed phenotypic non-susceptibility (MIC, 20 μg/mL) without detected acquired *dfr* determinants. Table S21 summarizes per-antibiotic concordance metrics and selected clinically relevant discordances.

## DISCUSSION

This study provides the first WGS-based molecular characterization of the *S. aureus* population at a Mexican university referral hospital experiencing a sustained decline in MRSA prevalence. Epidemiological surveillance at this institution has documented a 9.5-year reduction from 28.1% to 14.0% (22), against a broader background of changing MRSA epidemiology in hospital and surveillance settings (1, 16). The genomic data reveal a contemporary low-prevalence population characterized by marked MRSA clonal concentration in a pandemic clone carrying a cassette associated in prior literature with a higher metabolic burden, a single phylogenomic observation consistent with SCC*mec* excision in CC5, and genetically diverse MSSA carrying substantial virulence determinants.

The dominance of ST5-t895-SCC*mec* II among MRSA (69.2%) is concordant with this lineage’s established predominance in Mexican hospitals (18, 19). Contextual phylogenomics placed these isolates within the Mu3/N315 subclade, confirming their identity as the New York/Japan pandemic lineage (56, 57, 60). This marked clonal concentration—only three sequence types among 13 MRSA isolates—contrasts sharply with the broad polyclonal architecture of the MSSA population and resembles prior reports in which highly clonal MRSA populations underwent ecological displacement or decline (9, 61). By analogy, this pattern raises the hypothesis that, when a dominant MRSA clone contracts under changing selection pressures, no secondary MRSA clone necessarily compensates, and susceptible lineages may occupy available ecological space; this remains an inference rather than a demonstrated process in our cohort (9, 10, 61). The low SNP and cgMLST distances among several CC5-SCC*mec* II isolates are compatible with recent clonal dissemination and possible nosocomial circulation of an endemic MRSA lineage. However, this study did not include environmental sampling, healthcare-worker carriage screening, or device-tracing data; therefore, hospital contamination or transmission pathways cannot be formally inferred. The near-twofold difference in Simpson’s diversity (MRSA 0.500 vs MSSA 0.974) quantifies this contrast between a clonally concentrated MRSA population and a diverse MSSA polyclonal structure.

A possible mechanistic context involves the inferred metabolic burden of SCC*mec* II, supported by prior experimental literature on SCC*mec*-associated fitness effects and *psm-mec*-mediated regulation (11, 62) but not measured in this study. The *psm-mec* regulatory RNA within this element has been shown elsewhere to directly suppress *agr* translation, creating an intrinsic resistance-virulence trade-off (62). When antibiotic selection pressure decreases, these experimentally described SCC*mec*-associated fitness costs could plausibly reduce relative competitive fitness (11). Regional Mexican molecular/susceptibility surveillance and the previously described institutional antimicrobial-stewardship program provide only indirect epidemiologic context for a changing MRSA ecology (19, 22); however, antibiotic use and antibiotic selection pressure were not measured longitudinally in the present genomic cohort, so this link remains inferential rather than demonstrated. The SCC*mec* II cassette additionally carries Tn554 (*ermA*, conferring macrolide-lincosamide-streptogramin B resistance) and pUB110 (*aadD*, aminoglycoside resistance), implying a greater metabolic load than the smaller type IV cassette carried by the CC8 lineage—an inference based on cassette size and gene content rather than direct fitness measurement here. This inferred differential burden co-occurs with the distinct resistance profiles: all CC5-MRSA isolates carried both *ermA* and *aadD*, whereas the CC8-MRSA isolates carried *ermC* instead—an independently acquired macrolide resistance gene on a smaller mobile element.

SA023 (ST5-t688-MSSA), which lacks *mecA* at both assembly and read level yet nests within the MRSA-CC5 clade (94–130 SNPs from the nearest CC5-MRSA isolates), constitutes a single isolated observation compatible with SCC*mec* excision as a possible mechanism for MRSA-to-MSSA conversion, paralleling documented events in the ST36 lineage in the United Kingdom (13). Four lines of evidence support this interpretation as a working hypothesis rather than a population-level conclusion: (i) phylogenomic nesting within the MRSA-CC5 clade despite complete absence of *mecA*; (ii) an intact *orfX* gene with a chromosomal *attB* site 100% identical to the native MSSA reference (NCTC8325) and the canonical MSSA flanking architecture (*walK*/*walR*/*yycJ* upstream; *glpE*/*gloB*/*dus* downstream), with no IS431/IS257 elements within ± 50 kb of *orfX*, no *ccr* recombinase fragments, and no residual SCC*mec* sequences at the integration site—consistent with precise *ccr*AB-mediated excision (14); (iii) quantitative read mapping of SA023 against the N315 SCC*mec* II reference confirmed 94.8% zero coverage across the cassette-specific *ccrA–mecA* core (Fig. S4), with the residual signal restricted to conserved chromosomal flanks and IS431m elements that cross-map from a separate SA023 IS257-1 plasmid; and (iv) the focused chromosomal dotplot SA023 vs the sister CC5-MRSA SA015 (Fig. S5) shows an unbroken diagonal across the integration locus, consistent with the absence of insertion at *orfX/attB*. Independent retention of resistance determinants on mobile elements—an 18.4-kb *mupA*-bearing plasmid (SA023_contig_23, 2.81× chromosomal coverage; carrying *mupA*, IS257-1, *ssb*, and *topB*) and a 1.4-kb *aadD*-bearing fragment (SA023_contig_29, 2.37× coverage; consistent with a partial pUB110 derivative)—indicates that, if SCC*mec* loss occurred, associated resistance elements persisted as autonomous replicons. The clean *attB* restoration without IS431 scars is more consistent with the canonical *ccr*AB recombinase-mediated excision pathway (14) than with the IS431-mediated homologous recombination mechanism described in other lineages (63). This potential dissociation, if confirmed, would have implications for the trajectory of antimicrobial resistance after SCC*mec* loss: even after reversion to methicillin susceptibility, clinically relevant resistance determinants (*aadD* and *mupA*) may persist on mobile elements. Because this remains a single-isolate observation, generalization to the wider MRSA decline at this institution requires confirmation in larger longitudinal collections and ideally long-read sequencing to resolve the excision boundaries and the plasmid topology of the released elements.

The CC8-SCC*mec* IV isolates, all PVL-positive, represent a secondary CA-MRSA lineage that lacks ACME and COMER, distinguishing them from USA300 and USA300-LV (20, 21). The absence of these elements, which are characteristic of successful USA300-related backgrounds, may help contextualize the limited representation of CC8 in this small sample rather than the dominance observed for USA300 in other settings. ACME has been proposed to contribute to colonization through the arginine deiminase system; therefore, its absence suggests that these CC8 strains may differ biologically from ACME-positive USA300 lineages. The uniform CP8 capsule across all 26 isolates—including all MRSA—contrasts with a large U.S. clinical-isolate survey in which ~96% of MRSA carried CP5 (64), suggesting a distinct capsular ecology at this institution warranting further investigation.

SA016 (CC97) belongs to a clonal complex commonly associated with bovine and other livestock reservoirs, and sporadic human infections with CC97 have been linked to livestock exposure in prior studies (59). In this isolate, however, prophage analysis detected a complete φSa3int-associated IEC gene set, indicating human-adaptation markers rather than IEC loss. Because no direct epidemiological link to animal exposure was documented, SA016 should be interpreted as a human clinical isolate from a zoonotic-associated lineage, not as a confirmed zoonotic spillover.

The case-control analysis provides a clinical context for the genomic findings. Prior antibiotic exposure was the strongest independent risk factor (aOR, 9.61), consistent with prevention guidance that treats antimicrobial stewardship as a core component of MRSA control (7, 8). The composite healthcare exposure score—integrating hospitalization history, ICU admission, invasive devices, and antibiotic use—was higher in MRSA cases (median 3 vs 2, *P* = 0.045), with each additional point increasing MRSA odds by 66% (aOR, 1.66). This association supports the clinical plausibility of healthcare-associated selection for MRSA but does not, by itself, establish a genomic mechanism for the institutional decline.

The markedly lower adequacy of empiric therapy in MRSA cases (15.4% vs 89.5% in MSSA) highlights a clinical concern in lower-prevalence settings: if empiric anti-MRSA coverage is de-escalated, patients with true MRSA infections may experience worse outcomes. The elevated in-hospital mortality among MRSA cases (45.5% vs 12.8%, *P* = 0.030), while limited by sample size, underscores the persistent lethality of these infections and the clinical imperative for rapid identification. All five MRSA deaths belonged to the CC5-SCC*mec* II lineage, consistent with the association between healthcare-associated MRSA and severe outcomes in immunocompromised hosts. Rapid diagnostic workflows—including PCR-based *mecA* detection for methicillin resistance (65), paired with rapid organism identification by matrix-assisted laser desorption/ionization time-of-flight mass spectrometry (66)—could shorten the time to targeted therapy when coupled to susceptibility or resistance gene testing.

The high prevalence of inducible MLS_B_ resistance (53.8%) argues for mandatory D-testing before clindamycin prescribing, a recommendation with direct implications for empiric treatment protocols in settings where clindamycin remains a first-line option for *S. aureus* skin and soft tissue infections. The identification of *mupA* in both MRSA (SA018) and MSSA (SA023) backgrounds raises concerns about mupirocin-based decolonization sustainability, particularly given that SA023—the SCC*mec* excision candidate—carries mupirocin resistance, suggesting that this determinant persists independently of the resistance cassette. The universal absence of vancomycin and linezolid resistance is reassuring for current treatment strategies but requires ongoing genomic surveillance. The genotype–phenotype concordance analysis supported the clinical utility of WGS-based resistance prediction, with 91.7% overall agreement and 100% concordance for last-resort agents (vancomycin, linezolid, and daptomycin). The lower concordance for ciprofloxacin (71.4%) reflects the complexity of fluoroquinolone resistance: isolates harboring *parC* S80F/S80Y mutations without a concomitant *gyrA* S84L substitution were genotypically resistant but phenotypically susceptible, consistent with the established requirement for dual-target QRDR mutations to achieve clinically relevant fluoroquinolone resistance in *S. aureus* (67). Similarly, three tetracycline false-positives (SA002, SA005, and SA017) harbored *tetK* without phenotypic resistance, possibly reflecting low gene copy number, promoter variants, or non-functional alleles that fail to produce sufficient efflux pump activity. These discordances highlight that gene detection without contextual interpretation can overpredict resistance. The high concordance for trimethoprim–sulfamethoxazole (96.2%) and complete concordance for vancomycin, linezolid, and daptomycin support WGS-based screening for these critical agents and the potential implementation of genomic surveillance in resource-limited settings.

MDR-MSSA was also observed in clinically relevant forms. SA013 showed phenotypic non-susceptibility to penicillin, erythromycin, and tetracycline together with multiple resistance determinants, whereas SA017 carried a novel CC22 background with genotypic determinants that overpredicted tetracycline resistance. SA020 represented a BORSA phenotype: cefoxitin-negative and *mecA*/*mecC*-negative, but oxacillin-resistant with *blaZ*-associated β-lactamase hyperproduction. These MSSA findings reinforce that methicillin susceptibility does not exclude multidrug-resistant phenotypes or clinically relevant β-lactam non-susceptibility, supporting careful resistance phenotyping and, where available, genotypic follow-up in both MRSA and MSSA; Latin American surveillance studies underscore the clinical burden and regional heterogeneity of *S. aureus* resistance (16, 17).

The prophage and insertion sequence analyses provide a mechanistic context for the SCC*mec* excision hypothesis in SA023. The absence of IS431 remnants at the *attB* site and the clean restoration of the native chromosomal junction are more consistent with the canonical *ccr*AB recombinase pathway (14) than with the IS431-mediated homologous recombination model described in other lineages (63, 68); nevertheless, short-read data cannot definitively resolve the excision boundaries. The unexpected detection of *tst* in two MSSA isolates (SA007 and SA011) and *etb* in three isolates (SA015, SA025, and SA027) reveals a broader toxin landscape than captured by the initial VFDB screening, as these SaPI-encoded (69) and phage-encoded toxins require prophage-specific detection approaches. The high prevalence of φSa3int (IEC-carrying) prophages across both MRSA and MSSA confirms that immune evasion is a common feature of human-adapted *S. aureus* (43).

The defense system analysis revealed an additional genomic difference between MRSA and MSSA isolates. The enrichment of Retron_III and Stk2 in MRSA isolates—particularly within the CC5-SCC*mec* II lineage—suggests that defense system acquisition may co-occur with SCC*mec* carriage, consistent with recent evidence that anti-phage defenses are co-mobilized alongside SCC*mec* on SCC-like elements (70). If such co-mobilization applies to local CC5 isolates, SCC*mec* excision could also remove linked anti-phage defenses; this remains a hypothesis requiring long-read structural resolution and functional validation. The complete absence of CRISPR-Cas is notable, but its functional consequences cannot be inferred from these data alone. The exclusive presence of Pycsar, ShosTA, and PD-Lambda-1 in MSSA suggests lineage-specific defense repertoires that may influence phage susceptibility and, consequently, virulence gene acquisition via transduction.

These findings carry broader implications for understanding MRSA epidemiology in Mexico and Latin America. WGS-based molecular characterization of clinical *S. aureus* remains comparatively limited in Mexico, although recent multicenter Mexican genomic surveillance has begun to address this gap and supports the prominence of ST5-t895-SCC*mec* II in hospitals (19). The clonal homogeneity documented here—with a single pandemic lineage accounting for nearly 70% of MRSA—is therefore concordant with Mexican multicenter genomic data rather than evidence by itself of a broader regional pattern. However, the co-circulation of CA-MRSA CC8 lineages lacking ACME and COMER distinguishes the Mexican epidemiology from that of the United States, where USA300 (ACME-positive) has achieved dominance in both community and healthcare settings (71). The plasmid repertoire analysis further revealed that most predicted plasmid clusters were small and non-mobilizable (75.0%), suggesting limited horizontal transfer potential and a pattern dominated by clonal expansion rather than lateral gene exchange.

Several limitations should be acknowledged. We did not perform *in vitro* growth or competition assays; references to fitness effects or metabolic burden rely on prior experimental literature for SCC*mec*-associated fitness effects and *psm-mec*-mediated regulation (11, 62), and direct fitness measurements at this institution would be needed to confirm that the published burden estimates apply to local lineages. Likewise, allele-level variation within shared genes and chromosomal background effects also influence fitness and were not modeled here. The sample of 26 isolates provides a high-resolution genomic snapshot of the dominant circulating lineages, rather than an exhaustive population survey. Critically, the study objective was the characterization of the clonal architecture during prevalence decline, not the estimation of prevalence itself. The marked clonal concentration of the MRSA population—with pairwise cgMLST distances of 0–20 allelic differences among CC5-SCC*mec* II isolates (Fig. S6)—suggests that additional contemporary MRSA sampling from this institution may add limited lineage-level diversity, although larger sampling would still be required for formal population inference. While sufficient to characterize the clonal structure and identify major resistance and virulence determinants through short-read WGS at adequate depth (Table S1), the sample offers limited statistical power for subgroup comparisons. The case-control analysis with 13 MRSA events is underpowered to detect associations with OR < 3.0, and results should be interpreted as hypothesis-generating; nonetheless, the strong effect size for prior antibiotic exposure (aOR, 9.61) was robust to the small sample. Cases and controls were frequency-matched at the cohort level after applying the corrected clinical ID mapping and showed no statistically significant differences in age, sex, or sampling period (Materials and Methods); however, an unconditional logistic-regression model (Firth penalized) was used rather than a conditional pair-matched model because individual triplet matching was not enforced. Isolates represent a single time point during the low-prevalence period; without WGS data from the high-prevalence era (~2016), evidence for clonal replacement is indirect but supported by the marked clonal concentration observed. Short-read sequencing may not fully resolve SCC*mec* structural variants, plasmid topology, or the precise boundaries of the excision event in SA023; long-read sequencing would enable definitive resolution (72). As a single-center study, findings may not generalize to other Mexican regions; however, the dominance of ST5-t895-SCC*mec* II is consistent with multicenter surveillance data from Mexican hospitals (19), suggesting that the clonal concentration in MRSA documented here may extend beyond this institution.

### Conclusions

This study provides a WGS characterization of *S. aureus* at a Mexican university referral hospital during the low-prevalence phase after a documented decade-long MRSA decline. The contemporary MRSA sample was highly clonal, dominated by ST5-t895-SCC*mec* II (New York/Japan), a pandemic lineage carrying a cassette associated in prior literature with higher metabolic burden, whereas MSSA was genetically diverse. One CC5-MSSA isolate nested within the MRSA-CC5 clade and lacked SCC*mec*; together with the absence of ACME and COMER in secondary CC8 MRSA, these observations define genomic patterns that warrant longitudinal follow-up but do not establish a mechanism for the decline. A larger genomic surveillance study spanning high- and low-prevalence eras, ideally with long-read sequencing, is required to test these hypotheses formally. The secondary case-control analysis identified prior antibiotic exposure and ICU admission as clinical correlates of MRSA, and lower empiric-therapy adequacy in MRSA cases underscores the persistent clinical impact of resistant infections even during declining prevalence. Sustained genomic surveillance is essential to track clonal dynamics and guide evidence-based interventions.

## Supplementary Material

Reviewer comments

## Data Availability

Raw sequencing data have been deposited in the NCBI Sequence Read Archive under BioProject PRJNA1437481. Assembled genomes are available in GenBank under the same BioProject (individual accession numbers are listed in Tables S1 and S22). Supplementary data files (virulence matrix, SNP distance matrix, core-genome alignment, phylogenetic trees, and iTOL annotation files) are available at Zenodo (https://doi.org/10.5281/zenodo.19026167).

## SUPPLEMENTAL MATERIAL

Supplemental material spectrum.00948-26-s0001.pdf Tables S1 to S23; Fig. S1 to S6; descriptions for Data S1 to S5, hosted on Zenodo.

OPEN PEER REVIEW reviewer-comments.pdf An accounting of the reviewer comments and feedback.

ASM does not own the copyrights to Supplemental Material that may be linked to, or accessed through, an article. The authors have granted ASM a non-exclusive, world-wide license to publish the Supplemental Material files. Please contact the corresponding author directly for reuse.
