# Supplementary material for: Whole-genome sequencing of Staphylococcus aureus isolates after a decade-long decline in MRSA prevalence at a university referral hospital in Guadalajara, Mexico: clonal concentration in MRSA and greater genetic diversity in MSSA
Source: Microbiol Spectr. 2026 Jun 15;14(7):e00948-26. doi: 10.1128/spectrum.00948-26 (PMC13340018; doi:10.1128/spectrum.00948-26)
Supplement: Supplemental material — Tables S1 to S23; Fig. S1 to S6; descriptions for Data S1 to S5, hosted on Zenodo. [file spectrum.00948-26-s0001.pdf]

AMERICAN SOCIETY FOR MICROBIOLOGY

Journals.ASM.org

Supplementary Materials

Whole-Genome Sequencing of *Staphylococcus aureus* Isolates
after a Decade-Long Decline in MRSA Prevalence
at a University Referral Hospital in Guadalajara, Mexico:
Clonal Concentration in MRSA and Greater Genetic Diversity in MSSA

Contents

Table S1. Sequencing Quality Control Metrics 2
Table S2. Whole-genome ANI Species ID 3
Table S3. Reference Genomes for Contextual Phylogeny 4
Table S4. Case-Control Analytic Registry 5
Table S5. MLST Allelic Profiles 9
Table S6. Patient Demographics 10
Table S7. HA/CA Classification 11
Table S8. Clinical Outcomes (Exploratory) 12
Table S9. Plasmid Repertoire 13
Table S10. Phenotype–Genotype Concordance 14
Table S11. Resistance Gene Profiles 15
Table S12. SCCmec Burden and Colonization Markers 16
Table S13. SNP Distance Matrix 18
Table S14. Pan-genome Composition 19
Table S15. Prophage Content 20
Table S16. Insertion Sequence Elements 21
Table S17. Mec Complex Comparison 22
Table S18. Core Genome MLST 23
Table S19. Anti-Phage Defense Systems 24
Table S20. Gene/System Catalogue 25
Table S21. Genotype–Phenotype Concordance 32
Table S22. Assembly Statistics 33
Table S23. Virulence Factor Inventory 34
Figure S1. Population structure and genomic markers (MRSA vs MSSA) 35
Figure S2. Combined antimicrobial-resistance and virulence heatmap 36
Figure S3. Pairwise SNP distance heatmap 37
Figure S4. SCCmec II read-coverage track (SA023) 38
Figure S5. Focused dotplot SA023 vs SA015 39
Figure S6. cgMLST minimum spanning tree 40
Supplementary Data Files 41

Table S1. Sequencing quality control metrics for 26 *S. aureus* isolates

Sequencing was performed on the Illumina MiSeq platform in paired-end mode (2×200 bp). Reads were processed with Fastp (adapter removal, quality trimming Q≥20, minimum length 50 bp). Coverage was estimated assuming a 2.8 Mb reference genome. One pre-replacement sequencing attempt (460 total reads) failed QC and was excluded prior to analysis, leaving 26 analyzable isolates.

Sample	BioSample	SRA	Raw Reads	Trimmed	Ret. (%)	Q30 (%)	GC (%)	Adap. (%)	Dup. (%)	Cov. (×)	QC	Notes
SA001	SAMN56511660	SRR37630441	639,506	628,842	98.3	95.6	32.9	0.40	0.37	40.1	PASS	
SA002	SAMN56511661	SRR37630440	1,056,030	1,042,914	98.8	96.3	33.2	0.56	0.66	63.2	PASS	
SA004	SAMN56511662	SRR37630429	2,116,910	2,099,776	99.2	96.3	33.1	0.32	1.14	133.4	PASS	
SA005	SAMN56511663	SRR37630422	1,000,282	988,986	98.9	96.6	32.9	0.37	0.59	62.2	PASS	
SA006	SAMN56511664	SRR37630421	654,394	647,872	99.0	96.1	32.8	0.26	0.41	41.0	PASS	
SA007	SAMN56511665	SRR37630420	969,324	948,214	97.8	95.7	32.9	0.22	0.69	54.2	PASS	
SA008	SAMN56511666	SRR37630419	985,520	972,802	98.7	96.3	32.9	0.32	0.60	60.7	PASS	
SA009	SAMN56511667	SRR37630418	1,281,490	1,266,250	98.8	95.8	32.8	0.43	0.74	81.6	PASS	
SA010	SAMN56511668	SRR37630417	1,538,758	1,525,216	99.1	96.5	32.9	0.37	0.88	96.4	PASS	
SA011	SAMN56511669	SRR37630416	1,002,844	990,248	98.7	96.8	33.0	0.39	0.58	64.2	PASS	
SA012	SAMN56511670	SRR37630439	747,496	740,944	99.1	96.3	33.0	0.39	0.44	46.5	PASS	
SA013	SAMN56511671	SRR37630438	1,144,888	1,135,574	99.2	94.7	32.9	0.45	0.53	68.5	PASS	
SA014	SAMN56511672	SRR37630437	1,170,778	1,161,276	99.2	96.3	32.9	0.33	0.57	72.4	PASS	
SA015	SAMN56511673	SRR37630436	521,396	506,866	97.2	96.6	33.1	0.48	0.56	29.2	WARN	Low cov.
SA016	SAMN56511674	SRR37630435	679,048	671,162	98.8	96.7	32.9	0.30	0.40	43.7	PASS	
SA017	SAMN56511675	SRR37630434	1,094,642	1,065,688	97.4	96.4	33.0	0.48	0.93	61.2	PASS	
SA018	SAMN56511676	SRR37630433	2,202,398	2,143,436	97.3	96.7	32.9	0.65	1.39	122.6	PASS	
SA019	SAMN56511677	SRR37630432	914,610	891,042	97.4	96.4	33.2	0.48	0.82	51.5	PASS	
SA020	SAMN56511678	SRR37630431	1,135,818	1,100,840	96.9	96.8	32.7	0.43	1.42	62.8	PASS	
SA021	SAMN56511679	SRR37630430	1,362,590	1,324,218	97.2	97.2	33.2	0.37	1.18	75.3	PASS	
SA022	SAMN56511680	SRR37630428	1,048,338	1,023,584	97.6	96.2	32.9	0.35	0.80	58.2	PASS	
SA023	SAMN56511681	SRR37630427	814,488	805,636	98.9	96.9	33.0	0.30	0.47	52.2	PASS	
SA024	SAMN56511682	SRR37630426	1,361,146	1,322,888	97.2	97.0	33.1	0.43	1.14	75.1	PASS	
SA025	SAMN56511683	SRR37630425	1,138,622	1,108,976	97.4	96.8	33.1	0.44	0.94	64.1	PASS	
SA026	SAMN56511684	SRR37630424	717,158	695,532	97.0	96.7	33.2	0.51	0.81	39.1	PASS	
SA027	SAMN56511685	SRR37630423	1,367,944	1,348,498	98.6	95.1	32.0	0.27	1.98	86.3	PASS	

Notes: Raw Reads, number of read pairs before processing; Trimmed Reads, read pairs retained after Fastp; Retained (%), percentage passing filters; Q30 (%), percentage of bases with Phred ≥30; GC (%), guanine-cytosine content (expected ∼33% for *S. aureus*); Adapter (%), reads with adapter contamination; Dup. (%), estimated PCR/optical duplicate rate; Cov., estimated genome coverage. QC criteria: PASS, all metrics acceptable; WARN, coverage 25–30×.

Table S2. Whole-genome ANI species identification

Whole-genome average nucleotide identity (FastANI v1.34) computed for each assembly against five *S. aureus* reference genomes and *S. epidermidis* RP62A as outgroup. All 26 isolates returned ≥97.28% ANI to NCTC8325 (mean 98.7%), ≥95% to at least one *S. aureus* reference, and no significant ANI to the *S. epidermidis* outgroup, confirming species identity by a fourth orthogonal method.

Sample	ANI to NCTC8325 (%)	Best-match ref.	Best ANI (%)	Species call
SA001	98.96	N315	99.89	*S. aureus*
SA002	98.94	N315	99.92	*S. aureus*
SA004	98.94	N315	99.90	*S. aureus*
SA005	98.88	USA300 FPR3757	98.91	*S. aureus*
SA006	99.70	USA300 FPR3757	99.91	*S. aureus*
SA007	97.28	USA300 FPR3757	97.30	*S. aureus*
SA008	98.27	NCTC8325	98.27	*S. aureus*
SA009	99.01	Mu50	99.05	*S. aureus*
SA010	99.73	USA300 FPR3757	99.98	*S. aureus*
SA011	99.00	USA300 FPR3757	99.01	*S. aureus*
SA012	98.97	N315	99.78	*S. aureus*
SA013	98.79	Mu50	98.79	*S. aureus*
SA014	98.92	N315	99.92	*S. aureus*
SA015	97.37	N315	98.74	*S. aureus*
SA016	98.94	USA300 FPR3757	99.02	*S. aureus*
SA017	98.33	NCTC8325	98.33	*S. aureus*
SA018	99.72	USA300 FPR3757	99.92	*S. aureus*
SA019	98.93	N315	99.91	*S. aureus*
SA020	98.99	NCTC8325	98.99	*S. aureus*
SA021	98.94	N315	99.89	*S. aureus*
SA022	98.77	NCTC8325	98.77	*S. aureus*
SA023	98.91	N315	99.84	*S. aureus*
SA024	98.95	N315	99.89	*S. aureus*
SA025	97.63	MW2	99.86	*S. aureus*
SA026	98.96	N315	99.91	*S. aureus*
SA027	99.79	USA300 FPR3757	99.91	*S. aureus*

References used: *S. aureus* NCTC8325 (RefSeq GCF_000013425.1), N315 (GCF_000009645.1), USA300 FPR3757 (GCF_000013465.1), Mu50 (GCF_000010465.1), MW2 (GCF_000011505.1); outgroup *S. epidermidis* RP62A (GCF_000011865.1). Five samples with ANI to NCTC8325 below the 98.5% within-species expectation (SA007 CC152, SA008 CC22, SA015 CC5, SA017 novel CC22, SA025 CC30) all returned ≥95% ANI to at least one CC-representative reference (Mu50 for CC5; MW2 for CC30; USA300 for CC8/CC152), explained by their phylogenetic distance from the NCTC8325 reference rather than mis-classification. The *S. epidermidis* RP62A reference returned no ANI for any of the 26 query genomes (alignment fraction below the FastANI reporting threshold of 0.20), as expected for genus-level divergence. Per-sample aligned-fraction values for each reference are provided as Supplementary Data on Zenodo (DOI 10.5281/zenodo.19026167).

Table S3. Reference genomes used for contextual phylogeny

Fourteen globally representative *S. aureus* reference genomes were included in the contextual phylogenomic analysis. All genomes were downloaded from NCBI GenBank/RefSeq.

#	Strain	ST	CC	SCC*mec*	Accession	Size (bp)	Key Feature
1	N315	ST5	CC5	II	BA000018.3	2,814,816	HA-MRSA, Japan
2	Mu3	ST5	CC5	II	AP009324.1	2,880,168	hVISA, Japan
3	Mu50	ST5	CC5	II	BA000017.4	2,903,636	VISA, Japan
4	JH1	ST105	CC5	II	CP000736.1	2,906,507	VISA, USA
5	USA300-FPR3757	ST8	CC8	IV	CP000255.1	2,917,469	CA-MRSA, USA (ACME+)
6	NCTC8325	ST8	CC8	—	CP000253.1	2,821,361	MSSA reference
7	EMRSA-15 (HO 5096 0412)	ST22	CC22	IV	HE681097.1	2,832,164	HA-MRSA, UK
8	MW2	ST1	CC1	IV	BA000033.2	2,820,462	CA-MRSA, USA
9	TCH60	ST30	CC30	IV	CP002110.1	2,892,885	CA-MRSA, USA
10	TW20	ST239	CC8	III	FN433596.1	3,043,210	HA-MRSA, UK
11	COL	ST250	CC8	I	CP000046.1	2,813,862	HA-MRSA, UK (1960s)
12	ED98	ST97	CC97	—	NC_013450.1	2,824,404	Bovine MRSA, Ireland
13	JH9	ST105	CC5	II	CP000703.1	2,906,700	VISA, USA
14	S0385	ST398	CC398	—	AM990992.1	2,872,582	LA-MRSA, Netherlands

Abbreviations: ST, sequence type; CC, clonal complex; SCC*mec*, staphylococcal cassette chromosome *mec*; HA-MRSA, healthcare-associated MRSA; CA-MRSA, community-associated MRSA; LA-MRSA, livestock-associated MRSA; hVISA, heterogeneous vancomycin-intermediate *S. aureus*; VISA, vancomycin-intermediate *S. aureus*; ACME, arginine catabolic mobile element.

Table S4. De-identified case-control analytic registry

De-identified analytic registry for the complete case-control set (13 MRSA cases and 39 MSSA controls) used for frequency-matching checks, univariable comparisons, and Firth penalized regression. Group assignment for SA-coded WGS isolates follows the corrected clinical-ID mapping used throughout the revised manuscript; the 26 additional LIS-only isolates are MSSA controls. Patient names and medical-record numbers are not exported. Culture day index is a de-identified ordinal variable where day 1 is the first culture date in the analytic set. Time windows follow the data-collection workbook: prior hospitalization within 12 months, prior antibiotic exposure within 90 days, and surgery within 30 days.

Table S4a. Source, matching, and clinical-context variables.

Study ID	Group	Source	Age	Sex	Month	Day index	Department	Specimen	Clinical syndrome
SA019	MRSA case	WGS cohort	41	M	2025-01	1	Plastic Surgery	Blood	Infective endocarditis
SA018	MRSA case	WGS cohort	41	M	2025-02	31	Otolaryngology (ENT)	Abscess	Neck abscess (HIV infection)
SA005	MRSA case	WGS cohort	53	M	2025-02	37	HIV Unit	Abscess	Extremity abscess (HIV infection)
SA024	MRSA case	WGS cohort	41	M	2025-03	65	Plastic Surgery	Respiratory	Infective endocarditis
SA023	MSSA control	WGS cohort	70	F	2025-04	78	Cardiology	Respiratory	Hospital-acquired pneumonia (HAP) (diabetes mellitus)
SA007	MSSA control	WGS cohort	6	M	2025-04	87	Burn Unit	Tissue	Burn wound infection
SA006	MRSA case	WGS cohort	31	M	2025-04	89	Orthopedics and Traumatology	Abscess	Spider bite–associated skin and soft tissue infection (SSTI)
SA026	MRSA case	WGS cohort	69	M	2025-04	94	Oncology	Urine	Catheter-associated urinary tract infection (CAUTI)
SA014	MRSA case	WGS cohort	77	F	2025-04	97	Geriatrics	Respiratory	Hospital-acquired pneumonia (HAP)
SA001	MRSA case	WGS cohort	75	M	2025-04	96	Geriatrics	Blood	Hospital-acquired pneumonia (HAP)
SA002	MRSA case	WGS cohort	68	M	2025-04	102	Emergency Department	Urine	Abdominal trauma (chronic kidney disease, CKD)
SA004	MRSA case	WGS cohort	24	M	2025-05	113	Orthopedics and Traumatology	Abscess	Skin and soft tissue infection (SSTI)
SA021	MRSA case	WGS cohort	46	M	2025-05	123	General Surgery	Blood	Complicated intra-abdominal infection (cIAI)
SA022	MSSA control	WGS cohort	39	M	2025-04	83	Oncology	Blood	Infective endocarditis (renal tumor)
SA017	MSSA control	WGS cohort	35	F	2025-04	83	Cardiology	Blood	Infective endocarditis (AKD)
SA016	MSSA control	WGS cohort	69	M	2025-04	85	Nephrology	Blood	Hemodialysis arteriovenous fistula infection
SA010	MSSA control	WGS cohort	45	M	2025-04	84	Dermatology	Abscess	Gluteal abscess (HIV infection)
SA009	MSSA control	WGS cohort	34	M	2025-04	85	Neurosurgery	Bone tissue	Vertebral osteomyelitis
SA025	MSSA control	WGS cohort	24	M	2025-04	86	Neurosurgery	Bone tissue	Post-surgical infectious spondylodiscitis
SA008	MSSA control	WGS cohort	18	M	2025-04	87	Burn Unit	Tissue	Phalangeal osteomyelitis
SA011	MSSA control	WGS cohort	48	F	2025-04	89	Emergency Department	Abscess	Pyomyositis
SA012	MSSA control	WGS cohort	51	M	2025-04	94	Neurosurgery	Abscess	Spinal (vertebral) abscess
SA013	MSSA control	WGS cohort	64	M	2025-04	94	Orthopedics and Traumatology	Tissue	Skin and soft tissue infection (SSTI)
SA015	MRSA case	WGS cohort	71	M	2025-04	97	Nephrology	Blood	Hospital-acquired pneumonia (HAP)
SA020	MSSA control	WGS cohort	4	F	2025-05	116	Orthopedics and Traumatology	Bone tissue	Osteomyelitis (site not specified)
SA027	MRSA case	WGS cohort	9	M	2025-05	132	Burn Unit	Biopsy	Burn wound infection
MSSA_CTRL_001	MSSA control	LIS routine	41	M	2025-04	87	General Surgery	Tissue	Surgical site infection
MSSA_CTRL_002	MSSA control	LIS routine	31	M	2025-04	103	Neurosurgery	Blood culture	Primary bacteremia
MSSA_CTRL_003	MSSA control	LIS routine	41	M	2025-05	137	Neurosurgery	Blood culture	Catheter-related infection
MSSA_CTRL_004	MSSA control	LIS routine	70	F	2025-04	101	Neurosurgery	Abscess	Pacemaker pocket abscess
MSSA_CTRL_005	MSSA control	LIS routine	53	M	2025-05	115	General Surgery	Diabetic foot exudate	Diabetic foot
MSSA_CTRL_006	MSSA control	LIS routine	41	M	2025-04	92	Thoracic and Cardiovascular Surgery	Wound	Arteriovenous fistula infection
MSSA_CTRL_007	MSSA control	LIS routine	69	M	2025-05	115	General Surgery	Diabetic foot exudate	Diabetic foot
MSSA_CTRL_008	MSSA control	LIS routine	41	M	2025-06	141	Neurosurgery	Blood culture	Catheter-related infection
MSSA_CTRL_009	MSSA control	LIS routine	31	M	2025-06	157	Cardiology	Peritoneal fluid	Dialysis catheter-associated peritonitis
MSSA_CTRL_010	MSSA control	LIS routine	31	M	2025-03	76	Thoracic and Cardiovascular Surgery	Wound	Arteriovenous fistula infection
MSSA_CTRL_011	MSSA control	LIS routine	43	M	2025-04	80	Neurosurgery	Bronchial secretion	Hospital-acquired pneumonia

(continued)

Study ID	Group	Source	Age	Sex	Month	Day index	Department	Specimen	Clinical syndrome
MSSA_CTRL_012	MSSA control	LIS routine	51	M	2025-04	94	Neurosurgery	Abscess culture	Vertebral abscess
MSSA_CTRL_013	MSSA control	LIS routine	53	M	2025-03	76	Transplant Surgery	Pharyngeal exudate	Pharyngotonsillitis
MSSA_CTRL_014	MSSA control	LIS routine	20	M	2025-04	91	Trauma Surgery	Bone tissue	Open fracture
MSSA_CTRL_015	MSSA control	LIS routine	70	F	2025-05	108	Oncology	Blood culture	Hospital-acquired pneumonia
MSSA_CTRL_016	MSSA control	LIS routine	6	M	2025-04	77	Pediatric ICU	Surgical wound	Ventilator-associated pneumonia
MSSA_CTRL_017	MSSA control	LIS routine	41	M	2025-04	84	HIV Unit	Abscess	Gluteal abscess
MSSA_CTRL_018	MSSA control	LIS routine	69	M	2025-04	98	Hemodialysis	Blood culture	Septic shock
MSSA_CTRL_019	MSSA control	LIS routine	18	M	2025-04	94	Neurosurgery	Peripheral blood culture	Ventilator-associated pneumonia
MSSA_CTRL_020	MSSA control	LIS routine	53	M	2025-04	105	Adult ICU	Blood culture	Ventilator-associated pneumonia + psoas abscess
MSSA_CTRL_021	MSSA control	LIS routine	15	M	2025-06	142	Pediatric Infectious Diseases	Blood culture	Dorsal foot abscess
MSSA_CTRL_022	MSSA control	LIS routine	6	M	2025-04	77	Burn Unit	Blood culture	Primary bacteremia
MSSA_CTRL_023	MSSA control	LIS routine	71	F	2025-04	83	Neurosurgery	Blood culture	Endocarditis
MSSA_CTRL_024	MSSA control	LIS routine	70	M	2025-04	104	Neurosurgery	Blood culture	Primary bacteremia
MSSA_CTRL_025	MSSA control	LIS routine	68	F	2025-04	89	Adult Emergency	Tissue	Left thigh abscess
MSSA_CTRL_026	MSSA control	LIS routine	71	F	2025-05	127	Gynecology and Obstetrics	Urine culture	Urinary tract infection

Table S4b. Variables used in the Firth models and main clinical-outcome comparisons.

Study ID	Group	Prev hosp 12m	ICU	Transfer	LTCF	Prior ATB 90d	Any device	Surgery 30d	Score	Adeq tx	In-hosp death	30d death
SA019	MRSA case	No	Yes	No	No	Yes	Yes	Yes	4	No	Yes	Yes
SA018	MRSA case	No	No	No	No	Yes	No	No	1	No	—	No
SA005	MRSA case	No	No	No	No	Yes	No	No	1	No	—	No
SA024	MRSA case	No	Yes	No	No	Yes	Yes	Yes	4	No	Yes	Yes
SA023	MSSA control	Yes	Yes	Yes	No	Yes	No	No	4	No	Yes	Yes
SA007	MSSA control	No	Yes	No	No	No	No	Yes	2	No	No	No
SA006	MRSA case	No	No	No	Yes	Yes	No	No	2	No	No	No
SA026	MRSA case	Yes	No	No	No	Yes	Yes	Yes	4	No	Yes	Yes
SA014	MRSA case	Yes	Yes	No	No	No	Yes	No	3	No	No	No
SA001	MRSA case	No	Yes	No	No	No	Yes	No	2	No	Yes	Yes
SA002	MRSA case	No	No	No	No	Yes	Yes	Yes	3	No	No	Yes
SA004	MRSA case	No	No	No	No	Yes	No	No	1	No	No	No
SA021	MRSA case	No	Yes	No	No	Yes	Yes	Yes	4	No	No	No
SA022	MSSA control	Yes	No	No	No	Yes	Yes	No	3	Yes	Yes	Yes
SA017	MSSA control	No	No	Yes	No	Yes	Yes	Yes	4	Yes	No	No
SA016	MSSA control	Yes	No	No	No	Yes	Yes	No	3	Yes	No	No
SA010	MSSA control	No	No	No	No	No	No	No	0	No	No	No
SA009	MSSA control	Yes	No	No	No	Yes	No	Yes	3	Yes	No	No
SA025	MSSA control	Yes	No	No	No	No	No	No	1	Yes	No	No
SA008	MSSA control	No	Yes	No	No	No	Yes	Yes	3	Yes	No	No
SA011	MSSA control	No	No	No	No	Yes	No	No	1	Yes	No	No
SA012	MSSA control	No	No	No	No	No	No	No	0	Yes	No	No
SA013	MSSA control	No	No	No	No	No	No	No	0	Yes	No	No
SA015	MRSA case	No	Yes	No	No	No	Yes	No	2	Yes	Yes	Yes
SA020	MSSA control	No	No	No	No	Yes	No	No	1	Yes	No	No
SA027	MRSA case	No	Yes	No	No	Yes	Yes	No	3	Yes	No	No
MSSA_CTRL_001	MSSA control	No	No	No	No	No	No	Yes	1	Yes	No	No
MSSA_CTRL_002	MSSA control	—	Yes	No	No	No	Yes	Yes	3	Yes	No	No
MSSA_CTRL_003	MSSA control	No	Yes	No	No	No	Yes	Yes	3	Yes	No	No
MSSA_CTRL_004	MSSA control	No	—	No	No	No	No	No	0	Yes	No	No
MSSA_CTRL_005	MSSA control	No	No	No	No	Yes	Yes	Yes	3	Yes	No	No
MSSA_CTRL_006	MSSA control	Yes	No	No	No	No	No	Yes	2	Yes	No	No
MSSA_CTRL_007	MSSA control	No	No	No	No	Yes	Yes	Yes	3	Yes	No	No
MSSA_CTRL_008	MSSA control	No	Yes	No	No	No	Yes	Yes	3	Yes	No	No

(continued)

Study ID	Group	Prev hosp 12m	ICU	Transfer	LTCF	Prior ATB 90d	Any device	Surgery 30d	Score	Adeq tx	In-hosp death	30d death
MSSA_CTRL_009	MSSA control	No	No	No	No	No	No	No	0	Yes	No	No
MSSA_CTRL_010	MSSA control	Yes	No	No	No	No	No	Yes	2	Yes	No	No
MSSA_CTRL_011	MSSA control	No	—	No	No	No	Yes	Yes	2	—	No	No
MSSA_CTRL_012	MSSA control	No	No	No	No	No	Yes	Yes	2	Yes	No	No
MSSA_CTRL_013	MSSA control	No	No	No	No	No	No	No	0	Yes	No	No
MSSA_CTRL_014	MSSA control	No	No	No	No	No	No	Yes	1	Yes	No	No
MSSA_CTRL_015	MSSA control	Yes	No	No	No	Yes	No	No	2	Yes	No	Yes
MSSA_CTRL_016	MSSA control	No	Yes	No	No	No	Yes	Yes	3	Yes	No	No
MSSA_CTRL_017	MSSA control	No	No	No	No	No	No	No	0	Yes	No	No
MSSA_CTRL_018	MSSA control	No	No	No	No	Yes	Yes	No	2	Yes	Yes	Yes
MSSA_CTRL_019	MSSA control	No	No	No	No	No	Yes	No	1	Yes	No	No
MSSA_CTRL_020	MSSA control	No	No	No	No	No	Yes	No	1	Yes	Yes	Yes
MSSA_CTRL_021	MSSA control	No	No	No	No	No	No	No	0	Yes	No	No
MSSA_CTRL_022	MSSA control	No	Yes	No	No	Yes	Yes	Yes	4	Yes	No	No
MSSA_CTRL_023	MSSA control	No	No	No	No	No	Yes	No	1	Yes	No	No
MSSA_CTRL_024	MSSA control	No	Yes	No	No	No	Yes	Yes	3	No	Yes	Yes
MSSA_CTRL_025	MSSA control	No	No	No	No	No	No	No	0	Yes	No	No
MSSA_CTRL_026	MSSA control	No	No	Yes	No	No	No	No	1	Yes	No	No

Table S4c. Additional covariates used in univariable comparisons.

Study ID	Group	Diabetes	CKD/HD	HIV	Malignancy	Burn	COPD	Heart failure	CVC	Urinary cath	Ventilation	Bacteremia	LOS days
SA019	MRSA case	No	No	No	No	Yes	No	Yes	Yes	Yes	Yes	Yes	91
SA018	MRSA case	No	No	Yes	No	No	No	No	No	No	No	No	7
SA005	MRSA case	No	No	Yes	No	No	No	No	No	No	No	No	2
SA024	MRSA case	No	No	No	No	Yes	No	Yes	Yes	Yes	Yes	Yes	91
SA023	MSSA control	Yes	No	No	No	No	Yes	No	No	No	No	No	16
SA007	MSSA control	No	No	No	No	Yes	No	No	No	No	No	No	180
SA006	MRSA case	No	No	No	No	No	No	No	No	No	No	No	8
SA026	MRSA case	No	No	No	Yes	No	No	No	No	Yes	No	No	14
SA014	MRSA case	Yes	No	No	No	No	Yes	Yes	Yes	Yes	Yes	No	35
SA001	MRSA case	No	No	No	No	No	No	No	Yes	Yes	Yes	Yes	13
SA002	MRSA case	No	No	No	No	No	No	No	No	Yes	No	No	21
SA004	MRSA case	No	No	No	No	No	No	No	No	No	No	No	12
SA021	MRSA case	No	No	No	No	No	No	No	Yes	Yes	Yes	Yes	32
SA022	MSSA control	No	Yes	No	Yes	No	No	No	Yes	Yes	No	Yes	10
SA017	MSSA control	No	Yes	No	Yes	No	No	Yes	Yes	No	No	Yes	27
SA016	MSSA control	No	Yes	No	No	No	No	No	Yes	Yes	No	Yes	19
SA010	MSSA control	No	No	Yes	No	No	No	No	No	No	No	No	1
SA009	MSSA control	No	No	No	No	No	No	No	No	No	No	No	49
SA025	MSSA control	No	No	No	No	No	No	No	No	No	No	No	30
SA008	MSSA control	No	No	No	No	Yes	No	No	Yes	Yes	9	No	54
SA011	MSSA control	No	No	No	No	No	No	No	No	No	No	No	2
SA012	MSSA control	No	No	No	No	No	No	No	No	No	No	No	31
SA013	MSSA control	Yes	No	No	No	No	No	No	No	No	No	No	13
SA015	MRSA case	No	No	No	No	No	No	No	Yes	Yes	Yes	Yes	14
SA020	MSSA control	No	No	No	No	No	No	No	No	No	No	No	43
SA027	MRSA case	No	No	No	No	Yes	No	No	No	Yes	No	No	58
MSSA_CTRL_001	MSSA control	No	No	No	No	No	No	No	No	No	No	No	8
MSSA_CTRL_002	MSSA control	No	No	No	No	No	No	No	Yes	Yes	Yes	Yes	32
MSSA_CTRL_003	MSSA control	No	No	No	No	No	No	No	Yes	Yes	Yes	No	33
MSSA_CTRL_004	MSSA control	Yes	No	No	No	No	No	Yes	No	No	No	No	7
MSSA_CTRL_005	MSSA control	Yes	No	No	No	No	No	No	No	Yes	No	No	14
MSSA_CTRL_006	MSSA control	No	Yes	No	No	No	No	No	No	No	No	No	4
MSSA_CTRL_007	MSSA control	Yes	No	No	No	No	No	No	No	Yes	No	No	14
MSSA_CTRL_008	MSSA control	No	No	No	No	No	No	No	Yes	Yes	Yes	No	1
MSSA_CTRL_009	MSSA control	No	Yes	No	No	No	No	No	No	No	No	No	1
MSSA_CTRL_010	MSSA control	No	Yes	No	No	No	No	No	No	No	No	No	1
MSSA_CTRL_011	MSSA control	No	No	No	No	No	No	No	No	Yes	No	No	13

(continued)

Study ID	Group	Diabetes	CKD/HD	HIV	Malignancy	Burn	COPD	Heart failure	CVC	Urinary cath	Ventilation	Bacteremia	LOS days
MSSA_CTRL_012	MSSA control	Yes	No	No	No	No	No	No	No	Yes	No	No	30
MSSA_CTRL_013	MSSA control	Yes	Yes	No	No	No	No	No	No	No	No	No	1
MSSA_CTRL_014	MSSA control	No	No	No	No	No	No	No	No	No	No	No	15
MSSA_CTRL_015	MSSA control	No	No	No	Yes	No	Yes	No	No	No	No	No	9
MSSA_CTRL_016	MSSA control	No	No	No	No	No	No	No	No	Yes	Yes	No	11
MSSA_CTRL_017	MSSA control	No	No	No	No	No	No	No	No	No	No	No	1
MSSA_CTRL_018	MSSA control	No	Yes	No	No	No	No	No	Yes	Yes	No	No	14
MSSA_CTRL_019	MSSA control	No	No	No	No	No	No	No	No	No	Yes	No	12
MSSA_CTRL_020	MSSA control	No	No	No	No	No	No	No	Yes	Yes	Yes	No	22
MSSA_CTRL_021	MSSA control	No	No	No	No	No	No	No	No	No	No	No	35
MSSA_CTRL_022	MSSA control	No	No	No	No	Yes	No	No	Yes	Yes	No	Yes	46
MSSA_CTRL_023	MSSA control	No	Yes	No	No	No	No	No	Yes	Yes	No	Yes	27
MSSA_CTRL_024	MSSA control	No	No	No	No	No	No	No	Yes	Yes	Yes	Yes	10
MSSA_CTRL_025	MSSA control	No	No	No	No	No	No	No	No	No	No	No	1
MSSA_CTRL_026	MSSA control	Yes	No	No	No	No	No	No	No	No	No	No	1

Table S5. Complete MLST allelic profiles for 26 *S. aureus* isolates

Multilocus sequence typing (MLST) was performed using the *S. aureus* scheme (7 housekeeping genes). Allelic profiles were determined from assembled genomes using mlst v2.23. Isolates with incomplete allele calls were resolved manually by targeted BLAST.

Table S5. MLST allelic profiles for 26 analyzable *S. aureus* isolates.

**Sample**	**ST**	*arcC*	*aroE*	*glpF*	*gmk*	*pta*	*tpi*	*yqiL*	**CC**
SA001	5	1	4	1	4	12	1	10	CC5
SA002	5	1	4	1	4	12	1	10	CC5
SA004	5	1	4	1	4	12	1	10	CC5
SA005	88	22	1	14	23	12	4	31	CC88
SA006	8	3	3	1	1	4	4	3	CC8
SA007	152	46	75	49	44	13	68	60	CC152
SA008	22	7	6	1	5	8	8	6	CC22
SA009	15	13	13	1	1	12	11	13	CC15
SA010	8	3	3	1	1	4	4	3	CC8
SA011	2867	1	152	1	8	1	5	11	CC8
SA012	5[c]	1	4	1	4	12	1	10	CC5
SA013	188	3	1	1	8	1	1	1	CC188
SA014	5	1	4	1	4	12	1	10	CC5
SA015	5	1	4	1	4	12	1	10	CC5
SA016	4552	3	1	1	37	1	5	611	CC97
SA017	Novel[d]	∼572	6	1	5	8	8	6	CC22
SA018	8	3	3	1	1	4	4	3	CC8
SA019	5	1	4	1	4	12	1	10	CC5
SA020	25	4	1	4	1	5	5	4	CC25
SA021	5	1	4	1	4	12	1	10	CC5
SA022	188	3	1	1	8	1	1	1	CC188
SA023	5	1	4	1	4	12	1	10	CC5
SA024	5	1	4	1	4	12	1	10	CC5
SA025	30	2	2	2	2	6	3	2	CC30
SA026	5	1	4	1	4	12	1	10	CC5
SA027	8	3	3	1	1	4	4	3	CC8

Notes: ST, sequence type; CC, clonal complex. [c]SA012: *pta* exact match to allele 12 but detected twice in assembly (duplication artifact); resolved as ST5/CC5. [d]SA017: *arcC* novel allele (∼572, 1 SNP difference, identity 455/456 bp); SLV of ST22; classified as novel ST within CC22.

Table S6. Patient demographics and clinical characteristics for the 26 sequenced isolates

Anonymized cohort registry: sex, age, infection type, sampling site, hospital department, primary diagnosis, MRSA/MSSA status, and core genomic typing (ST, CC, spa type, SCCmec type, whole-genome ANI to NCTC8325). Ages are exact; departments and specimens are reported as captured in the institutional record.

Table S6. Patient demographics and clinical characteristics for the 26 sequenced isolates.

Clinical ID	Age	Sex	Department	Specimen	Diagnosis	MRSA status	ST	CC	spa type	SCCmec	ANI to NCTC8325 pct
SA001	75	male	Geriatrics	Blood	Hospital-acquired pneumonia (HAP)	MRSA	ST5	CC5	t895	II	98.9556
SA002	68	male	Emergency Department	Urine	Abdominal trauma (chronic kidney disease, CKD)	MRSA	ST5	CC5	t895	II	98.9386
SA004	24	male	Orthopedics and Traumatology	Abscess	Skin and soft tissue infection (SSTI)	MRSA	ST5	CC5	t895	II	98.9371
SA005	53	male	HIV Unit	Abscess	Extremity abscess (HIV infection)	MRSA	ST88	CC88	t13831	IV	98.8791
SA006	31	male	Orthopedics and Traumatology	Abscess	Spider bite–associated skin and soft tissue infection (SSTI)	MRSA	ST8	CC8	t008	IV	99.6977
SA007	6	male	Burn Unit	Tissue	Burn wound infection	MSSA	ST152	CC152	t1096	not applicable	97.2821
SA008	18	male	Burn Unit	Tissue	Phalangeal osteomyelitis	MSSA	ST22	CC22	t223	not applicable	98.2672
SA009	34	male	Neurosurgery	Bone tissue	Vertebral osteomyelitis	MSSA	ST15	CC15	t085	not applicable	99.0052
SA010	45	male	Dermatology	Abscess	Gluteal abscess (HIV infection)	MSSA	ST8	CC8	t008	not applicable	99.7300
SA011	48	female	Emergency Department	Abscess	Pyomyositis	MSSA	ST2867	CC8	t2016	not applicable	99.0011
SA012	51	male	Neurosurgery	Abscess	Spinal (vertebral) abscess	MSSA	ST5	CC5	t010	not applicable	98.9676
SA013	64	male	Orthopedics and Traumatology	Tissue	Skin and soft tissue infection (SSTI)	MSSA	ST188	CC188	t189	not applicable	98.7890
SA014	77	female	Geriatrics	Respiratory	Hospital-acquired pneumonia (HAP)	MRSA	ST5	CC5	t895	II	98.9157
SA015	71	male	Nephrology	Blood	Hospital-acquired pneumonia (HAP)	MRSA	ST5	CC5	t895	II	97.3724
SA016	69	male	Nephrology	Blood	Hemodialysis arteriovenous fistula infection	MSSA	ST4552	CC97	t224	not applicable	98.9385
SA017	35	female	Cardiology	Blood	Infective endocarditis (chronic kidney disease, CKD)	MSSA	Novel*	CC22	t309	not applicable	98.3255
SA018	41	male	Otolaryngology (ENT)	Abscess	Neck abscess (HIV infection)	MRSA	ST8	CC8	t008	IV	99.7207
SA019	41	male	Plastic Surgery	Blood	Infective endocarditis	MRSA	ST5	CC5	t895	II	98.9345
SA020	4	female	Orthopedics and Traumatology	Bone tissue	Osteomyelitis (site not specified)	MSSA	ST25	CC25	t078	not applicable	98.9931
SA021	46	male	General Surgery	Blood	Complicated intra-abdominal infection (cIAI)	MRSA	ST5	CC5	t895	II	98.9383
SA022	39	male	Oncology	Blood	Infective endocarditis (renal tumor)	MSSA	ST188	CC188	t189	not applicable	98.7714
SA023	70	female	Cardiology	Respiratory	Hospital-acquired pneumonia (HAP) (diabetes mellitus)	MSSA	ST5	CC5	t688	not applicable	98.9127
SA024	41	male	Plastic Surgery	Respiratory	Infective endocarditis	MRSA	ST5	CC5	t895	II	98.9536
SA025	24	male	Neurosurgery	Bone tissue	Post-surgical infectious spondylodiscitis	MSSA	ST30	CC30	t012	not applicable	97.6306
SA026	69	male	Oncology	Urine	Catheter-associated urinary tract infection (CAUTI)	MRSA	ST5	CC5	t895	II	98.9559
SA027	9	male	Burn Unit	Biopsy	Burn wound infection	MRSA	ST8	CC8	t008	IV	99.7946

Table S7. Healthcare-associated (HA) versus community-associated (CA) classification per CDC 2013 narrative criteria

CDC 2013 narrative criteria (CDC, MRSA Surveillance Manual, 2013) were applied to each case based on department, specimen, and primary diagnosis. Endocarditis cases without explicit hospital-acquired or community-onset wording are reported as "Indeterminate." Distribution: MRSA 4 HA / 5 CA / 4 indeterminate; MSSA 3 HA / 5 CA / 5 indeterminate.

Table S7. Healthcare-associated (HA) versus community-associated (CA) classification.

Clinical ID	MRSA status	ST	CC	Department	Specimen	Diagnosis	HA CA classification
SA001	MRSA	ST5	CC5	Geriatrics	Blood	Hospital-acquired pneumonia (HAP)	HA
SA002	MRSA	ST5	CC5	Emergency Department	Urine	Abdominal trauma (chronic kidney disease, CKD)	Indeterminate
SA004	MRSA	ST5	CC5	Orthopedics and Traumatology	Abscess	Skin and soft tissue infection (SSTI)	CA
SA005	MRSA	ST88	CC88	HIV Unit	Abscess	Extremity abscess (HIV infection)	CA
SA006	MRSA	ST8	CC8	Orthopedics and Traumatology	Abscess	Spider bite–associated skin and soft tissue infection (SSTI)	CA
SA007	MSSA	ST152	CC152	Burn Unit	Tissue	Burn wound infection	CA
SA008	MSSA	ST22	CC22	Burn Unit	Tissue	Phalangeal osteomyelitis	CA
SA009	MSSA	ST15	CC15	Neurosurgery	Bone tissue	Vertebral osteomyelitis	Indeterminate
SA010	MSSA	ST8	CC8	Dermatology	Abscess	Gluteal abscess (HIV infection)	CA
SA011	MSSA	ST2867	CC8	Emergency Department	Abscess	Pyomyositis	Indeterminate
SA012	MSSA	ST5	CC5	Neurosurgery	Abscess	Spinal (vertebral) abscess	CA
SA013	MSSA	ST188	CC188	Orthopedics and Traumatology	Tissue	Skin and soft tissue infection (SSTI)	CA
SA014	MRSA	ST5	CC5	Geriatrics	Respiratory	Hospital-acquired pneumonia (HAP)	HA
SA015	MRSA	ST5	CC5	Nephrology	Blood	Hospital-acquired pneumonia (HAP)	HA
SA016	MSSA	ST4552	CC97	Nephrology	Blood	Hemodialysis arteriovenous fistula infection	HA
SA017	MSSA	Novel*	CC22	Cardiology	Blood	Infective endocarditis (chronic kidney disease, CKD)	Indeterminate
SA018	MRSA	ST8	CC8	Otolaryngology (ENT)	Abscess	Neck abscess (HIV infection)	CA
SA019	MRSA	ST5	CC5	Plastic Surgery	Blood	Infective endocarditis	Indeterminate
SA020	MSSA	ST25	CC25	Orthopedics and Traumatology	Bone tissue	Osteomyelitis (site not specified)	Indeterminate
SA021	MRSA	ST5	CC5	General Surgery	Blood	Complicated intra-abdominal infection (cIAI)	Indeterminate
SA022	MSSA	ST188	CC188	Oncology	Blood	Infective endocarditis (renal tumor)	Indeterminate
SA023	MSSA	ST5	CC5	Cardiology	Respiratory	Hospital-acquired pneumonia (HAP) (diabetes mellitus)	HA
SA024	MRSA	ST5	CC5	Plastic Surgery	Respiratory	Infective endocarditis	Indeterminate
SA025	MSSA	ST30	CC30	Neurosurgery	Bone tissue	Post-surgical infectious spondylodiscitis	HA
SA026	MRSA	ST5	CC5	Oncology	Urine	Catheter-associated urinary tract infection (CAUTI)	HA
SA027	MRSA	ST8	CC8	Burn Unit	Biopsy	Burn wound infection	CA

Table S8. Clinical outcomes of MRSA versus MSSA infections

Exploratory analysis of clinical outcomes in MRSA versus MSSA infections at Hospital Civil de Guadalajara. Given the small sample size (13 MRSA events), these results should be interpreted as hypothesis-generating. The study was not designed or powered to establish differences in clinical outcomes.

Table S8. Clinical outcomes of MRSA versus MSSA infections (exploratory analysis).

**Outcome**	**MRSA (n = 13)**	**MSSA (n = 39)**	**OR (95% CI)**	***P***
Adequate empiric therapy	2/13 (15.4%)	34/38 (89.5%)	0.02 (0.002–0.16)	<0.001
In-hospital mortality[a]	5/11 (45.5%)	5/39 (12.8%)	5.67 (1.25–22.60)	0.030
30-day mortality	6/13 (46.2%)	6/39 (15.4%)	4.71 (1.16–17.14)	0.051
ICU admission after culture	5/13 (38.5%)	8/39 (20.5%)	2.42 (0.64–8.92)	0.269
Bacteremia	5/13 (38.5%)	7/39 (17.9%)	2.86 (0.55–13.80)	0.147
Hospital stay, days, median (IQR)	14 (12–35)	14 (6–30)	—	0.235

Notes: Fisher's exact test for categorical outcomes; Mann–Whitney U for continuous variables. OR, odds ratio; CI, confidence interval; IQR, interquartile range. [a]In-hospital mortality denominator for MRSA is n = 11 (2 patients lost to follow-up). Bold values indicate statistical significance ($P < 0.05$).

Table S9. Plasmid repertoire for 26 *S. aureus* isolates

Plasmid prediction was performed using MOB-suite on the final Clinical-ID assemblies deposited in GenBank. Mobilization potential (mobilizable, conjugative, non-mobilizable) and replicon types were assigned automatically. SA015 (MRSA, CC5) is flagged as fragmented (1,990 contigs; N50 = 1,721 bp) and is excluded from plasmid-summary statistics because plasmid reconstruction from this assembly is unreliable.

Isolate	Status	Plasmids (n)	Total Size (bp)	Largest (bp)	Mob.	Conj.	Non-mob.	Rep Types
MRSA isolates ($n = 13$)								
SA001	MRSA	6	35,783	12,367	1	0	5	rep_1018
SA002	MRSA	4	18,127	7,859	1	0	3	—
SA004	MRSA	4	15,124	6,055	1	0	3	rep_1837
SA005	MRSA	7	67,070	21,318	3	0	4	rep_1118; rep_1947
SA006	MRSA	4	10,617	3,459	0	0	4	rep_1947
SA014	MRSA	4	23,465	14,544	1	0	3	rep_1018
SA015	MRSA	—	—	—	—	—	—	Excluded (fragmented assembly)
SA018	MRSA	5	51,633	31,426	2	0	3	rep_1017; rep_1118; rep_1215; rep_1733; rep_1947; rep_2100
SA019	MRSA	4	19,891	9,623	1	0	3	rep_1837
SA021	MRSA	4	20,592	9,846	1	0	3	rep_1837
SA024	MRSA	4	15,155	6,055	1	0	3	rep_1837
SA026	MRSA	4	20,167	9,899	1	0	3	rep_1837
SA027	MRSA	4	45,665	38,265	1	0	3	rep_1017; rep_1215; rep_1947
MSSA isolates ($n = 13$)								
SA007	MSSA	3	25,330	20,515	1	0	2	rep_1733; rep_2214
SA008	MSSA	3	38,675	34,978	1	0	2	rep_1017; rep_1215; rep_2214
SA009	MSSA	3	25,214	20,515	1	0	2	rep_1733; rep_2214
SA010	MSSA	5	29,776	20,701	0	0	5	rep_1947
SA011	MSSA	2	4,532	2,933	0	0	2	—
SA012	MSSA	4	37,047	28,623	1	0	3	rep_1017; rep_1215; rep_1281
SA013	MSSA	3	35,115	31,037	1	0	2	rep_1118; rep_1215; rep_1281; rep_2214
SA016	MSSA	6	66,298	27,044	0	0	6	—
SA017	MSSA	3	39,874	35,471	1	0	2	rep_1017; rep_1215; rep_2214
SA020	MSSA	4	24,688	17,334	2	0	2	rep_1017; rep_1733; rep_2100
SA022	MSSA	3	27,970	23,762	1	0	2	rep_1215; rep_2214
SA023	MSSA	3	42,186	38,952	0	1	2	rep_1142
SA025	MSSA	4	57,687	27,877	1	0	3	rep_1017; rep_1733; rep_2100; rep_2214

Notes: Plasmids (n), number of MOB-recon plasmid clusters; Total Size, summed length of predicted plasmid clusters; Largest, length of the largest predicted plasmid cluster; Mob., mobilizable (relaxase+, T4SS−); Conj., conjugative (relaxase+, T4SS+); Non-mob., non-mobilizable (relaxase−, T4SS−). Rep Types, replicon families detected (prefix "rep_cluster_" abbreviated to "rep_"). Summary statistics exclude SA015 because its fragmented assembly precludes reliable plasmid reconstruction. Among the 25 evaluable assemblies, MOB-recon detected 100 plasmid clusters: 75 non-mobilizable (75.0%), 24 mobilizable (24.0%), and one conjugative cluster (1.0%; SA023).

Table S10. Phenotype–genotype concordance for methicillin resistance

Cefoxitin disk diffusion phenotype (CLSI M100, 2024) was concordant with *mecA* genotype in 26/26 (100%) isolates. SA020 was classified as borderline oxacillin-resistant *S. aureus* (BORSA): cefoxitin-negative (MSSA by phenotype and genotype) but oxacillin-resistant (MIC ≥ 4 μg/mL), consistent with *blaZ* hyperproduction.

Table S10. Phenotype–genotype concordance for methicillin resistance in 26 *S. aureus* isolates.

Sample	Cefoxitin	*mecA*	Phenotype	Concordance	Notes
SA001	R	+	MRSA	Yes	
SA002	R	+	MRSA	Yes	
SA004	R	+	MRSA	Yes	
SA005	R	+	MRSA	Yes	
SA006	R	+	MRSA	Yes	
SA007	S	−	MSSA	Yes	
SA008	S	−	MSSA	Yes	
SA009	S	−	MSSA	Yes	
SA010	S	−	MSSA	Yes	
SA011	S	−	MSSA	Yes	
SA012	S	−	MSSA	Yes	
SA013	S	−	MSSA	Yes	
SA014	R	+	MRSA	Yes	
SA015	R	+	MRSA	Yes	
SA016	S	−	MSSA	Yes	
SA017	S	−	MSSA	Yes	
SA018	R	+	MRSA	Yes	
SA019	R	+	MRSA	Yes	
SA020	S	−	MSSA	Yes	BORSA (Oxa R, Cefox S)
SA021	R	+	MRSA	Yes	
SA022	S	−	MSSA	Yes	
SA023	S	−	MSSA	Yes	
SA024	R	+	MRSA	Yes	
SA025	S	−	MSSA	Yes	
SA026	R	+	MRSA	Yes	
SA027	R	+	MRSA	Yes	
**Overall concordance**				**26/26 (100%)**	

Notes: Cefoxitin, disk diffusion result (R, resistant; S, susceptible) per CLSI M100 (2024) breakpoints. *mecA*, detected by read mapping (BWA MEM against SCCmec reference) and confirmed by assembly screening (AMRFinderPlus, ResFinder). BORSA, borderline oxacillin-resistant *S. aureus*; SA020 is cefoxitin-susceptible and *mecA*-negative (genotypic MSSA) but oxacillin-resistant, consistent with *blaZ* hyperproduction.

Table S11. Antimicrobial resistance gene profiles for 26 *S. aureus* isolates

Resistance genes were detected using AMRFinderPlus, CARD, and ResFinder databases with ≥80% identity and ≥80% coverage thresholds. QRDR point mutations (*gyrA*, *parC*) were identified by AMRFinderPlus. All genes screened are shown, including those negative in all isolates, to document comprehensive screening.

		OXA		PEN	MLS$_B$				Mac		CIP		TET				SXT			VAN		LZD		MUP		Aminoglycosides				FUS	
**Sample**	**Status**	*mecA*	*mecC*	*blaZ*	*ermA*	*ermB*	*ermC*	*ermT*	*msrA*	*mphC*	*gyrA*[a]	*parC*[b]	*tet(38)*[c]	*tetK*	*tetM*	*tetL*	*dfrG*	*dfrA*	*dfrK*	*vanA*	*vanB*	*cfr*	*optrA*	*mupA*	*mupB*	*aacA-aphD*	*aadD*	*aph(3')-IIIa*	*ant(6')-Ia*	*fusB*	*fusC*
MRSA isolates (n = 13)																															
SA001	MRSA	+	−	−	+	−	−	−	−	−	S84L	S80F	+	−	−	−	−	−	−	−	−	−	−	−	−	+	−	−	−	−	−
SA002	MRSA	+	−	+	+	−	−	−	−	−	S84L	S80Y	+	−	−	−	−	−	−	−	−	−	−	−	−	+	−	−	−	−	−
SA004	MRSA	+	−	+	+	−	−	−	−	−	S84L	S80F	+	−	−	−	−	−	−	−	−	−	−	−	−	+	−	−	−	−	−
SA005	MRSA	+	−	+	−	−	+	−	−	−	−	−	+	−	−	−	−	−	−	−	−	−	−	−	−	+	−	+	−	−	−
SA006	MRSA	+	−	+	−	−	+	−	−	−	−	S80F	+	−	−	−	−	−	−	−	−	−	−	−	−	−	−	+	−	−	−
SA014	MRSA	+	−	+	+	−	−	−	−	−	S84L	S80Y	+	−	−	−	−	−	−	−	−	−	−	−	−	+	−	−	−	−	−
SA015	MRSA	+	−	+	+	−	−	−	−	−	−	−	+	−	−	−	−	−	−	−	−	−	−	−	−	+	−	−	−	−	−
SA018	MRSA	+	−	+	−	−	+	−	+	+	S84L	S80F	+	−	−	−	−	−	−	−	−	−	−	+	−	+	−	+	−	−	−
SA019	MRSA	+	−	+	+	−	−	−	−	−	S84L	S80F	+	−	−	−	−	−	−	−	−	−	−	−	−	−	+	−	−	−	−
SA021	MRSA	+	−	+	+	−	−	−	−	−	−	−	+	−	−	−	−	−	−	−	−	−	−	−	−	+	−	−	−	−	−
SA024	MRSA	+	−	+	+	−	−	−	−	−	S84L	S80F	+	−	−	−	−	−	−	−	−	−	−	−	−	+	−	−	−	−	−
SA026	MRSA	+	−	+	+	−	−	−	−	−	S84L	S80F	+	−	−	−	−	−	−	−	−	−	−	−	−	+	−	−	−	−	−
SA027	MRSA	+	−	+	−	−	+	−	−	−	−	−	+	−	−	−	−	−	−	−	−	−	−	−	−	−	−	−	−	−	−
MSSA isolates (n = 13)																															
SA007	MSSA	−	−	+	−	−	−	−	−	−	−	−	+	−	−	−	−	−	−	−	−	−	−	−	−	−	−	−	−	−	−
SA008	MSSA	−	−	+	−	−	−	−	−	−	−	−	+	−	−	−	−	−	−	−	−	−	−	−	−	−	−	−	−	−	−
SA009	MSSA	−	−	+	−	−	−	−	−	−	S84L	S80Y	+	−	−	−	−	−	−	−	−	−	−	−	−	−	−	−	−	−	−
SA010	MSSA	−	−	−	−	−	+	−	−	−	−	−	+	−	−	−	−	−	−	−	−	−	−	−	−	−	−	−	−	−	−
SA011	MSSA	−	−	−	−	−	−	−	−	−	−	−	+	−	−	−	−	−	−	−	−	−	−	−	−	−	−	−	−	−	−
SA012	MSSA	−	−	+	−	−	−	−	−	−	−	−	+	−	−	−	−	−	−	−	−	−	−	−	−	−	−	−	−	−	−
SA013	MSSA	−	−	+	−	−	−	−	−	−	−	−	+	−	−	−	−	−	−	−	−	−	−	−	−	+	−	−	−	−	−
SA016	MSSA	−	−	+	−	−	−	−	−	−	−	−	+	−	−	−	−	−	−	−	−	−	−	−	−	−	−	−	−	−	−
SA017	MSSA	−	−	+	−	−	−	−	−	−	−	S80F	+	+	−	−	−	−	−	−	−	−	−	−	−	−	−	−	−	−	−
SA020	MSSA	−	−	+	−	−	−	−	−	−	S84L	S80F	+	−	−	−	−	−	−	−	−	−	−	−	−	−	−	−	−	−	−
SA022	MSSA	−	−	+	−	−	−	−	−	−	S84L	S80F	+	−	−	−	−	−	−	−	−	−	−	−	−	+	−	−	−	−	−
SA023	MSSA	−	−	−	−	−	−	−	−	−	−	−	+	−	−	−	−	−	−	−	−	−	−	+	−	+	−	−	−	−	−
SA025	MSSA	−	−	+	−	−	−	−	−	−	−	−	+	−	−	−	−	−	−	−	−	−	−	−	−	−	−	−	−	−	−
**Total +**		13	0	22	8	0	4	0	1	1	11	13	26	1	0	0	0	0	0	0	0	0	0	2	0	11	1	3	0	0	0

Notes: +, gene detected; −, gene not detected; S84L, S80F, S80Y, specific amino acid substitutions in QRDR regions. [a] *gyrA* S84L: DNA gyrase subunit A (Ser84→Leu); detected in 11 isolates (8 MRSA, 3 MSSA). [b] *parC* S80F/S80Y: DNA topoisomerase IV subunit A (Ser80→Phe or Ser80→Tyr); detected in 13 isolates. SA009 additionally harbored *parE* P585S. [c] *tet(38)*: intrinsic chromosomal tetracycline efflux pump universal in *S. aureus*; detected in all 26 isolates as expected. Drug class abbreviations: OXA, oxacillin; PEN, penicillin; MLS$_B$, macrolide-lincosamide-streptogramin B; Mac, macrolide efflux/phosphotransferase; CIP, ciprofloxacin; TET, tetracycline; SXT, trimethoprim-sulfamethoxazole; VAN, vancomycin; LZD, linezolid; MUP, mupirocin; FUS, fusidic acid. SA017 additionally harbored *lnu(A)* (lincosamide nucleotidyltransferase) and *tetK*. Additional genes screened but not detected: *fexA*, *rpoB* mutations, *msrB*, *cat*, *tetO*.

Table S12. Genomic markers related to SCC*mec* burden and colonization in MRSA versus MSSA

Genomic markers related to SCC*mec* burden and colonization were compared between MRSA (n = 13) and MSSA (n = 13) isolates. SCC*mec*-associated burden was classified as higher (type II, ~53 kb), lower (type IV, ~24 kb), or none based on cassette type, size, and gene content; no growth or competition assays were performed in this study.

S12a. Per-isolate genomic marker profile

Table S12a. Per-isolate SCC*mec* burden, ACME, complete IEC, PVL, and virulence gene counts.

Isolate	Status	CC	SCC*mec*	Size (kb)	Burden	ACME	IEC complete	PVL	Vir (*n*/94)
SA001	MRSA	CC5	II	~53	Higher	−	+	−	74
SA002	MRSA	CC5	II	~53	Higher	−	+	−	78
SA004	MRSA	CC5	II	~53	Higher	−	+	−	78
SA005	MRSA	CC88	IV	~24	Lower	−	+	+	82
SA006	MRSA	CC8	IV	~24	Lower	−	−	+	80
SA014	MRSA	CC5	II	~53	Higher	−	+	−	78
SA015	MRSA	CC5	II	~53	Higher	−	−	−	78
SA018	MRSA	CC8	IV	~24	Lower	−	+	+	82
SA019	MRSA	CC5	II	~53	Higher	−	+	−	78
SA021	MRSA	CC5	II	~53	Higher	−	+	−	78
SA024	MRSA	CC5	II	~53	Higher	−	+	−	78
SA026	MRSA	CC5	II	~53	Higher	−	+	−	78
SA027	MRSA	CC8	IV	~24	Lower	−	−	+	72
SA007	MSSA	CC152	—	0	None	−	−	+	81
SA008	MSSA	CC22	—	0	None	−	−	−	69
SA009	MSSA	CC15	—	0	None	−	+	−	71
SA010	MSSA	CC8	—	0	None	−	−	+	82
SA011	MSSA	CC8	—	0	None	−	−	−	78
SA012	MSSA	CC5	—	0	None	−	+	−	78
SA013	MSSA	CC188	—	0	None	−	+	−	80
SA016	MSSA	CC97	—	0	None	−	+	−	73
SA017	MSSA	CC22	—	0	None	−	−	−	67
SA020	MSSA	CC25	—	0	None	−	+	−	79
SA022	MSSA	CC188	—	0	None	−	+	−	80
SA023	MSSA	CC5	—	0	None	−	−	−	57
SA025	MSSA	CC30	—	0	None	−	+	−	75

S12b. MRSA vs MSSA comparative summary

Table S12b. MRSA versus MSSA comparative summary of genomic markers related to SCC*mec* burden and colonization.

**Genomic marker**	**MRSA (n = 13)**	**MSSA (n = 13)**	***P***	**Test**
SCCmec burden				
Higher (type II, ∼53 kb)	9/13 (69.2%)	0/13 (0%)	<0.001	Fisher's
Lower (type IV, ∼24 kb)	4/13 (30.8%)	0/13 (0%)	0.098	Fisher's
None	0/13 (0%)	13/13 (100%)	<0.001	Fisher's
ACME	0/13 (0%)	0/13 (0%)	1.000	Fisher's
Capsule CP8	13/13 (100%)	13/13 (100%)	1.000	Fisher's
IEC complete (*scn*+*chp*+*sak*)	10/13 (76.9%)	7/13 (53.8%)	0.411	Fisher's
PVL (*lukS-PV*/*lukF-PV*)	4/13 (30.8%)	2/13 (15.4%)	0.645	Fisher's
Virulence genes (VFDB)				
Mean ± SD	77.8 ± 2.8	73.1 ± 7.1	0.080	Mann–Whitney
Range	72–82	57–82		
ST diversity	3 STs	11 STs	—	—
Simpson's 1-D	0.500	0.974	—	—
Pielou's J′	0.719	0.981	—	—

Notes: SCC*mec*-associated burden categories are literature-derived genomic inferences based on cassette type, size, and gene content (Lee et al. 2007; Okuma et al. 2002), not direct fitness measurements in this isolate collection. ACME, arginine catabolic mobile element; screened by BLASTn for *arcA* and *opp3* against USA300 (CP000255). Capsule typing: cap5 vs cap8 gene clusters (VFDB). IEC complete: simultaneous detection of *scn*, *chp*, and *sak*; partial IEC configurations are reported in Table S15. PVL: both *lukS-PV* and *lukF-PV* detected. Virulence gene counts: total genes detected by ABRicate/VFDB (≥80% identity and coverage; 94 unique genes screened). Statistical tests: Fisher's exact (categorical), Mann–Whitney U (continuous), two-sided, $\alpha = 0.05$.

Table S13. Pairwise SNP distance matrix for 26 *S. aureus* isolates

Pairwise core-genome SNP distances were calculated using snp-dists on the recombination-filtered Snippy core alignment (reference: *S. aureus* N315, BA000018.3). Due to the large size of the 26×26 matrix, only the CC5-MRSA-SCC*mec* II cluster (n = 9 MRSA + SA023 MSSA) and representative between-lineage distances are shown below. The full matrix is available as Supplementary Data.

Table S13a. Intra-CC5 SNP distances (MRSA-SCC*mec* II cluster + SA023).

	**SA019**	**SA002**	**SA004**	**SA026**	**SA014**	**SA001**	**SA024**	**SA021**	**SA015**	**SA023**
SA019	0	7	3	13	45	51	493	363	205	97
SA002	7	0	4	12	46	52	494	362	206	94
SA004	3	4	0	10	42	48	490	360	202	94
SA026	13	12	10	0	46	58	500	368	212	102
SA014	45	46	42	46	0	18	532	396	244	136
SA001	51	52	48	58	18	0	530	408	248	130
SA024	493	494	490	500	532	530	0	850	692	562
SA021	363	362	360	368	396	408	850	0	562	448
SA015	205	206	202	212	244	248	692	562	0	294
SA023	97	94	94	102	136	130	562	448	294	0

Table S13b. Representative between-lineage SNP distances (selected pairs).

**Pair**	**Lineages**	**SNP distance**
SA019 vs SA018	CC5 vs CC8	6,187
SA019 vs SA007	CC5 vs CC152	6,167
SA019 vs SA009	CC5 vs CC15	6,330
SA019 vs SA016	CC5 vs CC97	6,495
SA019 vs SA025	CC5 vs CC30	30,409
SA019 vs SA027	CC5 vs CC8 (USA300-like)	37,077
SA018 vs SA027	CC8 vs CC8	39,100
SA008 vs SA017	CC22 vs CC22	156
SA013 vs SA022	CC188 vs CC188	69

Notes: Values represent the number of core-genome SNP differences after recombination filtering. Low intra-CC5 distances (3–58 SNPs among SA019/SA002/SA004/SA026/SA014/SA001) are compatible with recent clonal spread. SA023 (MSSA, 94–562 SNPs from MRSA-CC5 isolates) nests within the CC5 clade, consistent with SCC*mec* excision as a working hypothesis. Between-lineage distances (>6,000 SNPs) reflect deep phylogenetic divergence. The full 26×26 matrix is provided as Supplementary Data.

Table S14. Pan-genome composition of 26 *S. aureus* isolates

Pan-genome analysis was performed with Roary v3.13.0 using a 95% amino acid identity threshold on Prokka-annotated genomes.

Table S14. Pan-genome composition of 26 *S. aureus* isolates (Roary v3.13.0, 95% identity).

**Category**	**Gene clusters**	**Proportion (%)**
Core (≥99% prevalence)	1,168	19.2
Soft-core (95–99%)	766	12.6
Shell (15–95%)	1,174	19.3
Cloud (<15%)	2,960	48.8
**Total**	**6,068**	**100.0**

Notes: Core genes are present in ≥99% of isolates; soft-core in 95–99%; shell in 15–95%; cloud in <15%. The core genome proportion (19.2%) is concordant with published *S. aureus* pan-genome analyses (e.g., 19.1% core in 1,519 genomes; Liu et al. 2022, *Microbiol. Spectr.*).

Table S15. Prophage content of 26 *S. aureus* isolates

Prophage regions were detected by PhiSpy v5.0.2 with the *S. aureus*-specific training set. Virulence genes were identified by cross-referencing Prokka annotations with known prophage-associated genes. A total of 136 prophage regions were detected (mean 5.2 per genome).

**Sample**	**Status**	**Regions**	**Intact**	**Quest.**	**Incomp.**	**Total (bp)**	**Virulence genes**	**Inferred prophage types**
MRSA isolates (n = 13)								
SA001	MRSA	4	0	2	2	51,266	*chp; sak; scn*	φSa3int (IEC)
SA002	MRSA	4	0	4	0	60,422	*chp; sak; scn*	φSa3int (IEC)
SA004	MRSA	4	0	2	2	53,336	*chp; sak; scn*	φSa3int (IEC)
SA005	MRSA	1	0	0	1	7,360	*chp; sak; scn*	φSa3int (IEC)
SA006	MRSA	5	0	2	3	49,416	*sak; scn*	φSa3int (partial IEC)
SA014	MRSA	5	0	3	2	69,317	*chp; sak; scn*	φSa3int (IEC)
SA015	MRSA	8	0	3	5	71,382	*etb; sak; scn*	φSa3int; φETA
SA018	MRSA	5	0	3	2	60,116	*chp; sak; scn*	φSa3int (IEC)
SA019	MRSA	6	1	3	2	91,493	*chp; sak; scn*	φSa3int (IEC)
SA021	MRSA	7	1	2	4	86,423	*chp; sak; scn*	φSa3int (IEC)
SA024	MRSA	4	1	1	2	58,954	*chp; sak; scn*	φSa3int (IEC)
SA026	MRSA	4	0	2	2	57,816	*chp; sak; scn*	φSa3int (IEC)
SA027	MRSA	3	0	1	2	42,068	*etb; sak; scn*	φSa3int; φETA
MSSA isolates (n = 13)								
SA007	MSSA	6	0	2	4	46,317	*scn; tst*	φSa3int; SaPI (*tst*)
SA008	MSSA	9	0	5	4	98,264	*scn*	φSa3int (partial)
SA009	MSSA	5	0	2	3	55,084	*chp; sak; scn*	φSa3int (IEC)
SA010	MSSA	6	0	3	3	70,302	*chp; scn*	φSa3int (partial IEC)
SA011	MSSA	7	0	3	4	63,724	*sak; scn; tst*	φSa3int; SaPI (*tst*)
SA012	MSSA	5	0	2	3	46,205	*chp; sak; scn*	φSa3int (IEC)
SA013	MSSA	4	0	4	0	64,143	*chp; sak; scn*	φSa3int (IEC)
SA016	MSSA	5	0	4	1	75,721	*chp; sak; scn*	φSa3int (IEC)
SA017	MSSA	5	0	4	1	64,456	*sak; scn*	φSa3int (partial IEC)
SA020	MSSA	6	0	0	6	35,367	*chp; sak; scn*	φSa3int (IEC)
SA022	MSSA	3	0	2	1	48,674	*chp; sak; scn*	φSa3int (IEC)
SA023	MSSA	8	0	4	4	88,457	*sak; scn*	φSa3int (partial IEC)
SA025	MSSA	6	0	3	3	57,572	*chp; etb; sak; scn*	φSa3int (IEC); φETA

Notes: Intact, $\geq$30 kb with identifiable head/tail; Quest., questionable (10–30 kb); Incomp., <10 kb or fragmented. φSa3int: β-hemolysin-converting prophage carrying IEC (*scn*$\pm$*chp*$\pm$*sak*$\pm$*sea*). φETA: exfoliative toxin B-carrying prophage. SaPI: staphylococcal pathogenicity island. *tst*, toxic shock syndrome toxin 1 (detected in SA007 and SA011). Most prophage regions were incomplete or questionable due to assembly fragmentation inherent to short-read sequencing.

Table S16. Insertion sequence repertoire of 26 *S. aureus* isolates

Insertion sequences were screened from Prokka annotations. IS431/IS257 (IS6 family) flanks SCC*mec* and mediates its excision/integration.

Table S16. Insertion sequence (IS) element summary for 26 *S. aureus* isolates.

Sample	Status	Total IS	IS431	IS256	IS1272	IS families
MRSA (n = 13)						
SA001	MRSA	6	2	0	0	IS1182, IS3, IS6, ISL3
SA002	MRSA	10	0	1	0	IS110, IS1182, IS1595, IS256, IS3, IS30, IS605
SA004	MRSA	6	2	0	0	IS1182, IS3, IS6, ISL3
SA005	MRSA	8	1	0	0	IS1182, IS3, IS30, IS6, ISL3, ISNCY
SA006	MRSA	9	1	0	0	IS1182, IS30, IS6, ISNCY
SA014	MRSA	9	1	0	0	IS110, IS1182, IS3, IS30, IS6, IS605
SA015	MRSA	6	0	0	0	IS1182, IS605, ISL3
SA018	MRSA	6	2	0	0	IS1182, IS3, IS6, ISL3
SA019	MRSA	4	0	0	0	IS1182, IS3, ISNCY
SA021	MRSA	8	1	0	0	IS1182, IS3, IS6, ISL3, ISNCY
SA024	MRSA	6	2	0	0	IS1182, IS3, IS6, ISL3
SA026	MRSA	7	2	0	0	IS1182, IS3, IS6, ISL3, ISNCY
SA027	MRSA	5	1	1	0	IS256, IS30, IS6, ISL3, ISNCY
MSSA (n = 13)						
SA007	MSSA	15	0	0	0	IS1182, IS21, IS3, IS30, IS6, IS605, ISL3
SA008	MSSA	4	0	0	0	IS1182, IS30, ISL3
SA009	MSSA	5	0	0	0	IS110, IS3, IS30
SA010	MSSA	4	0	1	0	IS256, IS30, IS605, ISNCY
SA011	MSSA	9	0	0	0	IS1182, IS3, IS30, IS6, ISNCY
SA012	MSSA	15	0	0	0	IS1182, IS21, IS3, IS30, IS6, IS605, ISL3
SA013	MSSA	5	0	1	0	IS1182, IS256, IS30, IS605
SA016	MSSA	4	2	0	0	IS1182, IS3, IS6
SA017	MSSA	9	2	0	0	IS1182, IS30, IS6, ISNCY
SA020	MSSA	4	0	0	0	IS1182, IS3, ISNCY
SA022	MSSA	6	2	0	0	IS1182, IS3, IS6, ISL3
SA023*	MSSA	1	0	0	0	IS6 (1 IS257-1 on plasmid contig_23)
SA025	MSSA	4	0	0	0	IS1182, IS3, IS605, ISNCY
MRSA prevalence			10/13 (77%)	2/13 (15%)	0/13	
MSSA prevalence			3/13 (23%)	2/13 (15%)	0/13	

IS431/IS257 (IS6 family) flanks SCC*mec* and mediates cassette excision/integration. ***SA023 (SCC*mec* excision candidate) carries a single IS6-family element (IS257-1) located on a separate 18,417-bp plasmid contig (SA023_contig_23, GC 29.3%) that also encodes *mupA*, *ssb*, and *topB*; no IS431/IS257-family elements are present within ±50 kb of *orfX*/*attB* on the chromosomal contig.** The chromosomal flanking architecture (*walK*/*walR*/*yycJ* upstream; *glpE*/*gloB*/*dus* downstream) is identical to the canonical MSSA reference NCTC8325, consistent with precise *ccr*AB-mediated excision rather than IS-mediated recombination. IS counts and family assignments derive from NCBI-deposited Prokka annotations (BioProject PRJNA1437481); IS256 is associated with biofilm formation; IS1272 associates with SCC*mec* type I (not present in this collection). See Fig. S4 for the read-coverage track and Table S17 for direct comparison to the 9 sibling CC5-MRSA isolates.

Table S17. Comparative analysis of the *mec* complex across SA023 and 9 CC5-MRSA siblings

Direct query of the NCBI-deposited Prokka annotations (BioProject PRJNA1437481) for mecA, mecR1, mecI, ccrA, and ccrB across the 10 CC5 isolates in this study.

Table S17. Presence/absence of the canonical SCC*mec* II core genes in CC5 isolates (NCBI annotations, BioProject PRJNA1437481).

Isolate	mecA	mecR1	mecI	ccrA	ccrB	Status
SA001	+	+	+	+	+	MRSA-CC5-SCC*mec* II
SA002	+	+	+	+	+	MRSA-CC5-SCC*mec* II
SA004	+	+	+	+	+	MRSA-CC5-SCC*mec* II
SA014	+	+	+	+	+	MRSA-CC5-SCC*mec* II
SA015	+	+	+	+	+	MRSA-CC5-SCC*mec* II
SA019	+	+	+	+	+	MRSA-CC5-SCC*mec* II
SA021	+	+	+	+	+	MRSA-CC5-SCC*mec* II
SA024	+	+	+	+	+	MRSA-CC5-SCC*mec* II
SA026	+	+	+	+	+	MRSA-CC5-SCC*mec* II
**SA023**	−	−	−	−	−	**MSSA-CC5-t688 (excision candidate)**

All 9 sibling MRSA-CC5 isolates carry the complete mecA-PBP2a + mecR1 + mecI regulatory complex plus the ccrA/ccrB recombinase pair within their SCCmec II cassettes. SA023, sharing the same chromosomal background and spa-related typing (spa t688 vs t895), uniquely carries zero of these mec-complex genes. This pattern, together with the intact attB/orfX locus and the read-coverage data in Fig. S4, is consistent with precise ccrAB-mediated cassette excision.

Table S18. Core genome MLST schema and allelic distances

A *de novo* wgMLST schema was generated from 26 *S. aureus* assemblies using chewBBACA v3.5.3. Allelic profiles were extracted and pairwise distances computed.

Table S18a. wgMLST/cgMLST schema composition.

Category	Loci	Proportion (%)
Strict core (≥99%)	546	15.9
Soft-core (95–99%)	995	28.9
Shell (15–95%)	1,897	55.2
**Total wgMLST**	**3,438**	**100.0**
Paralogous (excluded)	17	—

Schema created with chewBBACA v3.5.3 (BLAST+ 2.16.0, Prodigal v2.6.3, translation table 11). cgMLST distances computed on the 546 strict-core loci. Correlation with SNP distances: Spearman $\rho = 0.327$ ($P < 0.001$). The *de novo* schema provides intra-lineage resolution but limited inter-lineage discrimination due to the small sample size; a validated reference schema (cgMLST.org, 1,861 loci) is recommended for cross-institutional comparisons.

Table S19. Anti-phage defense systems in 26 *S. aureus* isolates

Defense systems were identified using DefenseFinder v2.0.1 on Prokka-predicted protein sequences. A total of 209 systems comprising 23 subtypes were detected across 26 isolates.

**Sample**	**Status**	**CC**	**SCC*mec***	**Total**	**RM**	**CRISPR**	**Defense systems detected**
MRSA isolates (n = 13)							
SA001	MRSA	CC5	II	8	2	0	Abi2, FS_Sma, gcu233, Retron_III, RM_Type_I, RosmerTA, Stk2
SA002	MRSA	CC5	II	6	0	0	Abi2, AbiD, Avs_II, Dodola, FS_Sma, gcu233
SA004	MRSA	CC5	II	8	2	0	Abi2, FS_Sma, gcu233, Retron_III, RM_Type_I, RosmerTA, Stk2
SA005	MRSA	CC88	IV	9	2	0	Abi2, AbiD, FS_Sma, gcu233, Retron_III, RM_Type_I, RM_Type_IV, RosmerTA, Stk2
SA006	MRSA	CC8	IV	10	4	0	Abi2, AbiAlpha, AbiD, AbiJ, gcu233, RM_Type_I, RM_Type_II, RM_Type_IV, RosmerTA
SA014	MRSA	CC5	II	5	0	0	Abi2, Avs_II, Dodola, FS_Sma, gcu233
SA015	MRSA	CC5	II	6	1	0	Abi2, AbiD, Gabija, gcu233, RM_Type_I
SA018	MRSA	CC8	IV	8	2	0	Abi2, FS_Sma, gcu233, Retron_III, RM_Type_I, RosmerTA, Stk2
SA019	MRSA	CC5	II	8	2	0	Abi2, FS_Sma, gcu233, Retron_III, RM_Type_I, RosmerTA, Stk2
SA021	MRSA	CC5	II	9	3	0	Abi2, AbiAlpha, FS_Sma, gcu233, Retron_III, RM_Type_I, RosmerTA
SA024	MRSA	CC5	II	8	2	0	Abi2, FS_Sma, gcu233, Retron_III, RM_Type_I, RosmerTA, Stk2
SA026	MRSA	CC5	II	8	2	0	Abi2, FS_Sma, gcu233, Retron_III, RM_Type_I, RosmerTA, Stk2
SA027	MRSA	CC8	IV	5	1	0	AbiD, Gabija, gcu233, RM_Type_I, SoFic
MSSA isolates (n = 13)							
SA007	MSSA	CC152	—	8	2	0	Abi2, gcu233, Pycsar, RM_Type_I, ShosTA
SA008	MSSA	CC22	—	15	5	0	Abi2, AbiAlpha, FS_Sma, gcu233, PD-Lambda-1, Pycsar, RM_Type_I, RosmerTA
SA009	MSSA	CC15	—	6	0	0	Abi2, AbiD, Avs_II, Dodola, FS_Sma, gcu233
SA010	MSSA	CC8	—	9	3	0	Abi2, FS_Sma, gcu233, Lamassu-Cap4, RM_Type_I, RM_Type_II, RosmerTA
SA011	MSSA	CC8	—	10	3	0	Abi2, AbiD, FS_Sma, gcu233, PD-Lambda-1, RloC, RM_Type_I, RM_Type_IV, RosmerTA
SA012	MSSA	CC5	—	9	2	0	Abi2, FS_Sma, gcu233, Pycsar, RM_Type_I, ShosTA
SA013	MSSA	CC188	—	7	0	0	Abi2, AbiD, AbiJ, Avs_II, Dodola, FS_Sma, gcu233
SA016	MSSA	CC97	—	7	2	0	Abi2, FS_Sma, gcu233, Retron_III, RM_Type_I, RosmerTA
SA017	MSSA	CC22	—	9	4	0	Abi2, AbiD, AbiJ, gcu233, RM_Type_I, RM_Type_II, RM_Type_IV, RosmerTA
SA020	MSSA	CC25	—	7	2	0	Abi2, FS_Sma, gcu233, RM_Type_I, RosmerTA, Stk2
SA022	MSSA	CC188	—	8	2	0	Abi2, FS_Sma, gcu233, Retron_III, RM_Type_I, RosmerTA, Stk2
SA023	MSSA	CC5	—	8	2	0	Abi2, AbiD, CoCoNut_I-B, gcu233, RM_Type_I, RosmerTA
SA025	MSSA	CC30	—	8	3	0	Abi2, AbiD, AbiJ, AbiK, gcu233, RM_Type_I, RM_Type_II, RM_Type_IV
**Mean (MRSA)**				**7.5**	1.8	0	
**Mean (MSSA)**				**8.5**	2.3	0	

Notes: Defense systems detected by DefenseFinder v2.0.1. Total, number per isolate; RM, restriction-modification; CRISPR, CRISPR-Cas (none detected). Core: gcu233 (26/26), Abi2 (25/26), RM_Type_I (22/26). Retron_III enriched in MRSA (8/13, 62%) vs MSSA (2/13, 15%). Pycsar, ShosTA, PD-Lambda-1 exclusive to MSSA. SA008 (CC22) outlier with 15 systems.

Table S20. Catalogue of resistance, virulence, and anti-phage defense genes/systems detected in the 26 isolates

Combined catalogue including 188 entries spanning antimicrobial-resistance determinants, virulence-associated genes, stress/resistance markers, and anti-phage defense systems. DefenseFinder identified 23 defense-system subtypes across the cohort (Table S19).

Table S20. Catalogue of resistance, virulence, and anti-phage defense genes/systems detected in the 26 isolates.

Gene or System	Type	Class	Subclass	Product or Function	Database source
Abi2	DEFENSE	DefenseFinder		Anti-phage defense system: Abi2	DefenseFinder
AbiAlpha	DEFENSE	DefenseFinder		Anti-phage defense system: AbiAlpha	DefenseFinder
AbiD	DEFENSE	DefenseFinder		Anti-phage defense system: AbiD	DefenseFinder
AbiJ	DEFENSE	DefenseFinder		Anti-phage defense system: AbiJ	DefenseFinder
AbiK	DEFENSE	DefenseFinder		Anti-phage defense system: AbiK	DefenseFinder
Avs_II	DEFENSE	DefenseFinder		Anti-phage defense system: Avs_II	DefenseFinder
CoCoNut_I-B	DEFENSE	DefenseFinder		Anti-phage defense system: CoCoNut_I-B	DefenseFinder
Dodola	DEFENSE	DefenseFinder		Anti-phage defense system: Dodola	DefenseFinder
FS_Sma	DEFENSE	DefenseFinder		Anti-phage defense system: FS_Sma	DefenseFinder
Gabija	DEFENSE	DefenseFinder		Anti-phage defense system: Gabija	DefenseFinder
Lamassu-Cap4_nuclease	DEFENSE	DefenseFinder		Anti-phage defense system: Lamassu-Cap4_nuclease	DefenseFinder
PD-Lambda-1	DEFENSE	DefenseFinder		Anti-phage defense system: PD-Lambda-1	DefenseFinder
Pycsar	DEFENSE	DefenseFinder		Anti-phage defense system: Pycsar	DefenseFinder
RM_Type_I	DEFENSE	DefenseFinder		Anti-phage defense system: RM_Type_I	DefenseFinder
RM_Type_II	DEFENSE	DefenseFinder		Anti-phage defense system: RM_Type_II	DefenseFinder
RM_Type_IV	DEFENSE	DefenseFinder		Anti-phage defense system: RM_Type_IV	DefenseFinder
Retron_III	DEFENSE	DefenseFinder		Anti-phage defense system: Retron_III	DefenseFinder
RloC	DEFENSE	DefenseFinder		Anti-phage defense system: RloC	DefenseFinder
RosmerTA	DEFENSE	DefenseFinder		Anti-phage defense system: RosmerTA	DefenseFinder
ShosTA	DEFENSE	DefenseFinder		Anti-phage defense system: ShosTA	DefenseFinder
SoFic	DEFENSE	DefenseFinder		Anti-phage defense system: SoFic	DefenseFinder
Stk2	DEFENSE	DefenseFinder		Anti-phage defense system: Stk2	DefenseFinder
aac(6')-Ie/aph(2")-Ia	AMR	AMINOGLYCOSIDE	AMIKACIN/GENTAMICIN/ KANAMYCIN/TOBRAMYCIN	bifunctional aminoglycoside N-acetyltransferase AAC(6')-Ie/aminoglycoside O-phosphotransferase APH(2")-Ia	AMRFinderPlus
aadD1	AMR	AMINOGLYCOSIDE	KANAMYCIN/TOBRAMYCIN	aminoglycoside O-nucleotidyltransferase ANT(4')-Ia	AMRFinderPlus
adsA	VIRULENCE	VFDB		(adsA) Adenosine synthase A [AdsA (VF0422) - Immune modulation (VFC0258)] [Staphylococcus aureus subsp. aureus MW2]	VFDB (abricate)
ant(6)-Ia	AMR	AMINOGLYCOSIDE	STREPTOMYCIN	aminoglycoside nucleotidyltransferase ANT(6)-Ia	AMRFinderPlus
ant(9)-Ia	AMR	AMINOGLYCOSIDE	SPECTINOMYCIN	aminoglycoside nucleotidyltransferase ANT(9)-Ia	AMRFinderPlus
aph(3')-IIIa	AMR	AMINOGLYCOSIDE	AMIKACIN/KANAMYCIN	aminoglycoside O-phosphotransferase APH(3')-IIIa	AMRFinderPlus
apmA	AMR	AMINOGLYCOSIDE	APRAMYCIN	aminocyclitol acetyltransferase ApmA	AMRFinderPlus
arsB	STRESS	ARSENIC	ARSENITE	arsenite efflux transporter membrane subunit ArsB	AMRFinderPlus
arsC	STRESS	ARSENIC	ARSENATE	thioredoxin-dependent arsenate reductase	AMRFinderPlus
arsR	STRESS	ARSENIC	ARSENIC	As(III)-sensing metalloregulatory transcriptional repressor ArsR	AMRFinderPlus
aur	VIRULENCE	NA	NA	zinc metalloproteinase aureolysin	AMRFinderPlus
blaI	AMR	BETA-LACTAM	BETA-LACTAM	penicillinase repressor BlaI	AMRFinderPlus
blaPC1	AMR	BETA-LACTAM	BETA-LACTAM	BlaZ family penicillin-hydrolyzing class A beta-lactamase PC1	AMRFinderPlus
blaR1	AMR	BETA-LACTAM	BETA-LACTAM	beta-lactam sensor/signal transducer BlaR1	AMRFinderPlus
blaZ	AMR	BETA-LACTAM	BETA-LACTAM	penicillin-hydrolyzing class A beta-lactamase BlaZ	AMRFinderPlus
bleO	AMR	BLEOMYCIN	BLEOMYCIN	bleomycin binding protein	AMRFinderPlus
cadC	STRESS	CADMIUM/LEAD/ZINC	CADMIUM/LEAD/ZINC	Cd(II)/Pb(II)/Zn(II)-sensing metalloregulatory transcriptional repressor CadC	AMRFinderPlus
cadD	STRESS	CADMIUM	CADMIUM	cadmium resistance transporter CadD	AMRFinderPlus

(continued)

Gene or System	Type	Class	Subclass	Product or Function	Database source
cap8B	VIRULENCE	VFDB		(cap8B) type 8 capsular polysaccharide synthesis protein Cap8B [Capsule (VF0003) - Immune modulation (VFC0258)] [Staphylococcus aureus subsp. aureus MW2]	VFDB (abricate)
cap8C	VIRULENCE	VFDB		(cap8C) type 8 capsular polysaccharide synthesis protein Cap8C [Capsule (VF0003) - Immune modulation (VFC0258)] [Staphylococcus aureus subsp. aureus MW2]	VFDB (abricate)
cap8D	VIRULENCE	VFDB		(cap8D) type 8 capsular polysaccharide synthesis protein Cap8D [Capsule (VF0003) - Immune modulation (VFC0258)] [Staphylococcus aureus subsp. aureus MW2]	VFDB (abricate)
cap8E	VIRULENCE	VFDB		(cap8E) type 8 capsular polysaccharide synthesis protein Cap8E [Capsule (VF0003) - Immune modulation (VFC0258)] [Staphylococcus aureus subsp. aureus MW2]	VFDB (abricate)
cap8F	VIRULENCE	VFDB		(cap8F) type 8 capsular polysaccharide synthesis protein Cap8F [Capsule (VF0003) - Immune modulation (VFC0258)] [Staphylococcus aureus subsp. aureus MW2]	VFDB (abricate)
cap8G	VIRULENCE	VFDB		(cap8G) type 8 capsular polysaccharide synthesis protein Cap8G [Capsule (VF0003) - Immune modulation (VFC0258)] [Staphylococcus aureus subsp. aureus MW2]	VFDB (abricate)
cap8H	VIRULENCE	VFDB		(cap8H) type 8 capsular polysaccharide synthesis protein Cap8H [Capsule (VF0003) - Immune modulation (VFC0258)] [Staphylococcus aureus subsp. aureus MW2]	VFDB (abricate)
cap8I	VIRULENCE	VFDB		(cap8I) type 8 capsular polysaccharide synthesis protein Cap8I [Capsule (VF0003) - Immune modulation (VFC0258)] [Staphylococcus aureus subsp. aureus MW2]	VFDB (abricate)
cap8J	VIRULENCE	VFDB		(cap8J) type 8 capsular polysaccharide synthesis protein Cap8J [Capsule (VF0003) - Immune modulation (VFC0258)] [Staphylococcus aureus subsp. aureus MW2]	VFDB (abricate)
cap8K	VIRULENCE	VFDB		(cap8K) type 8 capsular polysaccharide synthesis protein Cap8K [Capsule (VF0003) - Immune modulation (VFC0258)] [Staphylococcus aureus subsp. aureus MW2]	VFDB (abricate)
cap8L	VIRULENCE	VFDB		(cap8L) type 8 capsular polysaccharide synthesis protein Cap8L [Capsule (VF0003) - Immune modulation (VFC0258)] [Staphylococcus aureus subsp. aureus MW2]	VFDB (abricate)
cap8M	VIRULENCE	VFDB		(cap8M) type 8 capsular polysaccharide synthesis protein Cap8M [Capsule (VF0003) - Immune modulation (VFC0258)] [Staphylococcus aureus subsp. aureus MW2]	VFDB (abricate)
cap8O	VIRULENCE	VFDB		(cap8O) type 8 capsular polysaccharide synthesis protein Cap8O [Capsule (VF0003) - Immune modulation (VFC0258)] [Staphylococcus aureus subsp. aureus MW2]	VFDB (abricate)
cap8P	VIRULENCE	VFDB		(cap8P) type 8 capsular polysaccharide synthesis protein Cap8P [Capsule (VF0003) - Immune modulation (VFC0258)] [Staphylococcus aureus subsp. aureus MW2]	VFDB (abricate)
capA	VIRULENCE	VFDB		(capA) capsular polysaccharide type 5/8 biosynthesis protein CapA [Capsule (VF0003) - Immune modulation (VFC0258)] [Staphylococcus aureus subsp. aureus MW2]	VFDB (abricate)

(continued)

Gene or System	Type	Class	Subclass	Product or Function	Database source
capN	VIRULENCE	VFDB		(capN) capsular polysaccharide type 5/8 biosynthesis epimerase CapN [Capsule (VF0003) - Immune modulation (VFC0258)] [Staphylococcus aureus subsp. aureus MW2]	VFDB (abricate)
chp	VIRULENCE	VFDB		(chp) chemotaxis-inhibiting protein CHIPS [CHIPS (VF0424) - Immune modulation (VFC0258)] [Staphylococcus aureus subsp. aureus str. Newman]	VFDB (abricate)
clfA	VIRULENCE	VFDB		(clfA) Clumping factor A fibrinogen-binding protein [Clumping factor (VF0004) - Adherence (VFC0001)] [Staphylococcus aureus subsp. aureus MW2]	VFDB (abricate)
clfB	VIRULENCE	VFDB		(clfB) Clumping factor B adhesin [Clumping factor (VF0004) - Adherence (VFC0001)] [Staphylococcus aureus subsp. aureus MW2]	VFDB (abricate)
cna	VIRULENCE	NA	NA	collagen adhesin Cna	AMRFinderPlus
coa	VIRULENCE	VFDB		(coa) staphylocoagulase precursor [Staphylocoagulase (VF0421) - Exoenzyme (VFC0251)] [Staphylococcus aureus subsp. aureus MW2]	VFDB (abricate)
dfrB_A135T	AMR	TRIMETHOPRIM	TRIMETHOPRIM	Staphylococcus aureus trimethoprim resistant DfrB	AMRFinderPlus
dfrS1	AMR	TRIMETHOPRIM	TRIMETHOPRIM	trimethoprim-resistant dihydrofolate reductase DfrS1	AMRFinderPlus
eap/map	VIRULENCE	VFDB		(eap/map) extracellular adherence protein Eap/Map [Eap/Map (VF0016) - Adherence (VFC0001)] [Staphylococcus aureus subsp. aureus MW2]	VFDB (abricate)
ebp	VIRULENCE	VFDB		(ebp) cell surface elastin binding protein [EbpS (VF0008) - Adherence (VFC0001)] [Staphylococcus aureus subsp. aureus MW2]	VFDB (abricate)
ednB	VIRULENCE	NA	NA	epidermal cell differentiation inhibitor EdnB	AMRFinderPlus
erm(A)	AMR	LINCOSAMIDE/MACROLIDE	AZITHROMYCIN/ CLARITHROMYCIN/ CLINDAMYCIN/ERYTHROMYCIN/ TELITHROMYCIN	23S rRNA (adenine(2058)-N(6))-methyltransferase Erm(A)	AMRFinderPlus
erm(C)	AMR	LINCOSAMIDE/MACROLIDE/STREPTOGRAMIN	CLINDAMYCIN/ERYTHROMYCIN/ STREPTOGRAMIN B/TYLOSIN	23S rRNA (adenine(2058)-N(6))-methyltransferase Erm(C)	AMRFinderPlus
esaA	VIRULENCE	VFDB		(esaA) type VII secretion system protein EsaA [Type VII secretion system (VF0403) - Effector delivery system (VFC0086)] [Staphylococcus aureus subsp. aureus MW2]	VFDB (abricate)
esaB	VIRULENCE	VFDB		(esaB) type VII secretion system protein EsaB [Type VII secretion system (VF0403) - Effector delivery system (VFC0086)] [Staphylococcus aureus subsp. aureus MW2]	VFDB (abricate)
esaD	VIRULENCE	VFDB		(esaD) type VII secretion system secreted protein a nuclease toxin EsaD [Type VII secretion system (VF0403) - Effector delivery system (VFC0086)] [Staphylococcus aureus subsp. aureus MW2]	VFDB (abricate)
esaE	VIRULENCE	VFDB		(esaE) type VII secretion system chaperone protein [Type VII secretion system (VF0403) - Effector delivery system (VFC0086)] [Staphylococcus aureus subsp. aureus MW2]	VFDB (abricate)
esaG	VIRULENCE	VFDB		(esaG) TIGR01741 family protein [Type VII secretion system (VF0403) - Effector delivery system (VFC0086)] [Staphylococcus aureus subsp. aureus MW2]	VFDB (abricate)
essA	VIRULENCE	VFDB		(essA) type VII secretion system protein EssA monotopic membrane protein [Type VII secretion system (VF0403) - Effector delivery system (VFC0086)] [Staphylococcus aureus subsp. aureus MW2]	VFDB (abricate)
essB	VIRULENCE	VFDB		(essB) type VII secretion system protein EssB monotopic membrane protein [Type VII secretion system (VF0403) - Effector delivery system (VFC0086)] [Staphylococcus aureus subsp. aureus MW2]	VFDB (abricate)

(continued)

Gene or System	Type	Class	Subclass	Product or Function	Database source
essC	VIRULENCE	VFDB		(essC) type VII secretion system protein EssC FtsK/SpoIIIE family ATPase [Type VII secretion system (VF0403) - Effector delivery system (VFC0086)] [Staphylococcus aureus subsp. aureus MW2]	VFDB (abricate)
esxA	VIRULENCE	VFDB		(esxA) type VII secretion system secreted protein EsxA [Type VII secretion system (VF0403) - Effector delivery system (VFC0086)] [Staphylococcus aureus subsp. aureus MW2]	VFDB (abricate)
esxB	VIRULENCE	VFDB		(esxB) type VII secretion system secreted protein EsxB [Type VII secretion system (VF0403) - Effector delivery system (VFC0086)] [Staphylococcus aureus subsp. aureus MW2]	VFDB (abricate)
esxC	VIRULENCE	VFDB		(esxC) type VII secretion system secreted protein EsxC [Type VII secretion system (VF0403) - Effector delivery system (VFC0086)] [Staphylococcus aureus subsp. aureus MW2]	VFDB (abricate)
esxD	VIRULENCE	VFDB		(esxD) type VII secretion system secreted protein EsxD [Type VII secretion system (VF0403) - Effector delivery system (VFC0086)] [Staphylococcus aureus subsp. aureus MW2]	VFDB (abricate)
etd	VIRULENCE	VFDB		(etd) exfoliative toxin D [Exfoliative toxin (VF0009) - Exoenzyme (VFC0251)] [Staphylococcus aureus str. pt251]	VFDB (abricate)
ete	VIRULENCE	VFDB		(ete) exfoliative toxin E [Exfoliative toxin (VF0009) - Exoenzyme (VFC0251)] [Staphylococcus aureus str. O46]	VFDB (abricate)
fnbA	VIRULENCE	VFDB		(fnbA) fibronectin-binding protein A [FnBPs (VF0010) - Adherence (VFC0001)] [Staphylococcus aureus subsp. aureus MW2]	VFDB (abricate)
fnbB	VIRULENCE	VFDB		(fnbB) fibronectin-binding protein B [FnBPs (VF0010) - Adherence (VFC0001)] [Staphylococcus aureus subsp. aureus MW2]	VFDB (abricate)
fosB	AMR	FOSFOMYCIN	FOSFOMYCIN	FosB/FosD family fosfomycin resistance bacillithiol transferase	AMRFinderPlus
gcu233	DEFENSE	DefenseFinder		Anti-phage defense system: gcu233	DefenseFinder
geh	VIRULENCE	VFDB		(geh) glycerol ester hydrolase [Lipase (VF0012) - Exoenzyme (VFC0251)] [Staphylococcus aureus subsp. aureus MW2]	VFDB (abricate)
glpT_A100V	AMR	FOSFOMYCIN	FOSFOMYCIN	Staphylococcus aureus fosfomycin resistant GlpT	AMRFinderPlus
glpT_V213I	AMR	FOSFOMYCIN	FOSFOMYCIN	Staphylococcus aureus fosfomycin resistant GlpT	AMRFinderPlus
gyrA_S84L	AMR	QUINOLONE	QUINOLONE	Staphylococcus aureus quinolone resistant GyrA	AMRFinderPlus
harA	VIRULENCE	VFDB		(harA) haptoglobin-binding heme uptake protein HarA [Isd (VF0015) - Nutritional/Metabolic factor (VFC0272)] [Staphylococcus aureus subsp. aureus str. Newman]	VFDB (abricate)
hlb	VIRULENCE	VFDB		(hlb) beta-hemolysin [Beta-hemolysin (VF0002) - Exotoxin (VFC0235)] [Staphylococcus aureus subsp. aureus COL]	VFDB (abricate)
hld	VIRULENCE	NA	NA	delta-hemolysin	AMRFinderPlus
hlgA	VIRULENCE	NA	NA	bi-component gamma-hemolysin HlgAB subunit A	AMRFinderPlus
hlgB	VIRULENCE	NA	NA	bi-component gamma-hemolysin HlgAB/HlgCB subunit B	AMRFinderPlus
hlgC	VIRULENCE	NA	NA	bi-component gamma-hemolysin HlgCB subunit C	AMRFinderPlus
hly/hla	VIRULENCE	VFDB		(hly/hla) Alpha-Hemolysin precursor [Alpha-hemolysin (VF0001) - Exotoxin (VFC0235)] [Staphylococcus aureus subsp. aureus MW2]	VFDB (abricate)
hysA	VIRULENCE	VFDB		(hysA) hyaluronate lyase precursor [Hyaluronate lyase (VF0013) - Exoenzyme (VFC0251)] [Staphylococcus aureus subsp. aureus MW2]	VFDB (abricate)
icaA	VIRULENCE	VFDB		(icaA) N-acetylglucosaminyltransferase involved in polysaccharide intercellular adhesin(PIA) synthesis [Intercellular adhesion proteins (VF0014) - Biofilm (VFC0271)] [Staphylococcus aureus subsp. aure	VFDB (abricate)

(continued)

Gene or System	Type	Class	Subclass	Product or Function	Database source
icaB	VIRULENCE	VFDB		(icaB) N-deacetylase involved in polysaccharide intercellular adhesin(PIA) synthesis [Intercellular adhesion proteins (VF0014) - Biofilm (VFC0271)] [Staphylococcus aureus subsp. aureus MW2]	VFDB (abricate)
icaC	VIRULENCE	NA	NA	polysaccharide intercellular adhesin biosynthesis/ export protein IcaC	AMRFinderPlus
icaD	VIRULENCE	VFDB		(icaD) intercellular adhesion protein D involved in polysaccharide intercellular adhesin(PIA) synthesis [Intercellular adhesion proteins (VF0014) - Biofilm (VFC0271)] [Staphylococcus aureus subsp. aur	VFDB (abricate)
icaR	VIRULENCE	VFDB		(icaR) ica operon transcriptional regulator IcaR [Intercellular adhesion proteins (VF0014) - Biofilm (VFC0271)] [Staphylococcus aureus subsp. aureus MW2]	VFDB (abricate)
isdA	VIRULENCE	VFDB		(isdA) iron-regulated surface determinant protein A [Isd (VF0015) - Nutritional/Metabolic factor (VFC0272)] [Staphylococcus aureus subsp. aureus str. Newman]	VFDB (abricate)
isdB	VIRULENCE	VFDB		(isdB) iron-regulated surface determinant protein B haemoglobin receptor [Isd (VF0015) - Nutritional/ Metabolic factor (VFC0272)] [Staphylococcus aureus subsp. aureus str. Newman]	VFDB (abricate)
isdC	VIRULENCE	VFDB		(isdC) iron-regulated surface determinant protein C [Isd (VF0015) - Nutritional/Metabolic factor (VFC0272)] [Staphylococcus aureus subsp. aureus str. Newman]	VFDB (abricate)
isdD	VIRULENCE	VFDB		(isdD) iron-regulated surface determinant protein D [Isd (VF0015) - Nutritional/Metabolic factor (VFC0272)] [Staphylococcus aureus subsp. aureus str. Newman]	VFDB (abricate)
isdE	VIRULENCE	VFDB		(isdE) iron-regulated surface determinant protein E [Isd (VF0015) - Nutritional/Metabolic factor (VFC0272)] [Staphylococcus aureus subsp. aureus str. Newman]	VFDB (abricate)
isdF	VIRULENCE	VFDB		(isdF) iron-regulated surface determinant protein F ATP-binding-cassette-type transmembrane transporter [Isd (VF0015) - Nutritional/Metabolic factor (VFC0272)] [Staphylococcus aureus subsp. aureus str	VFDB (abricate)
isdG	VIRULENCE	VFDB		(isdG) iron-regulated surface determinant protein G [Isd (VF0015) - Nutritional/Metabolic factor (VFC0272)] [Staphylococcus aureus subsp. aureus str. Newman]	VFDB (abricate)
isdI	VIRULENCE	VFDB		(isdI) staphylobilin-forming heme oxygenase IsdI [Isd (VF0015) - Nutritional/Metabolic factor (VFC0272)] [Staphylococcus aureus subsp. aureus str. Newman]	VFDB (abricate)
lip	VIRULENCE	VFDB		(lip) triacylglycerol lipase precursor [Lipase (VF0012) - Exoenzyme (VFC0251)] [Staphylococcus aureus subsp. aureus MW2]	VFDB (abricate)
lmrS	STRESS	MACROLIDE/PHENICOL	CHLORAMPHENICOL/ ERYTHROMYCIN	multidrug efflux MFS transporter LmrS	AMRFinderPlus
lnu(A)	AMR	LINCOSAMIDE	LINCOSAMIDE	lincosamide nucleotidyltransferase Lnu(A)	AMRFinderPlus
lukD	VIRULENCE	NA	NA	bi-component leukocidin LukED subunit D	AMRFinderPlus
lukE	VIRULENCE	NA	NA	bi-component leukocidin LukED subunit E	AMRFinderPlus
lukF-PV	VIRULENCE	NA	NA	Panton-Valentine bi-component leukocidin subunit F	AMRFinderPlus
lukS-PV	VIRULENCE	NA	NA	Panton-Valentine bi-component leukocidin subunit S	AMRFinderPlus
mco	STRESS	COPPER	COPPER	multi-copper oxidase Mco	AMRFinderPlus
mecA	AMR	BETA-LACTAM	METHICILLIN	PBP2a family beta-lactam-resistant peptidoglycan transpeptidase MecA	AMRFinderPlus
mecI	AMR	BETA-LACTAM	METHICILLIN	mecA-type methicillin resistance repressor MecI	AMRFinderPlus
mecR1	AMR	BETA-LACTAM	METHICILLIN	beta-lactam sensor/signal transducer MecR1	AMRFinderPlus
mepA	AMR	TETRACYCLINE	TIGECYCLINE	multidrug efflux MATE transporter MepA	AMRFinderPlus
merA	STRESS	MERCURY	MERCURY	mercury(II) reductase	AMRFinderPlus
merB	STRESS	MERCURY	ORGANOMERCURY	organomercurial lyase MerB	AMRFinderPlus

(continued)

Gene or System	Type	Class	Subclass	Product or Function	Database source
merT	STRESS	MERCURY	MERCURY	mercuric transport protein MerT	AMRFinderPlus
mph(C)	AMR	MACROLIDE	ERYTHROMYCIN/SPIRAMYCIN/ TELITHROMYCIN	Mph(C) family macrolide 2'-phosphotransferase	AMRFinderPlus
msr(A)	AMR	MACROLIDE/STREPTOGRAMIN	ERYTHROMYCIN/ STREPTOGRAMIN B	ABC-F type ribosomal protection protein Msr(A)	AMRFinderPlus
mupA	AMR	MUPIROCIN	MUPIROCIN	mupirocin-resistant isoleucine–tRNA ligase MupA	AMRFinderPlus
murA_D278E	AMR	FOSFOMYCIN	FOSFOMYCIN	Staphylococcus aureus fosfomycin resistant MurA	AMRFinderPlus
murA_E291D	AMR	FOSFOMYCIN	FOSFOMYCIN	Staphylococcus aureus fosfomycin resistant MurA	AMRFinderPlus
murA_G257D	AMR	FOSFOMYCIN	FOSFOMYCIN	Staphylococcus aureus fosfomycin resistant MurA	AMRFinderPlus
murA_T396N	AMR	FOSFOMYCIN	FOSFOMYCIN	Staphylococcus aureus fosfomycin resistant MurA	AMRFinderPlus
parC_S80F	AMR	QUINOLONE	QUINOLONE	Staphylococcus aureus quinolone resistant ParC	AMRFinderPlus
parC_S80Y	AMR	QUINOLONE	QUINOLONE	Staphylococcus aureus quinolone resistant ParC	AMRFinderPlus
parE_P585S	AMR	QUINOLONE	QUINOLONE	Staphylococcus aureus quinolone resistant ParE	AMRFinderPlus
qacA	STRESS	QUATERNARY AMMONIUM	QUATERNARY AMMONIUM	quaternary ammonium compound efflux MFS transporter QacA	AMRFinderPlus
qacC	STRESS	QUATERNARY AMMONIUM	QUATERNARY AMMONIUM	quaternary ammonium compound efflux SMR transporter QacC	AMRFinderPlus
qacR	STRESS	QUATERNARY AMMONIUM	QUATERNARY AMMONIUM	multidrug-binding transcriptional regulator QacR	AMRFinderPlus
sak	VIRULENCE	NA	NA	staphylokinase	AMRFinderPlus
sasG	VIRULENCE	NA	NA	LPXTG-anchored surface protein SasG	AMRFinderPlus
sat4	AMR	STREPTOTHRICIN	STREPTOTHRICIN	streptothricin N-acetyltransferase Sat4	AMRFinderPlus
sbi	VIRULENCE	VFDB		(sbi) IgG-binding protein SBI [Sbi (VF0423) - Immune modulation (VFC0258)] [Staphylococcus aureus subsp. aureus MW2]	VFDB (abricate)
scn	VIRULENCE	NA	NA	complement inhibitor SCIN-A	AMRFinderPlus
sdrC	VIRULENCE	VFDB		(sdrC) Ser-Asp rich fibrinogen-binding bone sialoprotein-binding protein [SDr (VF0019) - Adherence (VFC0001)] [Staphylococcus aureus subsp. aureus MW2]	VFDB (abricate)
sdrD	VIRULENCE	VFDB		(sdrD) Ser-Asp rich fibrinogen-binding bone sialoprotein-binding protein [SDr (VF0019) - Adherence (VFC0001)] [Staphylococcus aureus subsp. aureus MW2]	VFDB (abricate)
sdrE	VIRULENCE	VFDB		(sdrE) Ser-Asp rich fibrinogen-binding bone sialoprotein-binding protein [SDr (VF0019) - Adherence (VFC0001)] [Staphylococcus aureus subsp. aureus MW2]	VFDB (abricate)
sea	VIRULENCE	VFDB		(sea) staphylococcal enterotoxin A precursor [SE (VF0020) - Exotoxin (VFC0235)] [Staphylococcus aureus subsp. aureus MW2]	VFDB (abricate)
seb	VIRULENCE	NA	NA	staphylococcal enterotoxin type B	AMRFinderPlus
sed	VIRULENCE	NA	NA	staphylococcal enterotoxin type D	AMRFinderPlus
sei	VIRULENCE	NA	NA	staphylococcal enterotoxin type I	AMRFinderPlus
sej	VIRULENCE	NA	NA	staphylococcal enterotoxin type J	AMRFinderPlus
sek	VIRULENCE	NA	NA	staphylococcal enterotoxin type K	AMRFinderPlus
sel26	VIRULENCE	NA	NA	staphylococcal enterotoxin type 26	AMRFinderPlus
selX	VIRULENCE	NA	NA	staphylococcal enterotoxin-like toxin X	AMRFinderPlus
selk	VIRULENCE	VFDB		(selk) staphylococcal enterotoxin K precursor [SE (VF0020) - Exotoxin (VFC0235)] [Staphylococcus aureus subsp. aureus MW2]	VFDB (abricate)
selq	VIRULENCE	VFDB		(selq) staphylococcal enterotoxin G precursor [SE (VF0020) - Exotoxin (VFC0235)] [Staphylococcus aureus subsp. aureus MW2]	VFDB (abricate)
sem	VIRULENCE	NA	NA	staphylococcal enterotoxin type M	AMRFinderPlus
sen	VIRULENCE	NA	NA	staphylococcal enterotoxin type N	AMRFinderPlus
seo	VIRULENCE	NA	NA	staphylococcal enterotoxin type O	AMRFinderPlus
sep	VIRULENCE	NA	NA	staphylococcal enterotoxin type P	AMRFinderPlus
seq	VIRULENCE	NA	NA	staphylococcal enterotoxin type Q	AMRFinderPlus
ser	VIRULENCE	NA	NA	staphylococcal enterotoxin type R	AMRFinderPlus
set16	VIRULENCE	VFDB		(set16) superantigen-like protein SSL1 [SSLs (VF0990) - Exotoxin (VFC0235)] [Staphylococcus aureus subsp. aureus MW2]	VFDB (abricate)

(continued)

Gene or System	Type	Class	Subclass	Product or Function	Database source
set17	VIRULENCE	VFDB		(set17) superantigen-like protein SSL2 [SSLs (VF0990) - Exotoxin (VFC0235)] [Staphylococcus aureus subsp. aureus MW2]	VFDB (abricate)
set18	VIRULENCE	VFDB		(set18) superantigen-like protein SSL3 [SSLs (VF0990) - Exotoxin (VFC0235)] [Staphylococcus aureus subsp. aureus MW2]	VFDB (abricate)
set19	VIRULENCE	VFDB		(set19) superantigen-like protein SSL4 [SSLs (VF0990) - Exotoxin (VFC0235)] [Staphylococcus aureus subsp. aureus MW2]	VFDB (abricate)
set20	VIRULENCE	VFDB		(set20) superantigen-like protein SSL5 [SSLs (VF0990) - Exotoxin (VFC0235)] [Staphylococcus aureus subsp. aureus MW2]	VFDB (abricate)
set21	VIRULENCE	VFDB		(set21) superantigen-like protein SSL6 [SSLs (VF0990) - Exotoxin (VFC0235)] [Staphylococcus aureus subsp. aureus MW2]	VFDB (abricate)
set22	VIRULENCE	VFDB		(set22) superantigen-like protein SSL7 [SSLs (VF0990) - Exotoxin (VFC0235)] [Staphylococcus aureus subsp. aureus MW2]	VFDB (abricate)
set23	VIRULENCE	VFDB		(set23) superantigen-like protein SSL8 [SSLs (VF0990) - Exotoxin (VFC0235)] [Staphylococcus aureus subsp. aureus MW2]	VFDB (abricate)
set24	VIRULENCE	VFDB		(set24) superantigen-like protein SSL9 [SSLs (VF0990) - Exotoxin (VFC0235)] [Staphylococcus aureus subsp. aureus MW2]	VFDB (abricate)
set25	VIRULENCE	VFDB		(set25) superantigen-like protein SSL10 [SSLs (VF0990) - Exotoxin (VFC0235)] [Staphylococcus aureus subsp. aureus MW2]	VFDB (abricate)
set26	VIRULENCE	VFDB		(set26) superantigen-like protein SSL11 [SSLs (VF0990) - Exotoxin (VFC0235)] [Staphylococcus aureus subsp. aureus MW2]	VFDB (abricate)
seu	VIRULENCE	NA	NA	staphylococcal enterotoxin type U	AMRFinderPlus
spa	VIRULENCE	VFDB		(spa) Immunoglobulin G binding protein A precursor [SpA (VF0017) - Exotoxin (VFC0235)] [Staphylococcus aureus subsp. aureus MW2]	VFDB (abricate)
splA	VIRULENCE	NA	NA	serine protease SplA	AMRFinderPlus
splB	VIRULENCE	NA	NA	serine protease SplB	AMRFinderPlus
splE	VIRULENCE	NA	NA	serine protease SplE	AMRFinderPlus
srtB	VIRULENCE	VFDB		(srtB) NPQTN specific sortase B [Isd (VF0015) - Nutritional/Metabolic factor (VFC0272)] [Staphylococcus aureus subsp. aureus str. Newman]	VFDB (abricate)
sspA	VIRULENCE	VFDB		(sspA) serine protease; V8 protease; glutamyl endopeptidase [V8 protease (VF0023) - Exoenzyme (VFC0251)] [Staphylococcus aureus subsp. aureus MW2]	VFDB (abricate)
sspB	VIRULENCE	VFDB		(sspB) staphopain cysteine proteinase SspB [Staphopain (VF0006) - Exoenzyme (VFC0251)] [Staphylococcus aureus subsp. aureus MW2]	VFDB (abricate)
sspC	VIRULENCE	VFDB		(sspC) Staphostatin B [Staphopain (VF0006) - Exoenzyme (VFC0251)] [Staphylococcus aureus subsp. aureus MW2]	VFDB (abricate)
tet(38)	AMR	TETRACYCLINE	TETRACYCLINE	tetracycline efflux MFS transporter Tet(38)	AMRFinderPlus
tet(K)	AMR	TETRACYCLINE	TETRACYCLINE	tetracycline efflux MFS transporter Tet(K)	AMRFinderPlus
tsst-1	VIRULENCE	VFDB		(tsst-1) toxic shock syndrome toxin-1 [TSST-1 (VF0022) - Exotoxin (VFC0235)] [Staphylococcus aureus subsp. aureus N315]	VFDB (abricate)
tst	VIRULENCE	NA	NA	toxic shock syndrome toxin TSST-1	AMRFinderPlus
vWbp	VIRULENCE	VFDB		(vWbp) secreted von Willebrand factor-binding protein precursor [VWbp (VF0420) - Exoenzyme (VFC0251)] [Staphylococcus aureus subsp. aureus str. Newman]	VFDB (abricate)

Table S21. Genotype–phenotype concordance for antimicrobial resistance

WGS-based resistance predictions were compared with phenotypic MIC results (CLSI 2024 breakpoints) for 10 antimicrobial agents across 26 *S. aureus* isolates. Genotypic resistance was defined by established resistance determinants detected by AMRFinderPlus, CARD, and ResFinder.

Table S21. Genotype–phenotype concordance for antimicrobial resistance in 26 *S. aureus* isolates.

		Concordance								
Antibiotic	n	TP	TN	FP	FN	Sens	Spec	PPV	NPV	CA
Penicillin	26	22	2	1	1	96	67	96	67	92
Oxacillin	26	11	12	2	1	92	86	85	92	88
Erythromycin	26	12	10	2	2	86	83	86	83	85
Clindamycin	26	13	12	1	0	**100**	92	93	**100**	96
Ciprofloxacin	21	10	5	4	2	83	56	71	71	71
Tetracycline	26	2	20	3	1	67	87	40	95	85
TMP-SMX	26	0	25	0	1	0	**100**	—	96	96
Linezolid	26	0	26	0	0	—	**100**	—	**100**	**100**
Daptomycin	25	0	25	0	0	—	**100**	—	**100**	**100**
Vancomycin	26	0	26	0	0	—	**100**	—	**100**	**100**
**Overall**	**254**	**70**	**163**	**13**	**8**	**91**	**93**	**84**	**95**	**92**

TP, true positive; TN, true negative; FP, false positive; FN, false negative. Sens, sensitivity (%); Spec, specificity (%); PPV, positive predictive value (%); NPV, negative predictive value (%); CA, categorical agreement (%). CLSI 2024 breakpoints; intermediate classified as non-susceptible. Gene–antibiotic mapping: *blaZ*→PEN; *mecA/mecC*→OXA; *ermA/ermC/msrA/mphC*→ERY; *ermA/ermC*→CLI; QRDR mutations→CIP; *tetK/tetM*→TET; *dfrG/dfrA/dfrK*→SXT; *vanA/vanB*→VAN; *cfr/optrA*→LZD. Notable discordances: SA020 BORSA (OXA FN); SA015/SA027 heteroresistance (OXA FP); SA018 TMP-SMX non-susceptibility without detected acquired *dfr* determinants (SXT FN). VAN, LZD, and DAP achieved 100% concordance.

Table S22. De novo assembly statistics for 26 *S. aureus* isolates

Assemblies were generated using SPAdes v4.2.0 in standard paired-end mode with the `-careful` and `-only-assembler` options for Illumina paired-end reads. Contigs <500 bp were filtered. Expected genome size for *S. aureus*: 2.7–3.1 Mb; GC content: 32–34%. One isolate was excluded prior to assembly due to insufficient reads (460 total). Genome accessions are whole-genome shotgun (WGS) master records deposited in NCBI GenBank under BioProject PRJNA1437481.

Sample	BioSample	GenBank	Size (bp)	Contigs	N50 (bp)	GC (%)	Largest (bp)	QC	Notes
SA001	SAMN56511660	JBWDTT000000000	2,820,844	355	14,339	32.72	49,326	WARN	Fragmented
SA002	SAMN56511661	JBWDTU000000000	2,832,715	37	154,097	32.77	418,779	PASS	
SA004	SAMN56511662	JBWDTV000000000	2,828,888	36	165,708	32.76	458,057	PASS	
SA005	SAMN56511663	JBWDTW000000000	2,811,466	32	224,045	32.70	497,599	PASS	
SA006	SAMN56511664	JBWDTX000000000	2,892,142	45	167,046	32.64	510,896	PASS	
SA007	SAMN56511665	JBWDTY000000000	2,735,228	46	103,181	32.63	345,602	PASS	
SA008	SAMN56511666	JBWDTZ000000000	2,740,479	43	115,577	32.68	280,621	PASS	
SA009	SAMN56511667	JBWDUA000000000	2,687,773	54	86,052	32.70	256,539	WARN	Small genome
SA010	SAMN56511668	JBWDUB000000000	2,888,886	44	212,920	32.69	345,409	PASS	
SA011	SAMN56511669	JBWDUC000000000	2,731,934	29	196,296	32.71	489,760	PASS	
SA012	SAMN56511670	JBWDUD000000000	2,866,312	31	189,110	32.66	569,177	PASS	
SA013	SAMN56511671	JBWDUE000000000	2,734,054	21	300,928	32.66	460,520	PASS	
SA014	SAMN56511672	JBWDUF000000000	2,803,328	34	222,180	32.71	606,554	PASS	
SA015	SAMN56511673	JBWDUG000000000	2,824,811	1,990	1,721	32.59	31,820	WARN	Fragmented
SA016	SAMN56511674	JBWDUH000000000	3,455,764	59	161,838	32.71	366,712	WARN	Large genome
SA017	SAMN56511675	JBWDUI000000000	2,710,208	40	132,086	32.67	272,380	PASS	
SA018	SAMN56511676	JBWDUJ000000000	2,938,928	48	209,899	32.61	378,606	PASS	
SA019	SAMN56511677	JBWDUK000000000	2,833,249	36	154,097	32.77	358,802	PASS	
SA020	SAMN56511678	JBWDUL000000000	2,793,853	30	304,793	32.64	469,492	PASS	
SA021	SAMN56511679	JBWDUM000000000	2,833,694	36	165,708	32.77	418,779	PASS	
SA022	SAMN56511680	JBWDUN000000000	2,764,214	30	206,827	32.68	416,768	PASS	
SA023	SAMN56511681	JBWDUO000000000	2,853,091	33	222,694	32.67	646,040	PASS	
SA024	SAMN56511682	JBWDUP000000000	2,827,987	37	165,708	32.76	418,779	PASS	
SA025	SAMN56511683	JBWDUQ000000000	2,846,586	58	136,516	32.72	403,630	PASS	
SA026	SAMN56511684	JBWDUR000000000	2,831,466	36	207,009	32.77	358,802	PASS	
SA027	SAMN56511685	JBWDUS000000000	2,844,729	37	280,390	32.61	485,240	PASS	

Notes: Size, total assembled genome length; Contigs, number of contigs ≥500 bp; N50, minimum contig length at which 50% of assembly is contained in contigs of this length or longer; GC (%), guanine-cytosine percentage; Largest, length of the longest contig. GenBank, WGS master accession (BioProject PRJNA1437481). Assemblies were evaluated against expected *S. aureus* genome parameters (size 2.7–3.1 Mb; maximum 200 contigs; minimum N50 of 50 kb; GC 32–34%). PASS, all metrics acceptable; WARN, one or more metrics outside recommended thresholds. WARN assemblies were retained only when independent species confirmation and contamination screening supported their validity.

Table S23. Virulence factor inventory for 26 *S. aureus* isolates

Virulence genes were detected by ABRicate screening against VFDB (≥80% identity, ≥80% coverage). Genes are organized by functional category. PVL-positive: detection of both *lukS-PV* and *lukF-PV*; "partial" indicates detection of *lukF-PV* alone. IEC calls in this table derive from ABRicate/VFDB screening only; the final prophage-informed IEC classification used in the main text is based on Table S15.

		**PVL**		**Enterotoxins**			**IEC**			**Adhesins**		**Biofilm**		**Cap.**
**Sample**	**Status**	*lukS*	*lukF*	*sea*	*seb*	*sel/sek*	*scn*	*chp*	*sak*	*fnbA*	*cna*	*icaA*	*icaD*	CP
MRSA isolates (n = 13)														
SA001	MRSA	−	+	+	−	−	+	+	+	+	−	+	+	8
SA002	MRSA	−	+	+	−	−	+	+	+	+	−	+	+	8
SA004	MRSA	−	+	+	−	−	+	+	+	+	−	+	+	8
SA005	MRSA	+	+	−	−	−	+	−	+	+	−	−	+	8
SA006	MRSA	+	+	−	−	−	+	+	+	+	+	+	+	8
SA014	MRSA	−	+	+	−	−	+	+	+	+	−	+	+	8
SA015	MRSA	−	+	+	−	−	+	+	+	+	−	−	+	8
SA018	MRSA	+	+	−	−	+	+	+	+	+	−	+	+	8
SA019	MRSA	−	+	+	−	−	+	+	+	+	−	+	+	8
SA021	MRSA	−	+	+	−	−	+	+	+	+	−	+	+	8
SA024	MRSA	−	+	+	−	−	+	+	+	+	−	+	+	8
SA026	MRSA	−	+	+	−	−	+	+	+	+	−	+	+	8
SA027	MRSA	+	+	−	−	+	+	+	+	−	−	+	+	8
MSSA isolates (n = 13)														
SA007	MSSA	+	+	−	−	−	+	−	+	+	+	+	+	8
SA008	MSSA	−	−	−	−	−	+	+	+	−	+	+	+	8
SA009	MSSA	−	+	−	−	−	+	+	−	+	−	+	+	8
SA010	MSSA	+	+	−	−	+	+	+	+	+	−	+	+	8
SA011	MSSA	−	+	−	−	−	+	−	+	+	+	+	+	8
SA012	MSSA	−	+	−	−	−	+	+	+	+	+	+	+	8
SA013	MSSA	−	+	+	−	−	+	−	+	+	−	+	+	8
SA016	MSSA	−	+	−	−	−	−	−	−	+	−	+	+	8
SA017	MSSA	−	−	−	−	−	−	−	−	−	+	+	+	8
SA020	MSSA	−	+	−	+	−	+	+	+	+	−	+	+	8
SA022	MSSA	−	+	+	−	−	+	−	+	+	+	+	+	8
SA023	MSSA	−	+	−	−	−	+	+	+	+	−	+	+	8
SA025	MSSA	−	−	−	−	−	+	−	+	−	+	+	+	8

Notes: PVL, Panton–Valentine leukocidin (*lukS-PV*/*lukF-PV*); PVL-positive defined as detection of both subunits. IEC, immune evasion cluster; complete IEC defined as *scn*+*chp*+*sak*. Additional virulence genes universally present in all 26 isolates (not shown): hemolysins (*hla*, *hlb*, *hld*, *hlgA*, *hlgB*, *hlgC*), adhesins (*clfA*, *clfB*, *sdrC*, *sdrD*, *sdrE*, *spa*), biofilm genes (*icaB*, *icaC*), and iron acquisition system (*isdA*–*isdG*). CP, capsular polysaccharide type. Full per-gene virulence matrix available as Supplementary Data.

Supplementary Figures

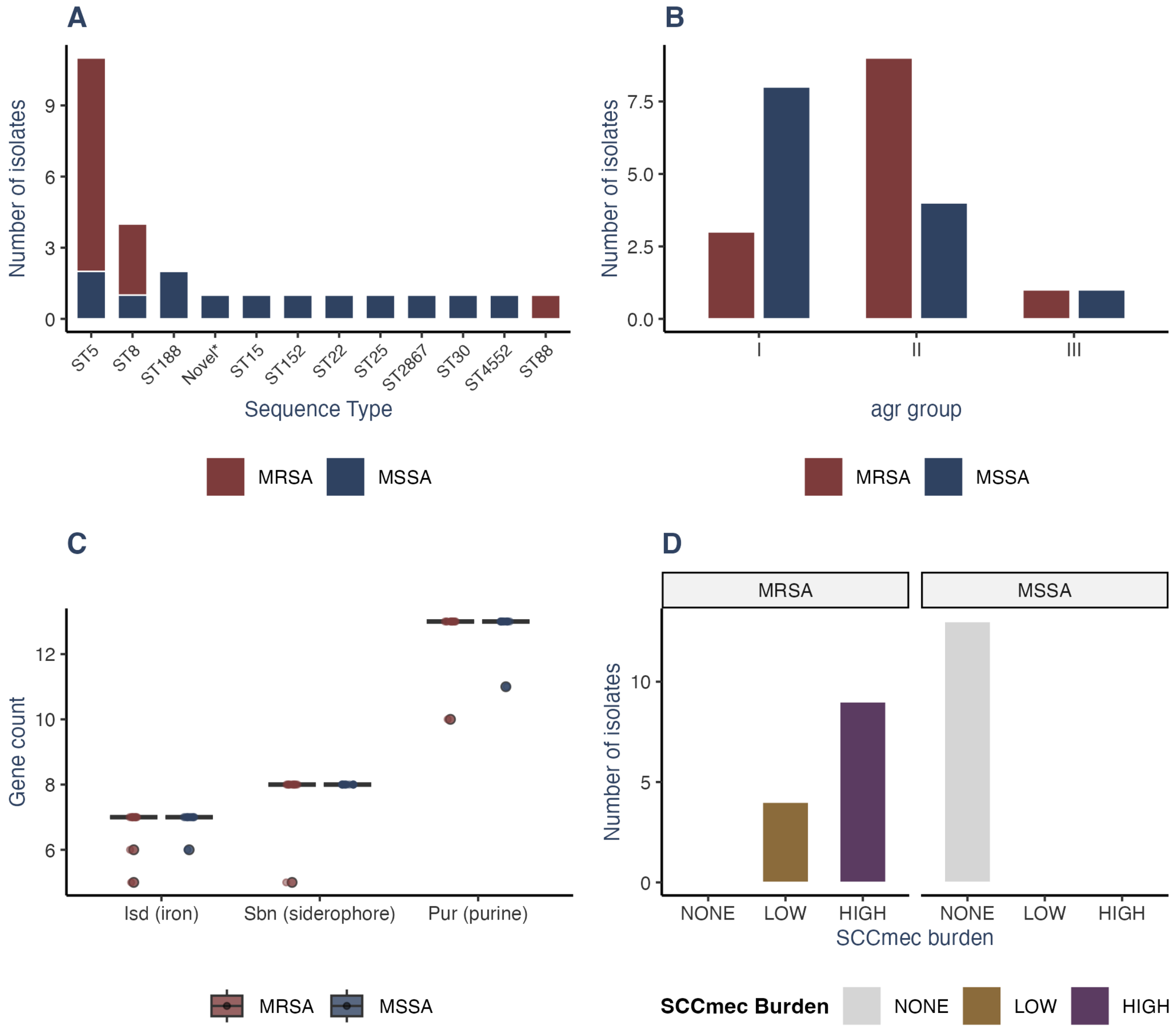

Figure S1. Comparative population structure and genomic marker analysis of MRSA (n = 13) and MSSA (n = 13). Panels: (A) sequence type distribution showing MRSA dominance by ST5 and polyclonal MSSA population; (B) *agr* group distribution; (C) iron-acquisition and purine-biosynthesis marker counts; (D) predicted SCC*mec*-associated burden categories.

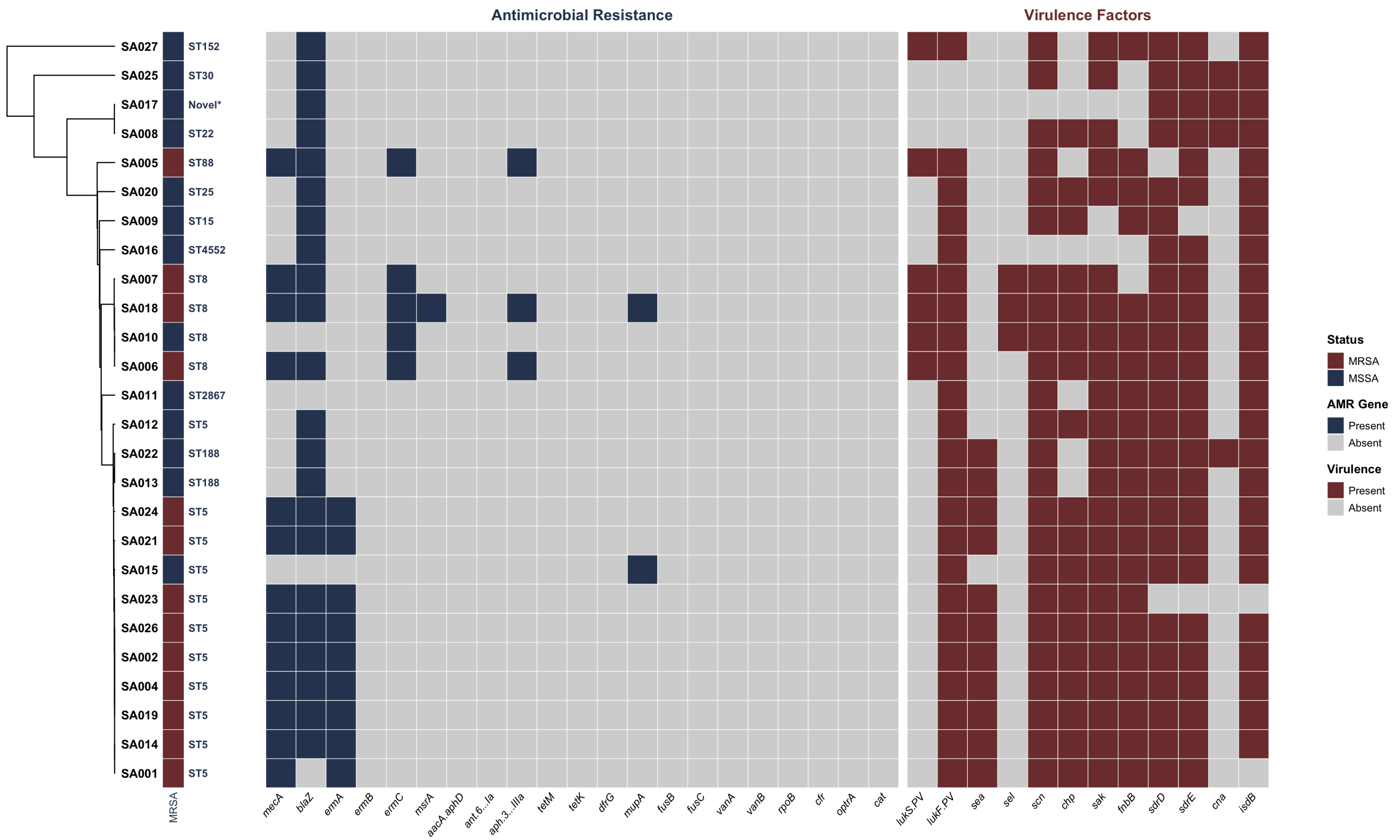

Figure S2. Combined antimicrobial-resistance and virulence heatmap showing the distribution of selected resistance determinants and key virulence-associated genes across 26 isolates aligned with the core-genome phylogeny. The left block summarizes antimicrobial-resistance markers, and the right block summarizes virulence-associated markers.

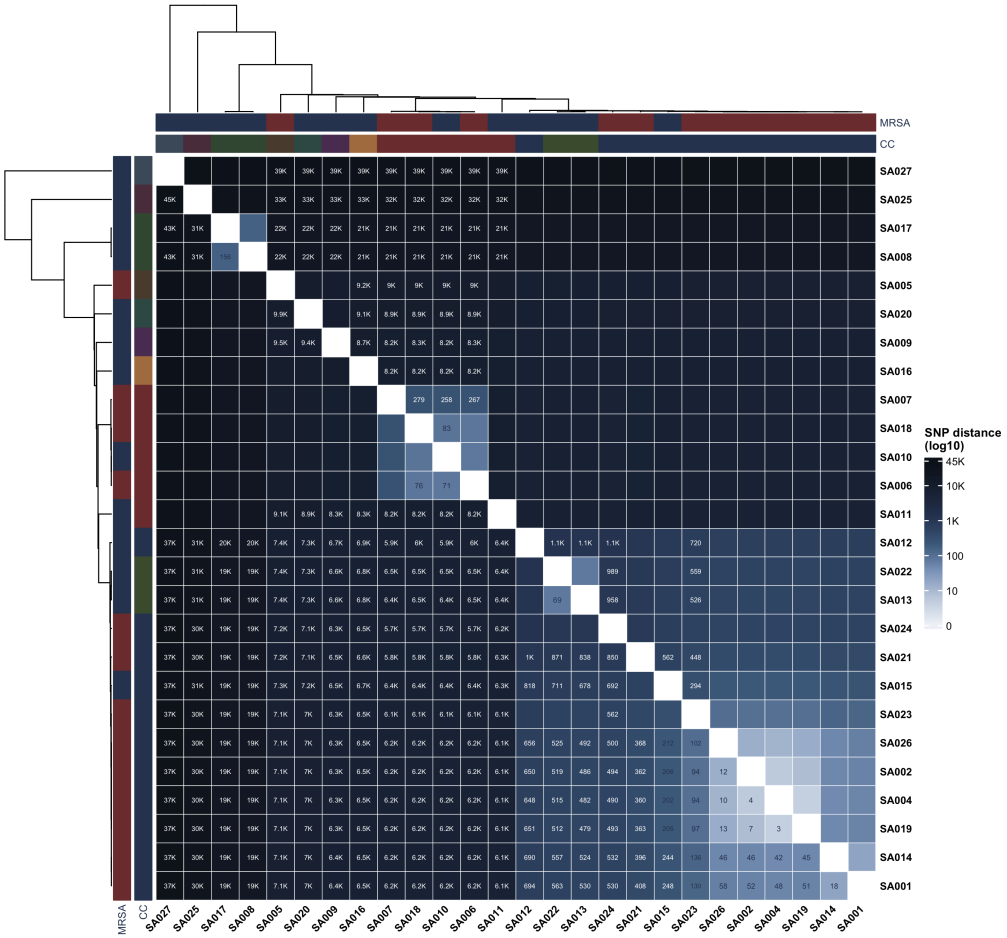

Figure S3. Pairwise core-genome SNP distance heatmap for all 26 isolates with hierarchical clustering. Color intensity reflects SNP distance (darker = fewer SNPs = closer relatedness). The CC5-MRSA-SCC*mec* II cluster shows characteristically low within-cluster distances (3–58 SNPs among the closest isolates), a pattern compatible with recent clonal spread.

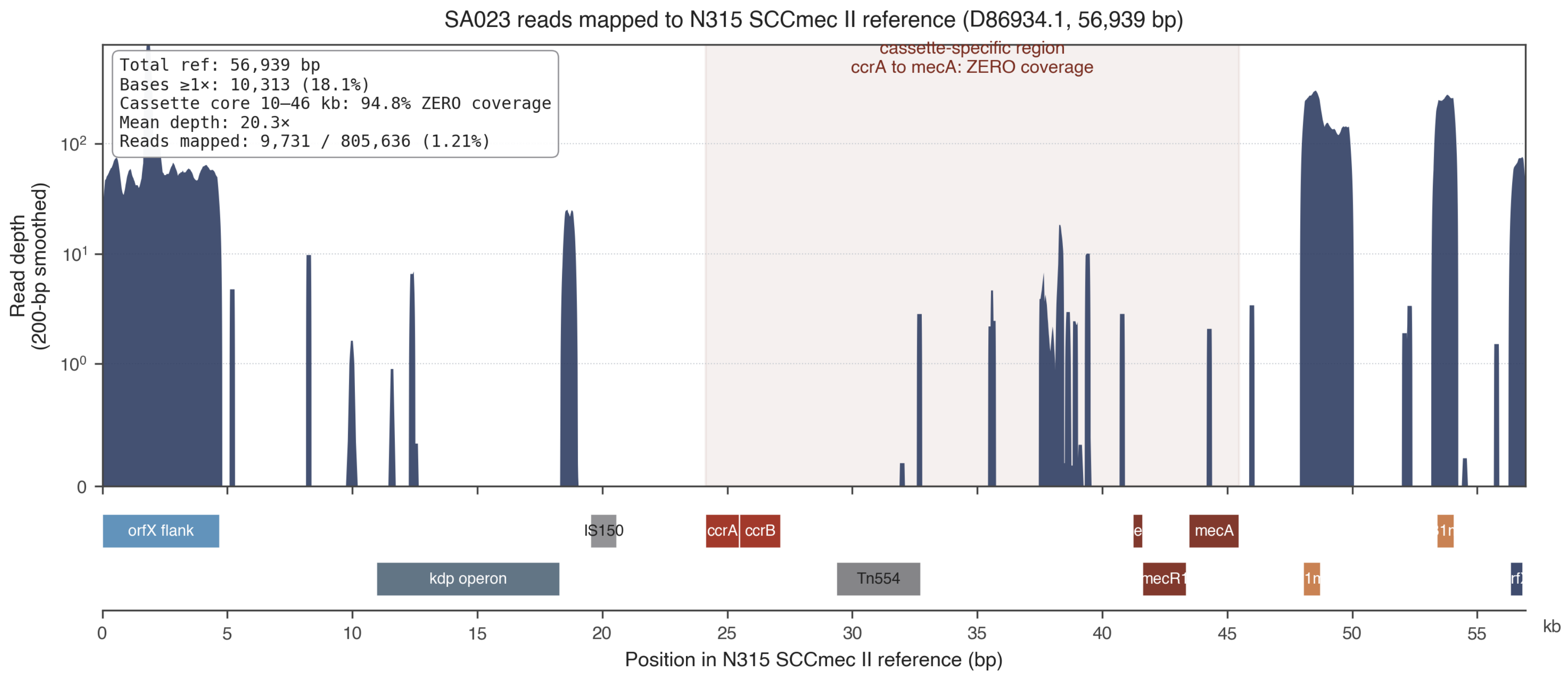

Figure S4. Quantitative read-mapping evidence for SCC*mec* II absence in SA023. Trimmed reads corresponding to final clinical ID SA023 were aligned with BWA-MEM v0.7.19 against the N315 SCC*mec* II reference (D86934.1, 56,939 bp). The coverage track (top, 200-bp smoothed, MSSA blue #344266) shows that 94.8% of the cassette-specific 36-kb region (*ccrA*–*mecA* core, light red shading) has zero coverage, confirming absence of cassette DNA in the SA023 read pool. Residual coverage falls at chromosomal flanks (positions 1–5 kb), the *kdp* auxiliary operon (10.9–18.3 kb; conserved in some staphylococci), and the IS431m elements at ∼48 kb and ∼54 kb (cross-mapping from the SA023 IS257-1 plasmid contig_23). Total mapped reads: 9,731 / 805,636 (1.21%); mean genome-wide depth 20.3×. Annotated features (bottom): *orfX* flank (chromosomal), *kdp* operon (slate blue #5A6E7F), IS150 (grey #8D8D91), *ccrA*/*ccrB* (red-orange #9D2E1F), Tn554 (#7E7E82), *mecI*/*mecR1*/*mecA* (MRSA red #7A2E21), IS431m left/right (warm orange #C67B4A), *orfX* chromosomal landmark (MSSA blue #344266).

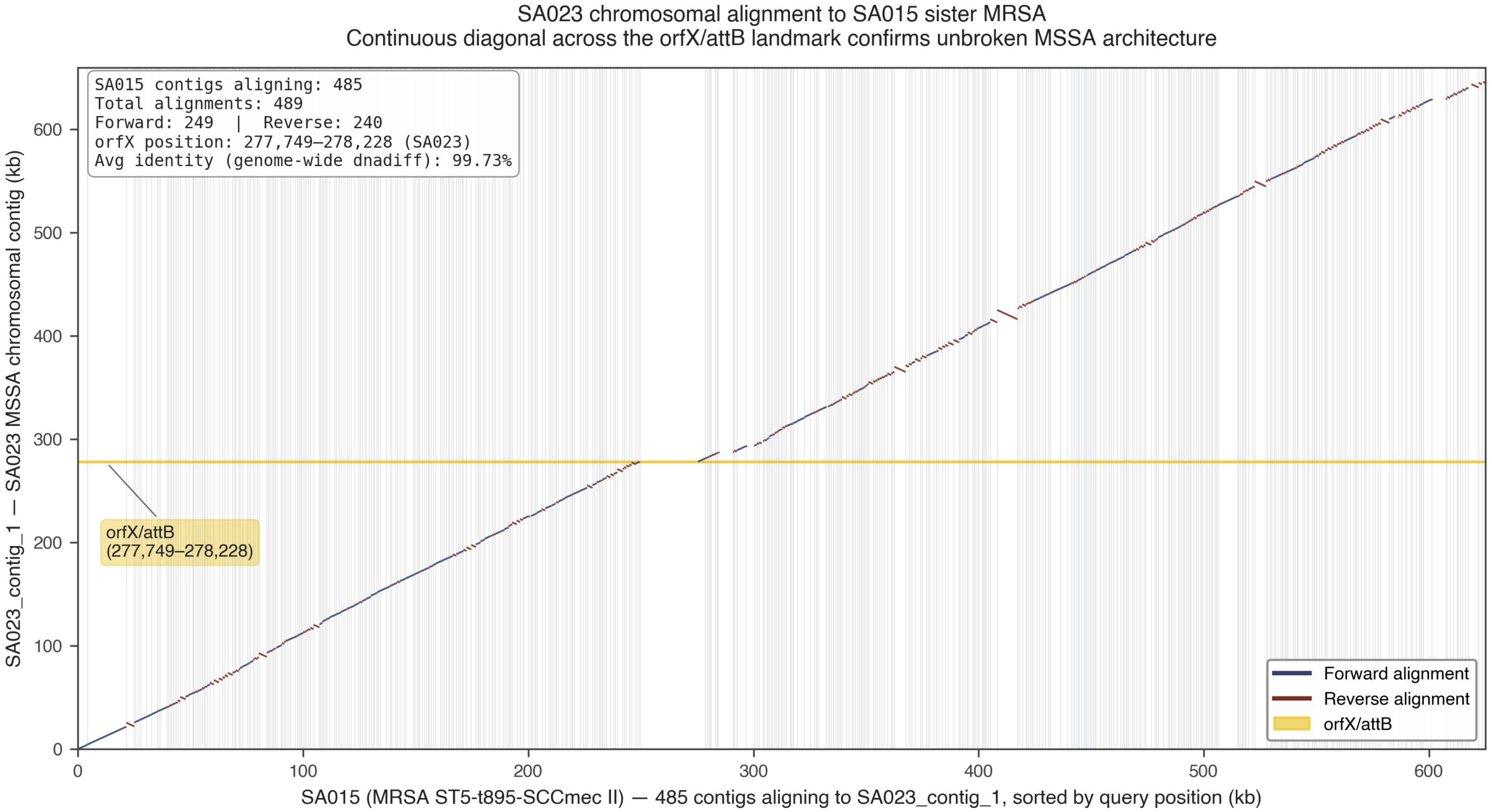

Figure S5. Focused chromosomal dotplot of SA023 versus its sister CC5-MRSA isolate SA015. The y-axis shows the SA023 chromosomal contig (SA023_contig_1, 646,040 bp); the x-axis shows the 485 SA015 contigs that align to it (≥200 bp), concatenated and sorted by their median query position. Forward 1-to-1 alignments (MSSA blue #344266) form an unbroken diagonal across the *orfX/attB* integration locus (gold band, position 277,749–278,228). Reverse alignments (MRSA red #7A2E21) are minor and scattered, as expected for two collinear chromosomal backbones. Genome-wide average identity (dnadiff) was 99.73%. The continuity of the diagonal at the integration locus is consistent with the canonical MSSA architecture being preserved in SA023 (no SCC*mec* insertion). Because SA015 is fragmented, this dotplot was used only to assess local chromosomal collinearity around the SA023 *orfX/attB* locus; SCC*mec* inference rests primarily on read-level absence of the cassette, intact *attB/orfX* architecture, and the complete *mec*-complex pattern in the nine CC5-MRSA sibling isolates.

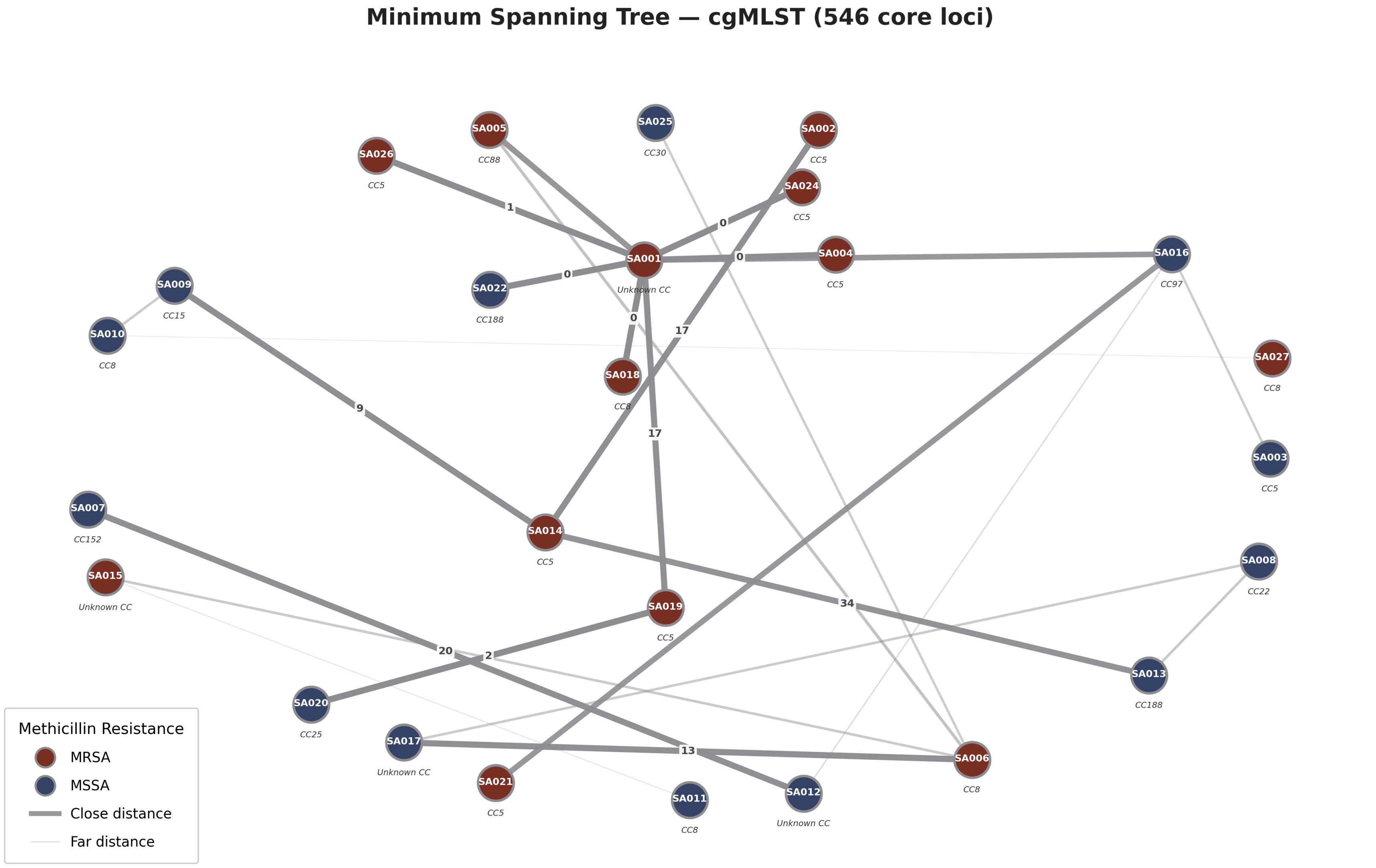

Figure S6. Minimum spanning tree based on cgMLST allelic distances (546 core loci) for 26 *S. aureus* isolates. Node colors indicate MRSA (red, #7A2E21) and MSSA (blue, #344266) status. Edge labels show the number of allelic differences. The CC5-MRSA-SCC*mec* II cluster shows characteristically low allelic distances, concordant with the SNP-based phylogeny.

Supplementary Data Files

The following supplementary data files are deposited in Zenodo (https://doi.org/10.5281/zenodo.19026167):

- **Supplementary Data S1.** Full per-gene virulence factor matrix (26 isolates × 94 genes) in tab-separated format.
- **Supplementary Data S2.** Complete 26×26 pairwise SNP distance matrix in tab-separated format.
- **Supplementary Data S3.** Core-genome alignment (FASTA) after recombination filtering with Gubbins.
- **Supplementary Data S4.** Newick tree files for the 26-taxon core-genome phylogeny (S4A) and the 40-taxon contextual phylogeny (S4B).
- **Supplementary Data S5.** iTOL phylogenetic tree and annotation files for reproducing Figures 1 and 2, including the recombination-filtered Newick tree and 10 dataset annotation files (methicillin resistance status, clonal complex, SCC*mec* type, *agr* group, PVL status, *spa* type, sequence type, syndromic category, clinical department, and in-hospital mortality).
