# Supplementary material for: Whole-genome sequencing of Staphylococcus aureus isolates after a decade-long decline in MRSA prevalence at a university referral hospital in Guadalajara, Mexico: clonal concentration in MRSA and greater genetic diversity in MSSA
Source: Microbiol Spectr. 2026 Jun 15;14(7):e00948-26. doi: 10.1128/spectrum.00948-26 (PMC13340018; doi:10.1128/spectrum.00948-26)
Supplement: Reviewer comments [file reviewer-comments.pdf]

AMERICAN SOCIETY FOR MICROBIOLOGY | Microbiology Spectrum

Whole-Genome Sequencing of *Staphylococcus aureus* Isolates after a Decade-Long Decline in MRSA Prevalence at a University Referral Hospital in Guadalajara, Mexico: Clonal Concentration in MRSA and Greater Genetic Diversity in MSSA

Jaime Andrade-Villanueva, Pedro Martínez-Ayala, Adolfo Gomez-Quiroz, Violeta Vera-Cuevas, Judith De Arcos-Jiménez, and Jaime Briseno-Ramirez

Corresponding Author(s): Jaime Briseno-Ramirez, Hospital Civil de Oriente

Review Timeline:

Submission Date:	March 18, 2026
Editorial Decision:	May 2, 2026
Revision Received:	May 7, 2026
Accepted:	May 9, 2026

Editor: Victor Gonzalez

Reviewer(s): Disclosure of reviewer identity is with reference to reviewer comments included in decision letter(s). The following individuals involved in review of your submission have agreed to reveal their identity: LUIS ESAU LOPEZ-JACOME (Reviewer #1)

Transaction Report:

(Note: With the exception of the correction of typographical or spelling errors that could be a source of ambiguity, letters and reports are not edited. The original formatting of letters and referee reports may not be reflected in this compilation.)

DOI: https://doi.org/10.1128/spectrum.00948-26

Re: Spectrum00948-26 (Whole-Genome Sequencing Reveals Clonal Vulnerability of Methicillin-Resistant extit{Staphylococcus aureus)

Dear Dr. Jaime Briseno-Ramirez:

Thank you for the privilege of reviewing your work. Below you will find my comments, instructions from the Spectrum editorial office, and the reviewer comments.

This manuscript presents valuable results on the analysis of MRSA and MSSA in a single hospital. However, several concerns regarding the extent of the conclusions should be addressed carefully. As editor, I suggest examining the following issues and modifying the corresponding sections accordingly:

1. The main claim of this manuscript is that the decline in MRSA prevalence within a hospital over a decade is likely due to molecular mechanisms involving the excision of the SCCmec cassette. This conclusion is supported by the phylogenomic analysis of a closely related MSSA (ST) nested with an MRSA clone. However, this observation is not sufficient to validate that this mechanism is associated with MRSA reduction or to conclude that MRSA is vulnerable. To make such statements, solid evidence should come from an ample population analysis of the frequencies of MRSA/MSSA. Therefore, in my opinion, the conclusion is overstated and needs to be modified, as does the title.

2. Species identification is commonly achieved by calculating the whole-genome average nucleotide identity (ANI), followed by core phylogeny. Kraken is best used in metagenomics, and its use with genomes is not reliable at the species level. Furthermore, Kraken does not provide identity percentages. It is unclear how this percentage was obtained (l- 153). We suggest using ANI or validating Kraken profiling as adequate for species identification.

3. Although fitness associated with the class of SCCmec cassette has been reported and pointed out in the manuscript, no experiment was performed to evaluate the fitness in the collection of strains used in the manuscript. I am not sure you can extrapolate the The genomic background of the strains, such as genome size and alleles, should also play a role in growth rates and overall fitness. I suggest reviewing the corresponding statements.

4. The section "Risk Factors for MRSA Infection" appears somewhat disconnected from the main purpose of the manuscript. I suggest moving it to the first section of the Results to highlight the clinical relevance of the study. Otherwise, as the authors mention, this analysis render preeliminary results and requiere to expand the number of samples

Please return the manuscript within 60 days; if you cannot complete the modification within this time period, please contact me. If you do not wish to modify the manuscript and prefer to submit it to another journal, notify me immediately so that the manuscript may be formally withdrawn from consideration by Spectrum.

Revision Guidelines
To submit your modified manuscript, log into the submission site at https://spectrum.msubmit.net/cgi-bin/main.plex. Go to Author Tasks and click the appropriate manuscript title to begin. The information you entered when you first submitted the paper will be displayed; update this as necessary. Note the following requirements:

• Upload point-by-point responses to the issues raised by the reviewers in a file named "Response to Reviewers," NOT in your cover letter.
• Upload a compare copy of the manuscript (without figures) as a "Marked-Up Manuscript" file.
• Upload a clean .DOC/.DOCX version of the revised manuscript and remove the previous version.
• Each figure must be uploaded as a separate, editable, high-resolution file (TIFF or EPS preferred), and any multipanel figures must be assembled into one file.
• Any supplemental material intended for posting by ASM should be uploaded with their legends separate from the main manuscript. You can combine all supplemental material into one file (preferred) or split it into a maximum of 10 files with all associated legends included.

For complete guidelines on revision requirements, see our Submission and Review Process webpage. Submission of a paper that does not conform to guidelines may delay acceptance of your manuscript.

Data availability: ASM policy requires that data be available to the public upon online posting of the article, so please verify all links to sequence records, if present, and make sure that each number retrieves the full record of the data. If a new accession number is not linked or a link is broken, provide Spectrum production staff with the correct URL for the record. If the accession numbers for new data are not publicly accessible before the expected online posting of the article, publication may be delayed; please contact production staff (Spectrum@asmusa.org) immediately with the expected release date.

Publication Fees: For information on publication fees and which article types are subject to charges, visit our website. If your manuscript is accepted for publication and any fees apply, you will be contacted separately about payment during the production process; please follow the instructions in that e-mail. Arrangements for payment must be made before your article is published.

ASM Membership: Corresponding authors may join or renew ASM membership to obtain discounts on publication fees. Need to upgrade your membership level? Please contact Customer Service at Service@asmusa.org.

The ASM Journals program strives for constant improvement in our submission and publication process. Please tell us how we can improve your experience by taking this quick Author Survey.

Thank you for submitting your paper to Spectrum.

Sincerely,
Victor Gonzalez
Editor
Microbiology Spectrum

Reviewer #1 (Comments for the Author):

Study is interesting and show distribution of MRSA within a hospital in Jalisco.
First of all if authors want to know how changes

Reviewer #2 (Comments for the Author):

This manuscript analyzes the whole-genome sequences (WGS) of MRSA and MSSA strains isolated from patients at a tertiary care hospital in Mexico. The strains are characterized molecularly, and virulence factors, mobile genetic elements, antibiotic resistance, and a phylogenetic analysis are studied.

The following comments are made:

1. Provide more detail about the patients from whom the samples were taken: sex, age, type of infection, and sampling site. Specify the origin of the MSSA and MRSA strains, as these factors are important for understanding the types of strains used.
2. Also, describe the microbiological aspects, including how the strains were isolated and classified as MRSA and MSSA.
3. Indicate whether informed consent was obtained from the patients for sample collection and data processing.
4. Line 125. Indicate the composition of the lysis buffer.
5. Table 1 presents the MIC of various antibiotics for each strain. However, the Materials and Methods section does not explain how this was done. Please explain how the MIC was calculated.
6. Lines 255-257. Indicate the origin of the HA-MRSA strains and the CA-MRSA strains.
7. Table S6. Indicates cefoxitin disk diffusion. This method is also missing from the Materials and Methods section. Please add it.
8. Could you add a Supplementary Table listing the names and functions of the studied genes, such as those presented in Table S7.
9. Line 297. Indicate which strains contain the PVL genes: MSSA, HA-MRSA, or CA-MRSA.
10. Line 424. Explain the origin of the 39 MSSA strains, as only 13 MSSA strains are initially mentioned.
11. Line 451. Discuss the possible contamination of the hospital with a predominant MRSA strain.
12. Line 463. Indicate how a decrease in antibiotic pressure is evidenced.
13. Line 493. Discuss the type of patient and pathology from which these strains were isolated.
14. Lines 529-552. Discuss the presence of MDR-MSSA strains found in your sample and the data obtained.
15. Line 584. Discuss the presence of CA-MRSA strains and the type of patient and pathology from which they were isolated.

Response to Reviewers — Spectrum00948-26

Manuscript title (revised): Whole-Genome Sequencing of Staphylococcus aureus Isolates after a Decade-Long Decline in MRSA Prevalence at a University Referral Hospital in Guadalajara, Mexico: Clonal Concentration in MRSA and Greater Genetic Diversity in MSSA Corresponding author: Jaime Briseno-Ramirez (jaime.briseno@academicos.udg.mx) Date of resubmission: May 2026

We thank the Editor and the two Reviewers for their constructive critique. The four editorial concerns and the fifteen comments from Reviewer 2 have been addressed point by point in the revised manuscript and supplementary materials, with new analyses (whole-genome ANI, quantitative read-mapping against the N315 SCCmec II reference, focused chromosomal dotplot, mec-complex comparative table), a WGS-centered reframing of the Abstract and Conclusions, and a structural reorganization of the Results section so that the clinical risk-factor analysis is presented clearly as secondary and exploratory clinical context. Below we reproduce each comment verbatim and describe the corresponding change. Because manuscript line numbering differs between DOCX, PDF, and rendered outputs, we identify revised locations by manuscript section, table, figure, or supplementary item rather than by line number; line numbers appearing inside reviewer quotations are reproduced verbatim from the reviewers' comments.

We note up-front that the comments from Reviewer 1 appear to have been transmitted truncated (the only text we received is reproduced verbatim below and ends mid-clause). We respond on the basis of the text as transmitted in the decision letter and the Author Tasks page on the submission portal, and have inferred the likely thrust of the reviewer's question and addressed it substantively.

EDITOR COMMENTS

Editor Point 1 — Overstated SCCmec excision conclusion / "MRSA vulnerability"

> "The main claim of this manuscript is that the decline in MRSA prevalence within a hospital over a decade is likely due to molecular mechanisms involving the excision of the SCCmec cassette. This conclusion is supported by the phylogenomic analysis of a closely related MSSA (ST) nested with an MRSA clone. However, this observation is not sufficient to validate that this mechanism is associated with MRSA reduction or to conclude that MRSA is vulnerable. To make such statements, solid evidence should come from an ample

population analysis of the frequencies of MRSA/MSSA. Therefore, in my opinion, the conclusion is overstated and needs to be modified, as does the title."

Response. We are grateful to the Editor for this careful reading, and we accept the recommended changes in full. We would respectfully note, however, that at no point did the original manuscript propose SCCmec excision — or the SA023 observation — as the explanatory mechanism for the decade-long MRSA decline. The central descriptive claim of the manuscript was, rather, the contrast in clonal architecture between the two populations: a highly clonal MRSA population dominated by a single ST5-t895-SCCmec II clone (9/13 MRSA; Simpson's 1−D = 0.500) juxtaposed with a polyclonal MSSA reservoir of eleven sequence types and nine clonal complexes (Simpson's 1−D = 0.974). We now present this structural contrast more conservatively as a genomic feature of the contemporary low-prevalence population, quantified using Simpson's diversity index and Pielou's evenness (Results, "Molecular Typing and Clonal Structure"; Table S12). The SA023 phylogenomic observation was offered as one molecularly grounded illustration consistent with that broader contrast, never as the sole or primary explanatory mechanism, and we did not intend any single isolate to carry population-level causal weight. We nevertheless recognise on re-reading that the Abstract Conclusions, Importance, Discussion, and Conclusions sections did permit the stronger reading the Editor identified, and we have therefore softened every relevant passage: the Discussion now describes the key point as "a single phylogenomic observation consistent with SCCmec excision in CC5", the title has been substantially reframed (see below), and explicit caveats now state that population-level surveillance is required to test the wider hypothesis formally. We fully agree that the single SA023 observation cannot, on its own, establish ccrAB-mediated SCCmec excision as a population-level driver of the decline, and we have made this distinction unambiguous everywhere it appears.

Changes in the revised manuscript.

- Title. We have substantially reframed the title to align it with the editor's request and with the descriptive nature of our findings. We recognize that the original "Clonal Vulnerability" framing risked being read as a population-level causal claim that our single-isolate observation cannot support, and we appreciate the editor's prompting us to clarify this. The revised title is "Whole-Genome Sequencing of Staphylococcus aureus Isolates after a Decade-Long Decline in MRSA Prevalence at a University Referral Hospital in Guadalajara, Mexico: Clonal Concentration in MRSA and Greater Genetic Diversity in MSSA". Four changes from the original deserve specific note: (i) the word "Vulnerability" has been removed in its entirety; (ii) the active verb "Reveals" has been removed and the title is now structured descriptively (study setup before the colon, findings after the colon), so that no causal claim is implied; (iii) "after a Decade-Long Decline in MRSA Prevalence" replaces the original "Dur-

ing a Decade-Long Prevalence Decline" framing to avoid the unintended reading that sequencing itself spanned a decade; the cohort represents contemporary 2024–2025 isolates sampled after the documented institutional decline; and (iv) the location is anchored to a university referral hospital in Guadalajara rather than to "Western Mexico", avoiding any implication that this single-center study is regionally representative. The new title foregrounds the two parallel descriptive findings of the study — clonal concentration in MRSA and broad genetic diversity in MSSA — and presents the decade-long MRSA decline as the temporal context of the cohort rather than a phenomenon the manuscript claims to explain. The corresponding language in the Abstract Conclusions, Discussion, Limitations, and Conclusions sections has been adjusted in parallel: every previous use of "clonal vulnerability" has been replaced with descriptive equivalents ("clonal concentration", "highly clonal MRSA vs diverse MSSA polyclonal structure", "hypotheses for future longitudinal testing") so that the manuscript now describes what we observed without asserting a causal mechanism for the wider decline.

- Abstract rewritten to foreground WGS. The Methods now emphasize assembly, species confirmation, MLST/spa/SCCmec typing, resistance and virulence profiling, mobile-element analysis, genotype–phenotype concordance, and contextual phylogenomics; the matched case-control analysis is explicitly described as secondary. The Abstract Conclusions now state that WGS identifies lineage-specific resistance/mobile-element architectures and one observation consistent with SCCmec excision, generating hypotheses for longitudinal surveillance rather than establishing a mechanism for MRSA decline.
- Importance rewritten to keep the main contribution genomic and descriptive: the study provides a WGS-based baseline for longitudinal MRSA surveillance, and the clinical risk-factor analysis is included as exploratory context rather than primary evidence for the genomic conclusions.
- Discussion. The previous overstatement has been replaced with the more cautious wording: "a single phylogenomic observation consistent with SCCmec excision in CC5".
- Discussion. The four-part evidence list is now framed as supporting a working hypothesis rather than a documented mechanism, and the closing caveat states explicitly that "Because this remains a single-isolate observation, generalization to the wider MRSA decline at this institution requires confirmation in larger longitudinal collections and ideally long-read sequencing to resolve the excision boundaries and the plasmid topology of the released elements."
- Conclusions. The final paragraph now states that the findings "define genomic patterns that warrant longitudinal follow-up but do not establish a mechanism for the decline" and that a larger genomic surveillance study spanning high- and low-prevalence eras, ideally with long-read sequencing, is required to test these hypotheses formally. The secondary case-control analysis is presented only as clinical context.

- New Figure S4 (read-coverage track of SA023 against N315 SCCmec II) and new Figure S5 (focused chromosomal dotplot SA023 vs sister CC5-MRSA SA015) provide quantitative evidence that the cassette-specific ccrA–mecA core is 94.8% uncovered in SA023 reads while the chromosomal attB locus is intact and collinear with the sister MRSA — strengthening the genomic basis for the SA023 observation without overstating its population-level meaning.
- New Table S17 (mec-complex comparative analysis) shows that all 9 sibling CC5-MRSA carry mecA+mecR1+mecI+ccrA+ccrB, while SA023 alone among the CC5 isolates carries zero of these mec-complex genes, a pattern consistent with the excision interpretation for this single isolate.

Editor Point 2 — Species identification by ANI rather than Kraken2

> "Species identification is commonly achieved by calculating the whole-genome average nucleotide identity (ANI), followed by core phylogeny. Kraken is best used in metagenomics, and its use with genomes is not reliable at the species level. Furthermore, Kraken does not provide identity percentages. It is unclear how this percentage was obtained (l-153). We suggest using ANI or validating Kraken profiling as adequate for species identification."

Response. We agree that whole-genome ANI is the appropriate method for species-level identification of assembled genomes. We have replaced Kraken2 as the primary species-ID method with FastANI v1.34 (Jain et al., 2018) computed against five S. aureus reference genomes (NCTC8325, N315, USA300 FPR3757, Mu50, MW2) and S. epidermidis RP62A as outgroup. All 26 isolates returned ≥97.28% ANI to NCTC8325 (mean 98.7%, range 97.28–99.79%), all returned ≥95% ANI to at least one S. aureus reference, and S. epidermidis RP62A returned no significant ANI for any of the 26 query genomes — consistent with confident within-species identification. We acknowledge that the percentage we previously reported from Kraken2 reflected the proportion of reads classified as S. aureus rather than nucleotide identity; we have removed this conflation. Kraken2 is now retained as a complementary contamination screen.

Changes in the revised manuscript.

- Methods, "De novo Assembly and Annotation" rewritten to describe FastANI v1.34 as the primary species-ID method, with the six reference genomes listed by GCF accession; ANI thresholds quoted; Kraken2 retained explicitly as a contamination screen.
- Results, "Sequencing and Assembly" rewritten. The revised text states: "All assemblies passed species confirmation by four convergent methods: whole-genome ANI (≥97.28% to NCTC8325, ≥95% to at least one S. aureus reference; Table S2), Kraken2 read classification (≥98% S. aureus), MLST against the species-specific PubMLST scheme, and spa typing."

- New Table S2 added: per-isolate ANI to NCTC8325, best-match reference, and best-ANI value, plus footnote explaining the five samples below 98.5% to NCTC8325 (SA007 CC152, SA008 CC22, SA015 CC5, SA017 novel CC22, SA025 CC30 — all within-species variation explained by phylogenetic distance from the NCTC8325 reference).
- References updated with Jain et al., 2018 (Nature Communications 9:5114).

Editor Point 3 — Fitness claims without experimental measurement

> "Although fitness associated with the class of SCCmec cassette has been reported and pointed out in the manuscript, no experiment was performed to evaluate the fitness in the collection of strains used in the manuscript. I am not sure you can extrapolate the […]. The genomic background of the strains, such as genome size and alleles, should also play a role in growth rates and overall fitness. I suggest reviewing the corresponding statements."

Response. We agree. The original manuscript described a "fitness cost" in absolute terms ("HIGH fitness cost", "LOW fitness cost") without performing growth or competition assays in the studied strains; we have re-framed every such claim as a predicted metabolic burden inferred from cassette size and gene content, and have added an explicit Limitations statement that no in vitro fitness assays were conducted at this institution. The reviewer's broader point on chromosomal background and allele effects is also acknowledged in Limitations.

Changes in the revised manuscript.

- Subsection title changed from "Fitness-Associated Genomic Markers" to "Genomic Markers Related to SCCmec Burden and Colonization".
- Results, "Genomic Markers Related to SCCmec Burden and Colonization." The old "HIGH/LOW fitness cost" language has been replaced with cassette-burden wording. The current Results state: "All nine CC5-SCCmec II isolates carried a ~53 kb cassette (predicted higher metabolic burden based on cassette size and gene content; not measured experimentally in this study), while the four CC8/CC88-SCCmec IV isolates harbored the smaller ~24 kb element (predicted lower burden)."
- Abstract Results. The previous fitness-cost framing was removed. The Abstract now describes lineage-specific resistance and mobile-element architectures: "CC5-MRSA carried SCCmec II with ermA/aadD and QRDR mutations, while SCCmec IV MRSA belonged to CC8/CC88 and lacked ACME/COMER."
- Discussion. The previous "fitness cost" wording has been replaced with: "the inferred metabolic burden of SCCmec II, supported by prior experimental literature on SCCmec-associated fitness effects and psm-mec-mediated regulation [11, 62] but not measured in this study"; the subse-

quent fitness statement is explicitly tied to published SCCmec-associated fitness costs [11].

- Limitations. New sentence: “We did not perform in vitro growth or competition assays; references to fitness effects or metabolic burden rely on prior experimental literature for SCCmec-associated fitness effects and psm-mec-mediated regulation [11, 62], and direct fitness measurements at this institution would be needed to confirm that the published burden estimates apply to local lineages. Likewise, allele-level variation within shared genes and chromosomal background effects also influence fitness and were not modelled here.”

Editor Point 4 — “Risk Factors” section appears disconnected; move to first Results subsection

> “The section ‘Risk Factors for MRSA Infection’ appears somewhat disconnected from the main purpose of the manuscript. I suggest moving it to the first section of the Results to highlight the clinical relevance of the study. Otherwise, as the authors mention, this analysis render preeliminary results and requiere to expand the number of samples”

Response. Implemented. The risk-factor analysis is now the first subsection of the Results, integrated with a brief Cohort Description paragraph (drawn from the new Table S6) so that readers encounter the clinical context before the genomic findings. We have also adopted the Editor’s suggested phrasing, adding “Given the small sample size, these results are preliminary and require validation in a larger cohort” at the close of the subsection.

Changes in the revised manuscript.

- Results §3.1 (NEW): “Cohort Description and Risk Factors for MRSA Infection” — formerly the last subsection of Results; combines a short demographic/cohort paragraph with the matched case-control analysis, OR table, Firth aORs, exploratory mortality data, and the new “preliminary; require validation” caveat.
- Renumbered subsequent subsections (Sequencing and Assembly is now §3.2, etc.). A bridging clause was added to the new §3.2 opening: “All 26 isolates from the cohort described above yielded high-quality assemblies…”.
- Reviewer 2 points 6, 13, 15 are now addressed in part by the new Cohort Description and the supporting Table S6 / Table S7 (HA/CA classification per CDC 2013 narrative criteria).

REVIEWER 1 COMMENTS

> "Study is interesting and show distribution of MRSA within a hospital in Jalisco. First of all if authors want to know how changes"

Note on reviewer text. The text reproduced above is the complete content of the Reviewer 1 comments as transmitted to us through the decision letter and the Author Tasks page on the submission portal. The single complete sentence is followed by an incomplete clause that ends mid-thought after the words "how changes" without a verb, object, or terminal punctuation, indicating that part of the reviewer's submission was lost in transmission. We address the comment as transmitted, offering an inferred response based on the most plausible reading of the truncated clause and on related concerns raised by the Editor and Reviewer 2.

Inferred concern. We interpreted the truncated sentence as a sampling-design concern: if the aim is to understand how MRSA prevalence changed across the 9.5-year decline, a contemporary 2024–2025 isolate set can describe the low-prevalence population but cannot, by itself, reconstruct the decade-long temporal trajectory.

Response. This is a fair concern, and it converges with Editor Point 1. The manuscript's central message is descriptive: that the contemporary MRSA population at this institution, sampled during the low-prevalence phase of the previously published 9.5-year prevalence decline [22], is highly clonally concentrated (ST5-t895-SCCmec II = 9/13, 69.2%; Simpson's 1−D = 0.500), whereas the MSSA population is highly diverse (Simpson's 1−D = 0.974). We do not sequence isolates from the high-prevalence era — and we now state this limitation explicitly. The revised text emphasises that the genomic snapshot describes the clonal architecture during the low-prevalence phase and does not itself demonstrate the temporal mechanism of decline.

Changes in the revised manuscript (in addition to those listed under Editor Point 1).

- Methods, Study Design retains the explicit statement that the 26 isolates represent a stratified random sample of the post-peak (2024–2025) period.
- Discussion softened to "The genomic data reveal a contemporary low-prevalence population characterized by marked MRSA clonal concentration in a pandemic clone carrying a cassette associated in prior literature with higher metabolic burden, a single phylogenomic observation consistent with SCCmec excision in CC5, and genetically diverse MSSA carrying substantial virulence determinants."
- Limitations. The revised text states that isolates represent a single time point during the low-prevalence period and that, without WGS data from the high-prevalence era (~2016), evidence for clonal replacement remains indirect.

- Conclusions explicitly state that a larger genomic surveillance study spanning high- and low-prevalence eras, ideally with long-read sequencing, is required to test these hypotheses formally.

If the complete Reviewer 1 text contains additional points that we have not anticipated above, we will address each in a focused supplementary response on receipt.

REVIEWER 2 COMMENTS

> "This manuscript analyzes the whole-genome sequences (WGS) of MRSA and MSSA strains isolated from patients at a tertiary care hospital in Mexico. The strains are characterized molecularly, and virulence factors, mobile genetic elements, antibiotic resistance, and a phylogenetic analysis are studied. The following comments are made:"

We thank Reviewer 2 for the careful reading and the fifteen specific points, which have led to a substantial strengthening of the Methods section, new and expanded supplementary tables for clinical context, gene/function annotation, and SCCmec evidence, and a sharper attribution of clinical context to the molecular findings.

R2.1 — Patient demographics: sex, age, infection type, sampling site, MSSA/MRSA origin

> "Provide more detail about the patients from whom the samples were taken: sex, age, type of infection, and sampling site. Specify the origin of the MSSA and MRSA strains, as these factors are important for understanding the types of strains used."

Response. Implemented. The new Table S6 (Patient demographics and clinical characteristics) provides age, sex, department, specimen, diagnosis, MRSA/MSSA status, ST, CC, spa type, SCCmec, ANI to NCTC8325, and species call for all 26 sequenced isolates. The information is also summarised in the new "Cohort Description" paragraph at the start of Results (§3.1).

R2.2 — Microbiological methods for strain isolation and MRSA/MSSA classification

> "Also, describe the microbiological aspects, including how the strains were isolated and classified as MRSA and MSSA."

Response. Implemented. We added a new Methods subsection "Strain Isolation, Phenotypic Identification, and MRSA/MSSA Classification" describing the routine workflow (sheep blood agar 35 °C, 18–24 h; coagulase + DNase

+ VITEK 2 GP card for species ID; preservation in 20% glycerol at −80 °C). MRSA classification required concordant resistance in both (i) cefoxitin disk diffusion (30 μg disk, ≤21 mm = R) and (ii) oxacillin broth microdilution (VITEK 2; ≥4 μg/mL = R) per CLSI 2024 (M100, 34th ed.). Discrepant cases were resolved by genotypic mecA/mecC status with read-mapping confirmation against AB245468.1. S. aureus ATCC 29213 and ATCC 43300 were used as QC strains.

R2.3 — Informed consent

> "Indicate whether informed consent was obtained from the patients for sample collection and data processing."

Response. The Ethics Approval section already states this; we have made it more explicit:

> "Patient consent was waived by the Institutional Ethics Committee (CUTLAJO/DS/CEICS/07/26, 2026-02-25) due to the retrospective design and minimal-risk nature of the study, and because all data were analyzed in fully de-identified form (study codes SA001–SA027); no individually identifiable patient information was accessed by the genomic team."

R2.4 — Composition of the lysis buffer

> "Line 125. Indicate the composition of the lysis buffer."

Response. Implemented. The DNA Extraction subsection now describes the lysozyme pre-treatment buffer composition (20 mg/mL lysozyme in 50 mM Tris-HCl pH 8.0, 50 mM EDTA, 1.2% Triton X-100) and states that the proprietary MagDEA Dx SV lysis buffer is a "chaotropic guanidinium thiocyanate-based denaturant with anionic detergent and EDTA-buffered carrier."

R2.5 — MIC determination method

> "Table 1 presents the MIC of various antibiotics for each strain. However, the Materials and Methods section does not explain how this was done. Please explain how the MIC was calculated."

Response. Implemented. We added a new Methods subsection "Antimicrobial Susceptibility Testing and MIC Determination" describing broth microdilution by VITEK 2 (AST-P607 panel, bioMérieux) for the agents in Table 1, with cefoxitin disk diffusion (30 μg) on Mueller-Hinton agar interpreted per CLSI M100 34th ed. (2024); D-test for inducible MLS_B; mupirocin E-test (high-level R if ≥512 μg/mL). QC strains S. aureus ATCC 29213 and E. faecalis ATCC 29212 were included in every batch.

R2.6 — HA-MRSA vs CA-MRSA origin

> "Lines 255-257. Indicate the origin of the HA-MRSA strains and the CA-MRSA strains."

Response. Implemented. The new Table S7 classifies each isolate as healthcare-associated (HA), community-associated (CA), or indeterminate per CDC 2013 narrative criteria (no admission timeline available for some endocarditis cases). Distribution: MRSA 4 HA / 5 CA / 4 indeterminate; MSSA 3 HA / 5 CA / 5 indeterminate. The Results direct readers to Table S7 for HA/CA classification, and the Discussion comments on the CC8-SCCmec IV isolates as a PVL-positive secondary CA-MRSA lineage distinct from USA300/USA300-LV.

R2.7 — Cefoxitin disk diffusion method (Table S6)

> "Table S6. Indicates cefoxitin disk diffusion. This method is also missing from the Materials and Methods section. Please add it."

Response. Added to the new Methods subsection on Antimicrobial Susceptibility Testing (see R2.5 above). The revised text states that cefoxitin disk diffusion was performed with a 30 μg disk on Mueller-Hinton agar, with zone diameters read at 16–18 h and interpreted per CLSI M100, 34th edition (2024), as susceptible (≥22 mm) or resistant (≤21 mm). QC strains and breakpoint citations are explicit.

R2.8 — Supplementary table of gene names and functions (style of Table S7)

> "Could you add a Supplementary Table listing the names and functions of the studied genes, such as those presented in Table S7."

Response. Implemented as the new Table S20 ("Catalogue of resistance, virulence, and anti-phage defense genes/systems"). The combined catalogue includes 188 entries spanning antimicrobial-resistance determinants, virulence-associated genes, stress/resistance markers, and anti-phage defense systems. DefenseFinder identified 23 defense-system subtypes across the cohort (Table S19). Each entry lists gene/system symbol, type, class, subclass, product/function, and database source. The full catalogue is provided as a tab-separated file (`tableS20_genes_function.tsv`) with the supplementary materials.

R2.9 — PVL strain attribution: MSSA, HA-MRSA or CA-MRSA

> "Line 297. Indicate which strains contain the PVL genes: MSSA, HA-MRSA, or CA-MRSA."

Response. Implemented. The Virulence Factor Profiles subsection now explicitly attributes each PVL+ isolate by category: PVL genes were detected

in SA005 (CC88-MRSA, SCCmec IV; CA), SA006 (CC8-MRSA, SCCmec IV; CA), SA018 (CC8-MRSA, SCCmec IV; CA), SA027 (CC8-MRSA, SCCmec IV; CA, burn-wound), SA007 (CC152-MSSA; CA, burn-wound), and SA010 (CC8-MSSA; CA), establishing that PVL positivity comprised four CA-MRSA isolates and two CA-MSSA isolates.

R2.10 — Origin of the 39 MSSA controls

> "Line 424. Explain the origin of the 39 MSSA strains, as only 13 MSSA strains are initially mentioned."

Response. Clarified and expanded. All 39 controls are from the same institution (Antiguo Hospital Civil de Guadalajara), drawn from routine hospital microbiology cultures during the same collection window. The Methods now state that the controls comprise 13 MSSA isolates from the WGS cohort plus 26 additional routine hospital MSSA isolates from the institutional Laboratory Information System used only for epidemiological matching and not subjected to whole-genome sequencing. The revised Table S4 now provides a de-identified analytic registry for the complete case-control set (13 MRSA cases and 39 MSSA controls), rather than a controls-only origin table. To keep the supplement readable, Table S4 is split into S4a–S4c: S4a reports source and matching/balance variables, S4b reports the variables used in the Firth models and the main clinical outcomes, and S4c reports additional covariates used in univariable comparisons. We also made the clinical time windows explicit: prior hospitalization within 12 months, prior antibiotic exposure within 90 days, and surgery within 30 days. Patient names and medical-record numbers are not exported.

In the course of preparing this Response we re-audited the matching procedure against the original data-collection workbook and rewrote the Methods text to describe frequency matching at the cohort level rather than as individual triplet matching. Using the corrected clinical-ID mapping applied throughout the revised manuscript, the 13 cases and 39 controls did not differ significantly in age (median 46 vs 41 years; Mann–Whitney P=0.32; Welch's t P=0.35), sex (92.3% vs 76.9% male; Fisher's exact P=0.42), or sampling period (both within Q1–Q2 2025; Mann–Whitney on the de-identified culture-day index P=0.83). The Methods text now reports these test statistics directly so that readers can verify the empirical balance of the matched cohort. The unconditional Firth penalised regression used for the multivariable analysis does not require individual case-control triplet conditioning, so the odds-ratio estimates remain valid under cohort-level frequency matching.

R2.11 — Possible hospital contamination by predominant MRSA

> "Line 451. Discuss the possible contamination of the hospital with a predominant MRSA strain."

Response. Added to the Discussion. The 9 CC5-MRSA-SCCmec II isolates

differ by 0–20 cgMLST allelic differences (Table S18; Fig. S6) and pairwise SNP distances of 3–58 (Table S13; Supplementary Data S2; the closest pairs are SA004–SA019 at 3 SNPs and SA004–SA002 at 4 SNPs). We now state that these low distances are compatible with recent clonal dissemination and possible nosocomial circulation of an endemic New York/Japan MRSA lineage. We also explicitly caveat that this study did not include environmental sampling, healthcare-worker carriage screening, or device-tracing data; therefore, hospital contamination or transmission pathways cannot be formally inferred.

R2.12 — Evidence of decreased antibiotic pressure

> "Line 463. Indicate how a decrease in antibiotic pressure is evidenced."

Response. Clarified. We do not directly measure antibiotic-use trends or antibiotic selection pressure in this study. The revised Discussion now states that regional Mexican molecular/susceptibility surveillance and the previously described institutional antimicrobial-stewardship program provide only indirect epidemiologic context for a changing MRSA ecology [19, 22], and that antibiotic use and antibiotic selection pressure were not measured longitudinally in the present WGS cohort. This keeps the decreased-selection-pressure explanation explicitly inferential rather than presented as a demonstrated finding.

R2.13 — Patient type and pathology of these strains

> "Line 493. Discuss the type of patient and pathology from which these strains were isolated."

Response. Implemented. The Results now identify the PVL-positive isolates by MRSA/MSSA status and HA/CA category, including four CA-MRSA isolates and two CA-MSSA isolates. Full patient demographics, specimen source, infection/pathology, and HA/CA context are provided in Tables S6 and S7, and the Discussion identifies the CC8-SCCmec IV isolates as a PVL-positive secondary CA-MRSA lineage distinct from USA300/USA300-LV.

R2.14 — MDR-MSSA strains and obtained data

> "Lines 529-552. Discuss the presence of MDR-MSSA strains found in your sample and the data obtained."

Response. Implemented. We added a Discussion paragraph explicitly addressing the MDR-MSSA cases identified in our cohort. SA013 showed phenotypic non-susceptibility to penicillin, erythromycin, and tetracycline together with multiple resistance determinants; SA017 represented a novel CC22 MSSA background with genotypic determinants that overpredicted tetracycline resistance; and SA020 represented a CC25 BORSA phenotype (cefoxitin negative, oxacillin MIC ≥4 μg/mL with blaZ-associated β-lactamase hyperproduction, no

mecA/mecC). The paragraph now states that MDR-MSSA can remain clinically important even when isolates are methicillin-susceptible, supports careful resistance phenotyping and genotypic follow-up where available, and cites Latin American surveillance studies (Diekema et al., 2019; Seas et al., 2018) as context for regional burden and heterogeneity rather than as direct evidence for routine MDR genotyping.

R2.15 — CA-MRSA strains: patient type and pathology

> "Line 584. Discuss the presence of CA-MRSA strains and the type of patient and pathology from which they were isolated."

Response. Implemented. The Results now identify the CA-MRSA/PVL+ isolates as SA005 (CC88-MRSA, SCCmec IV; CA), SA006 (CC8-MRSA, SCCmec IV; CA), SA018 (CC8-MRSA, SCCmec IV; CA), and SA027 (CC8-MRSA, SCCmec IV; CA, burn-wound). Full patient profile and pathology information are provided in Tables S6 and S7. The Discussion further describes the CC8-SCCmec IV isolates as a PVL-positive secondary CA-MRSA lineage distinct from USA300/USA300-LV.

CLOSING SUMMARY

The revised manuscript is shorter on overstatement and longer on quantitative evidence. The four editorial concerns are addressed by (i) softening the title, abstract, and conclusions so that the SA023 SCCmec pattern is described as a single observation consistent with cassette excision rather than a population-level driver, (ii) replacing Kraken2 as the primary species-ID method with whole-genome ANI (FastANI v1.34), (iii) re-framing every fitness claim as a literature-derived inference of metabolic burden, and (iv) moving the Risk Factors / clinical analysis to the first Results subsection. The fifteen Reviewer 2 comments are addressed in detail and have produced new or expanded clinical/cohort tables (Tables S4, S6, S7, and S8), a gene/function catalogue (Table S20), a mec-complex comparative table (Table S17), two SCCmec support figures (Figures S4 and S5), and a substantially expanded Methods section.

We thank the Editor and Reviewers for the time invested in this manuscript, and we hope the revision now satisfies their concerns. We remain at the editorial office's disposal to address any residual point.

— On behalf of all co-authors, Jaime Briseno-Ramirez, M.D., MSc (corresponding author)

Re: Spectrum00948-26R1 (Whole-Genome Sequencing of *Staphylococcus aureus* Isolates after a Decade-Long Decline in MRSA Prevalence at a University Referral Hospital in Guadalajara, Mexico: Clonal Concentration in MRSA and Greater Genetic Diversity in MSSA)

Dear Dr. Jaime Briseno-Ramirez:

Thank you for your responses and clarifications provided to reviewers and editor.

Your manuscript has been accepted, and I am forwarding it to the ASM production staff for publication. Your paper will first be checked to make sure all elements meet the technical requirements. ASM staff will contact you if anything needs to be revised before copyediting and production can begin. Otherwise, you will be notified when your proofs are ready to be viewed.

Data Availability: ASM policy requires that data be available to the public upon online posting of the article, so please verify all links to sequence records, if present, and make sure that each number retrieves the full record of the data. If a new accession number is not linked or a link is broken, provide production staff with the correct URL for the record. If the accession numbers for new data are not publicly accessible before the expected online posting of the article, publication may be delayed; please contact ASM production staff immediately with the expected release date.

Publication Fees: For information on publication fees and which article types have charges, please visit our website. We have partnered with Copyright Clearance Center (CCC) to collect author charges. If fees apply to your paper, you will receive a message from no-reply@copyright.com with further instructions. For questions related to paying charges through RightsLink, please contact CCC at ASM_Support@copyright.com or toll free at +1-877-622-5543. CCC makes every attempt to respond to all emails within 24 hours.

ASM Membership: Corresponding authors may join or renew ASM membership to obtain discounts on publication fees. Need to upgrade your membership level? Please contact Customer Service at Service@asmusa.org.

Embargo Policy: A press release may be issued as soon as the manuscript is posted on the Spectrum Latest Articles webpage. The corresponding author will receive an email with the subject line "ASM Journals Author Services Notification" when the article is available online.

The ASM Journals program strives for constant improvement in our submission and publication process. Please tell us how we can improve your experience by taking this quick Author Survey.

Thank you for submitting your paper to Spectrum.

Sincerely,
Victor Gonzalez
Editor
Microbiology Spectrum
